# Under controlled conditions, lower concentrations of aqueous extracts of *Acroptilon repens* are needed to inhibit seed germination and seedling growth of weeds than crops

**DOI:** 10.1371/journal.pone.0354454

**Published:** 2026-08-27

**Authors:** Fatemeh Ahmadnia, Elham Samadi-Kalkhoran, Mohammad Taghi Alebrahim, Dana R. MacGregor

**Affiliations:** 1 Department of Plant Production and Genetics, Faculty of Agriculture and Natural Resources, University of Mohaghegh Ardabili, Ardabil, Iran; 2 Plant Protection Research Department, Hamedan Agricultural and Natural Resources Research and Education Center, Agricultural Research, Education, and Extension Organization (AREEO), Hamedan, Iran; 3 Rothamsted Research, West Common, Harpenden, Hertfordshire, United Kingdom; Canakkale Onsekiz Mart University, TÜRKIYE

## Abstract

Preventing weeds from hindering agricultural production often relies on chemical inputs; however, these inputs can be ineffective, increase costs, or harm the environment. Natural products, particularly plant extracts, are being explored as potential sources for developing eco-friendly bioherbicides that replace or reduce the use of chemical herbicides. In this study, we examined to what degree aqueous extract from the allelopathic weed *Acroptilon repens* L. (Russian knapweed) was able to influence seed germination and seedling growth metrics *in vitro*. Uniquely, our study compared the impacts of *A. repens* extract on three weedy species (*Sinapis arvensis* L., *Amaranthus retroflexus* L., and *Avena fatua* L.), a multipurpose species (*Secale cereale* L.), and two crop species (*Triticum aestivum* L., and *Vicia faba* L.) in side-by-side comparisons. As expected, a significant (*P* ≤ 0.01), linear, and concentration-dependent decline in germination metrics and seedling growth was observed after application of the extract, while mean germination time (*P* > 0.05) was not changed. Interestingly, the weedy species were affected at lower concentrations compared to the crops. Germination was fully inhibited for *S. arvensis* ≥ 40 g L^-1^, *A. retroflexus* ≥ 80 g L^-1^, *A. fatua* ≥ 120 g L^-1^, *T. aestivum* ≥ 140 g L^-1^, and *S. cereale* ≥ 160 g L^-1^ of *A. repens* extract. These findings are based on *in vitro* assays and do not directly demonstrate allelopathic activity under field or soil conditions. In addition to existing data on the phytotoxic compounds produced by *A. repens,* our untargeted GC-MS profiling putatively identified several compounds that warrant further exploration, including phenolic compounds, a sesquiterpene (caryophyllene oxide), and lipid-derived constituents such as fatty acids and sterols. Although comprehensive ecological and agronomic assessments are needed prior to field application and further research is needed to isolate and confirm the bioactivity of these putatively identified secondary metabolites, these data suggest that *A. repens* aqueous extract can inhibit germination and early seedling development of problematic weeds more strongly than crops.

## Introduction

Wheat (*Triticum aestivum* L.) and fava bean (*Vicia faba* L.) are recognized as essential pillars of global food security and sustainable nutrition. *Triticum. aestivum* is a primary source of calories, protein, and key micronutrients for billions of individuals worldwide [1]. Moreover, *V. faba* is a protein-rich legume that enhances soil fertility through atmospheric nitrogen fixation and is widely used in crop rotations across diverse agricultural systems [2]. However, both face serious threats from uncontrollable weed infestations [3,4]. Weed management strategies often rely on chemical inputs, especially herbicides, to mitigate the adverse effects that weeds have on crop yields [5]. The overuse and indiscriminate application of conventional herbicides have led to herbicide resistance [6] and caused significant multiple environmental issues, including the degradation of agricultural ecosystems, the buildup of chemical residues, and contamination of vital resources such as air, water, and soil [7,8]. Successful sustainable agriculture depends on developing safer and more effective alternatives to conventional herbicides [9]. The use of allelopathic plant extracts as substitutes for synthetic herbicides is a promising approach [10]. Plant-based allelopathic compounds are typically released through leachates, secretions, or decomposition [11,12]. These compounds impact plant physiology by affecting photosynthesis, respiration, and hormone production [13]. They can disrupt cell division, hormone biosynthesis, and transport [14], alter membrane permeability [15], and influence water relations, leading to reduced growth [16,17]. Allelopathic compounds can cause root necrosis, poor germination, discoloration, and diminished reproductive capacity [18].

Suppressing weed seed germination is key to sustainable management of weeds within the agricultural ecosystem. Unchecked weed seedlings compete for resources such as water, nutrients, and light, and this competition reduces yields by 20–50% in staple crops including *T. aestivum* and *V. faba* [19,20]. Weed seedlings in the agri-ecosystem also increase management costs as they require postemergence interventions, which can promote the development of herbicide-resistant weeds [21]. Strategically suppressing weed seed germination through seedbank depletion, phytotoxic interference, or targeted soil ecology management can reduce reliance on reactive control methods and strengthen long-term agricultural resilience.

Russian knapweed, a perennial herb in the *Asteraceae* family, is known by several scientific names, including *Acroptilon repens*, *Rhaponticum repens*, *Centaurea repens*, and *Leuzea repens*. It is native to Iran, western Turkestan, Mongolia, Turkey, and Asia Minor [22]. Even as early as the 1960s [23], *A. repens*, then called *Centaurea repens*, was known as a highly competitive plant capable of forming dense patches that suppress the growth of other species. Early research on this plant showed that soil infested by *A. repens* could inhibit growth of tomato plants, and aqueous extracts from *A. repens* leaves and stems strongly inhibited germination of turnips [23]. This work has since been extended, and numerous studies have shown that aqueous extracts of *Acroptilon repens* reduce germination and seedling growth parameters across a wide range of species, including problematic weeds such as bladder campion (*Silene vulgaris*), cleavers (*Galium aparine*), common purslane (*Portulaca oleracea*), common yellow marrow (*Abutilon theophrasti*), lamb’s quarters (*Chenopodium album*), redroot pigweed (*Amaranthus retroflexus*), and velvetleaf (*Abutilon theophrasti*), as well as crop species including barley (*Hordeum vulgare* ‘Vantage’), tomato (*Solanum lycopersicum* ‘Firestell’) and wheat (*Triticum aestivum*) [23–26]. Like many of these studies, the present study evaluates its effects using controlled *in vitro* germination assays and therefore addresses phytotoxic potential rather than allelopathy in a true ecological sense. Despite this wealth of literature, very few of these studies compare the efficacy of target and non-target plants as we have done here. Although not all reports have stood the test of time (such as the retracted paper by Stermitz et al. [27]), the literature reports that *A. repens* releases a complex mixture of allelochemicals, including indole derivatives [23], sesquiterpene lactones such as repin, acroptilin, and hyrcanin [28,29], and five polyacetylenic compounds VIII–XIV [30,31]. We also used untargeted GC-MS analysis to putatively identify compounds that may underpin the biological activity we observe.

As novel methods to manage otherwise uncontrollable weeds are desperately needed and *A. repens* shows great potential to repress seed germination and seedling growth in a variety of species, we assessed whether aqueous extract from *A. repens* was able to alter seed germination and seedling growth metrics in side-by-side comparisons between weeds and the crops in which they are likely to be found. We assessed three weeds, redroot pigweed (*Amaranthus retroflexus* L.), wild oat (*Avena fatua* L.), wild mustard (*Sinapis arvensis* L.), and the mixed-use species wild rye (*Secale cereale* L.) and compared these against two crops, wheat (*Triticum aestivum* L.) and fava bean (*Vicia faba* L.)*.* Specifically, our research determined the concentration needed to inhibit germination of each species independently and identified which growth parameters were affected. When exploring allelopathic potential, many studies, including the present work, rely on controlled *in vitro* germination assays, which assess phytotoxic potential rather than allelopathic interactions under ecological conditions. Therefore, caution is required when extrapolating laboratory findings to field-based weed management scenarios.

The weedy species we have chosen hinder crop growth and reduce yields because of their rapid growth, prolific seed production, and ability to germinate under diverse environmental conditions. *Amaranthus retroflexus*, *S. cereale*, *A. fatua*, and *S. arvensis* are common and troublesome weeds across various cropping systems, including in *T. aestivum* [32]. *Avena fatua* is also problematic in *V. faba* [32]. *Secale cereale* is both a weed and a cover crop. It is herbicide-resistant and problematic in parts of the U.S. [33]; however, it is widely used to increase soil health and suppress other less competitive weeds [34]. Together, these species represent a biologically and agronomically relevant spectrum for comparative analyses of how *A. repens* aqueous extract influences germination and early seedling growth under controlled conditions.

## Materials and methods

### Experimental design and equations used for the analysis

Vegetative tissues of *A. repens*, including leaves and stems, were collected before flowering from wheat fields affected by this weed located at a latitude of 38°13′ N and 48°18′ E longitude, and an elevation of 1367 meters above sea level. The collected samples were thoroughly dried at room temperature (25 ± 2°C) for 30 days and pooled into a single bulk sample, which was then finely ground to ensure homogeneity [35]. This single homogenized batch of dried plant material was used for all experiments.

Aqueous extracts of *A. repens* were prepared from this bulk material via the maceration method described by Ahmadnia et al. [36]. Briefly homogenized plant material was mixed at a ratio of 0:1, 20:1, 40:1, 60:1, 80:1, 100:1, 120:1, 140:1, 160:1, 180:1, or 200:1 w/v in distilled water, followed by shaking for 24 hours to allow soluble metabolites to elute, filtration through Whatman No. 42 filter paper, and centrifugation at 5000 rpm for 15 minutes to remove solids. All bioassays were conducted using these extracts. The concentrations (0–200 g L^-1^) were selected to cover a broad range for initial screening purposes.

Seeds for the weed species (*S. arvensis* L., *A. retroflexus* L., and *A. fatua* L.) and mixed-use species (*S. cereale* L.) were collected at Moghan Research and Natural Resources Center (48°20′ E, 38°19′ N) from plants with ripening inflorescences. Following collection, inflorescences were air-dried at 25 ± 2°C for three weeks, and seeds were manually separated from the inflorescences by rubbing. *Amaranthus retroflexus*, *A. fatua*, and *S. arvensis* seeds required dormancy-breaking treatment. These seeds were treated with gibberellic acid (GA_3_) at 1500, 1000, or 600 ppm respectively, where these concentrations were previously defined to be sufficient and appropriate [37–39].

Seeds for the crop species (*T. aestivum* L. var. Cascogen and *V. faba* L. cv. Shadan/G-Faba/133) were obtained from commercial suppliers (Seed and Plant Improvement Institute of Iran, Karaj, Iran) and used without further selection. Therefore, the storage duration, moisture content at harvest, and after‑ripening status of the crop species are unknown but in accordance with standard commercial production. Upon receipt, seeds were stored at 25 ± 2°C and used within 14 days. Once seed lots were obtained, all seeds were stored under equivalent conditions where temperature, humidity, and storage time were controlled as much as was practically possible.

For the bioassays, all seeds were first sterilized with 1% sodium hypochlorite and washed at least three times with distilled water. Three triplicates of twenty seeds were randomly placed in Petri dishes (9 cm in diameter for small seeds and 15 cm in diameter for *V. faba* seeds) on No. 42 filter paper, giving a total of sixty seeds per analysis. Ten mL of extract at the different concentrations or distilled water was then added to the filter paper, ensuring that the liquid covered the filter paper completely and evenly. To ensure that each replicate dish maintained equivalent and consistent levels of humidity, the dishes were sealed in transparent plastic bags. Sealed plates were transferred to a BINDER KBW 240 germinator (Germany) set at 25°C; no additional lighting was used, and throughout the seeds were kept in the dark. A factorial design was used, with three technical replications in a completely randomized design. Seed germination was monitored daily for 14 days. Seeds with a radicle length of 1 mm were considered germinated [40]. The germination percentage (GP) and germination rate (GR) were recorded daily, from which the mean germination time (MGT), mean daily germination (Ḡ), peak value (PV), and germination value (GV) were calculated. Measurements of seedling radicle and plumule lengths were taken 14 days after incubation at 25°C to 1 mm accuracy. The germination percentage (GP), defined as the ratio of the number of germinated seeds (S) to the total number of seeds in the experimental sample (T), was calculated via Equation 1, as described by Scott et al. [41].


$$\begin{array}{c} \text{GP}=\frac{\text{S}}{\text{T}}\times \text{100} \end{array}$$ (1)

The germination rate (GR), which is the number of germinated seeds over time, is represented in Equation 2 from Maguire [42].


$$\begin{array}{c} \text{GR}=\frac{Numberofnormalseedlings}{Daystofirstcount}+\ldots \frac{Numberofnormalseedlings}{Daystofinalcount} \end{array}$$ (2)

The mean germination time (MGT) was calculated via Equation 3 described by Ellis [43].


$$\begin{array}{c} \text{MGT}=\frac{\sum (\text{nd})}{\sum \text{n}} \end{array}$$ (3)

The mean time required for germination (MGT) was calculated as the ratio of the sum of the number of days counted from the start of the experiment (d), divided by the sum (n), which represents the number of germinated seeds on day (d).

Protocols and equations are further detailed in Ahmadnia et al. [37] or elsewhere as indicated.

### Gas chromatography–mass spectrometry (GC-MS)

This study employed a comparative extraction approach to optimise metabolite detection for qualitative GC–MS analysis. As stated above, dried material of *A. repens* was pooled into a single bulk sample and finely ground to obtain a homogeneous sample that ensured consistency of plant source across experiments. No biological replication was performed; analyses represent technical measurements of the same homogenised plant material.

#### Extraction procedure.

Equal to the 100 g in 1000 ml above, 5 g of air-dried and powdered *A. repens* material was extracted with 50 mL of deionised water at 25 ± 2 °C for 24 hours using shaking. The extract was then sonicated for 15 minutes using an ultrasonic homogenizer (UP100H, Hielscher, Germany) and subsequently filtered through Whatman No. 54 filter paper under vacuum (ROCKER 811 oil-free vacuum pump, QTE Technologies, Taiwan). In order to make this aqueous extract suitable for injection into the GC-MS, it was dried in a two-step process: 1) Ambient drying at 25 ± 2 °C for 7 days to remove bulk water and 2) Freeze-drying using a VaCo5 freeze dryer (ZIBRUS Technology GmbH, Germany). This two‑step drying approach was used to concentrate aqueous extracts while minimizing thermal degradation of metabolites, following established protocols [44,45]. The resulting residue was reconstituted in 10 mL of deionised water containing 0.5% (v/v) DMSO, filtered through a 0.22 μm syringe filter (Sarstedt), and analysed immediately by GC–MS.

#### GC–MS analysis.

Samples were analysed using an Agilent 7890B gas chromatograph coupled to an Agilent 5977A mass-selective detector (Agilent Technologies Inc., Palo Alto, USA). Samples were injected at a volume of 0.1 μL using helium as the carrier gas. A multimode inlet (MMI) was operated in split mode with a split ratio of 15:1 and a total flow rate of 48 mL/min. The MMI temperature was programmed to increase from 100 °C to 380 °C at a rate of 8 °C/min. The oven temperature was initially set at 40 °C and increased to 325 °C at a rate of 4 °C/min, followed by a future increase to 400 °C at 10 °C/min and held for 12.5 min. The total run time was 46.95 min. Mass spectrometric detection was performed using electron ionisation (EI) at 70 eV with a quadrupole mass analyser. Samples were analysed using an automated liquid sampler. Data were acquired in full scan mode over a broad m/z range (approximately 10–300+), suitable for library-based compound identification.

#### Data acquisition and processing.

Data acquisition and initial processing were performed using Agilent MassHunter software. Chromatographic peak detection and integration were conducted using the ChemStation integrator (autoint1.e), with peak identification based on apex spectral extraction. Data were acquired as total ion chromatograms (TIC). Peaks within the solvent front were removed prior to analysis, and a post-processing match quality cutoff of >80% was applied to retain high-confidence identifications. Mass spectral identification was carried out by comparison against the NIST05a and Wiley 7N spectral libraries. Metabolites were tentatively identified based on mass spectral similarity to reference library entries (NIST05a and Wiley7n), corresponding to Metabolomics Standards Initiative (MSI) level 2 identification. Relative abundances were estimated based on peak areas from the total ion chromatogram and expressed as percentages of the total integrated signal. No internal standards were used, and no quantitative normalisation was applied, as the analysis was designed for qualitative metabolite profiling. Library matches were manually inspected to remove obvious artefacts and non-biogenic compounds.

### Statistical analysis

The data were analyzed using RStudio and SAS 9.4 software. Data normality was assessed using the Shapiro-Wilk test, and non-normal data were transformed using a square root transformation. Homogeneity of variances was verified using Levene’s test. After confirming these assumptions, ANOVA was performed, and mean comparisons were conducted using at LSD 5% significance. Pearson correlation was applied only to traits that met the assumptions of normality and homogeneity of variance. For traits that did not meet these assumptions, appropriate square root transformations were applied prior to correlation analysis to ensure valid results. Additionally, we used the R corrplot package in RStudio to visualize them.

## Results

### *Acroptilon repens* extract reduces seed germination and seedling parameters in a concentration-dependent manner

**Fig 1:**
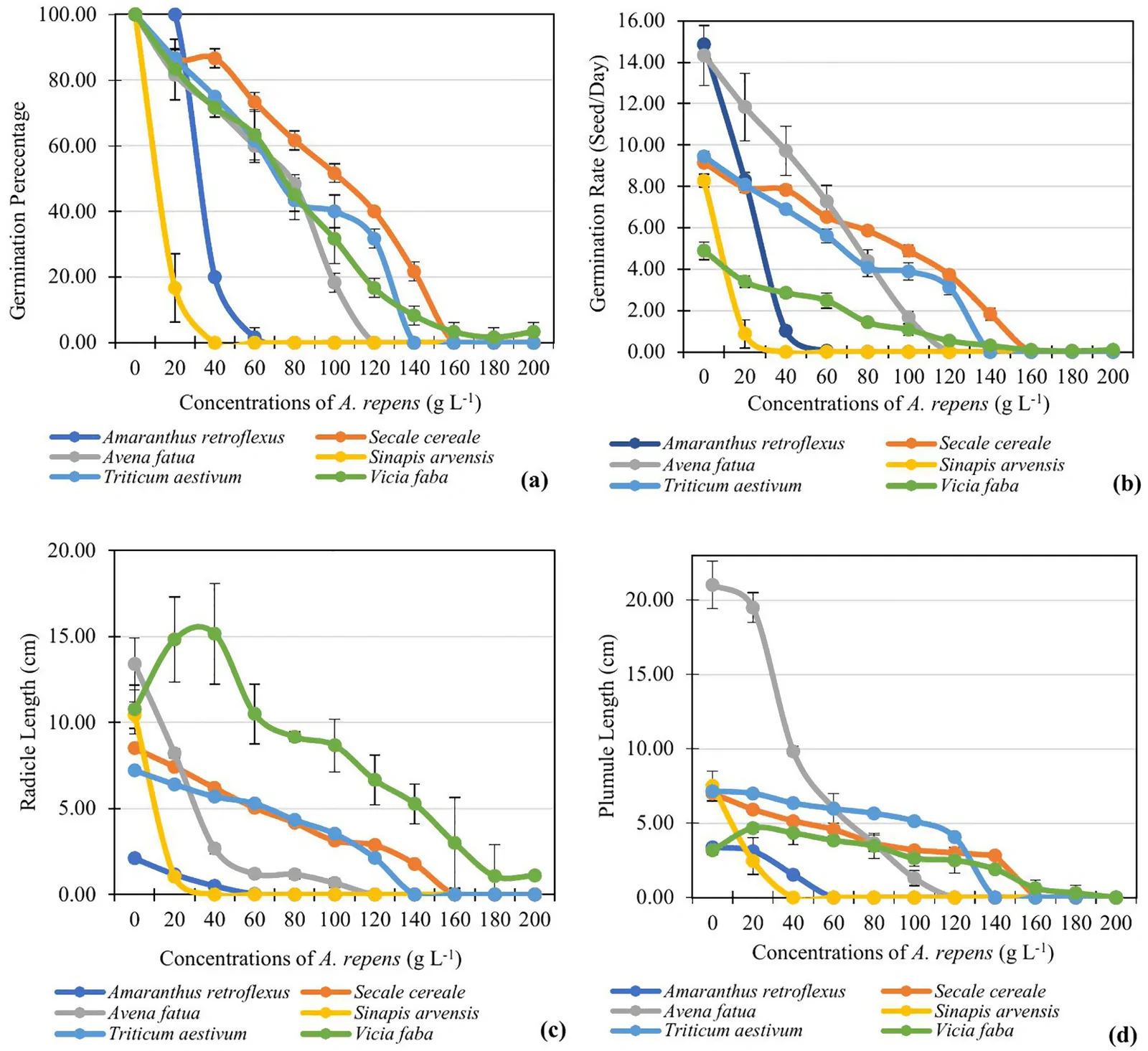
The effect of *A. repens* extract on percent germination (a), germination rate (b), radicle length (c), and plumule length (d). Data are shown as averages ± standard deviation of 20 plants observed for 14 days as described in the methods.

All germination indices declined consistently and significantly with increasing concentrations of *A. repens* extract (*P < 0.01*; Tables 1–3; Fig 1a-d), indicating including *A. repens* extract *in vitro* causes a strong concentration-dependent inhibitory effect on germination and early seedling growth. Because trends across indices were consistent, results are presented in a consolidated manner to emphasise biologically meaningful differences among species and treatment levels. Inhibition was more pronounced in weed species, which showed steeper declines across the concentration gradient and reached complete inhibition at lower concentrations than crops. *Amaranthus retroflexus* and *S. arvensis* were the most sensitive species, *T. aestivum*, *V. faba*, and *S. cereale* were less sensitive, and *A. fatua* showed an intermediate response. Germination was fully inhibited at concentrations of ≥ 40 g L^-1^ for *S. arvensis*, ≥ 80 g L^-1^ for *A. retroflexus*, ≥ 120 g L^-1^ for *A. fatua*, ≥ 140 g L^-1^ for *T. aestivum*, and ≥ 160 g L^-1^ for *S. cereale*, whereas *V. faba* maintained low-level germination even at the highest concentrations tested (Fig 1a). Consequently, data could not be collected for germination and seedling growth metrics at or above these concentrations (Fig 1b-d). Correlation analysis confirmed strong negative relationships between extract concentration and germination metrics across species (S1 Fig).

**Table 1:** Analysis of variance (mean square) of the effects of *A. repens* aqueous extract and plant species on germination and related characteristics.

Source of Variation	df	GP	GR	MGT	SPL	SRL
Concentration	10	20246.23^**^	191.84^**^	5.40^**^	143.81^**^	14.82^**^
Plant species	5	6284.44^**^	77.85^**^	12.62^**^	110.41^**^	23.16^**^
Concentration × Plant species	50	804.38^**^	13.25^**^	0.77^**^	24.03^**^	1.00^**^
Error	132	8.83	0.14	0.14	0.17	0.05
Coeff Var (%)	–	9.31	13.38	25.63	14.97	18.82

**Significant at 1% probability level.

Germination percentage, (GP); Germination rate, (GR); Mean germination time, (MGT); Seedling plumule length, (SPL); Seedling radicle length, (SRL).

**Table 2:** Effect of increasing *A. repens* extract concentration on germination and seedling parameters.

Plant	Concentration (g L^-1^)	GP (%)	GR (Seed/day)	MGT (Seed/day)	SPL (cm)	SRL (cm)
***A. retroflexus***	0	100.00 ± 0.00	14.86± 0.07	1.55 ± 0.02	3.37 ± 0.09	2.09 ± 0.26
	20	100.00 ± 0.00	8.30 ± 0.21	2.51 ± 0.07	3.12 ± 0.52	1.16 ± 0.12
	40	20.00 ± 0.00	1.00 ±0.00	4.66 ± 0.16	1.53 ± 0.06	0.50 ± 0.05
	60	1.66 ± 1.66	0.05 ± 0.05	2.00 ± 2.00	0.00 ± 0.00	0.03 ± 0.03
	80	0.00±0.00	0.00±0.00	0.00±0.00	0.00±0.00	0.00±0.00
***S. cereale***	0	100.00 ± 0.00	9.13± 0.10	2.30 ± 0.02	7.00 ± 0.01	8.50 ± 0.09
	20	86.66 ± 3.33	7.95 ± 0.10	2.31 ± 0.09	5.91 ± 0.06	7.42 ± 0.08
	40	86.66 ± 1.66	7.83 ± 0.12	2.30 ± 0.01	5.12 ± 0.09	6.19 ± 0.09
	60	73.33 ± 1.66	6.52 ± 0.03	2.44 ± 0.11	4.59 ± 0.14	5.03 ± 0.03
	80	61.66 ± 1.66	5.86 ± 0.12	2.16 ± 0.04	3.62 ± 0.06	4.16 ± 0.07
	100	51.66 ± 1.66	4.88 ± 0.14	2.16 ± 0.03	3.16 ± 0.08	3.12 ± 0.01
	120	40.00 ± 0.00	3.72 ± 0.11	2.20 ± 0.08	2.99 ± 0.008	2.87 ± 0.05
	140	21.66 ± 1.66	1.83 ± 0.16	2.46 ± 0.03	2.82 ± 0.06	1.77 ± 0.11
	160	0.00±0.00	0.00±0.00	0.00±0.00	0.00±0.00	0.00±0.00
***A. fatua***	0	100.00 ± 0.00	14.33± 0.83	1.80 ± 0.11	21.03 ±0.93	13.40 ± 0.87
	20	81.66 ± 4.40	11.83 ±0.94	1.67 ± 0.06	19.50 ± 0.57	8.20 ± 0.15
	40	71.66 ± 1.66	9.72 ± 0.68	2.05 ± 0.19	9.83 ± 0.20	2.66 ± 0.16
	60	60.00 ± 2.88	7.27 ± 0.45	1.98 ± 0.20	6.00 ± 0.57	1.20 ± 0.15
	80	48.33 ± 1.66	4.38 ± 0.30	2.31 ± 0.17	3.63 ± 0.31	1.16 ± 0.16
	100	18.33 ± 1.66	1.66 ± 1.66	2.33 ± 0.33	1.30 ± 0.30	0.66 ± 0.16
	120	0.00±0.00	0.00±0.00	0.00±0.00	0.00±0.00	0.00±0.00
***S. arvensis***	0	100.00 ± 0.00	8.27± 0.18	2.68 ± 0.04	7.50 ± 0.57	10.43 ± 0.44
	20	16.66 ± 6.00	0.86 ± 0.39	4.86 ± 0.94	2.46 ± 0.53	1.03 ± 0.03
	40	0.00±0.00	0.00±0.00	0.00±0.00	0.00±0.00	0.00±0.00
***T. aestivum***	0	100.00 ± 0.00	9.44± 0.14	2.18 ± 0.06	7.15 ± 0.02	7.21 ± 0.11
	20	86.66 ± 1.66	8.08 ± 0.20	2.21 ± 0.02	7.00 ± 0.01	6.40 ± 0.07
	40	75.00 ± 0.00	6.88 ± 0.05	2.24 ± 0.02	6.34 ± 0.02	5.69 ± 0.08
	60	61.66 ± 1.66	5.61 ± 0.19	2.29 ± 0.03	5.97 ± 0.04	5.28 ± 0.02
	80	43.33 ± 3.33	4.08 ± 0.25	2.18 ± 0.05	5.65 ± 0.11	4.33 ± 0.05
	100	40.00 ± 2.88	3.88 ± 0.24	2.07 ± 0.03	5.13 ± 0.06	3.52 ± 0.07
	120	31.66 ±1.66	3.11 ± 0.20	2.05 ± 0.05	4.06 ± 0.034	2.12 ± 0.01
	140	0.00±0.00	0.00±0.00	0.00±0.00	0.00±0.00	0.00±0.00
***V. faba***	0	100.00 ± 0.00	4.88 ± 0.25	4.23 ± 0.24	3.16 ± 0.16	10.76 ± 0.81
	20	83.33 ± 1.66	3.39 ± 0.15	4.99 ± 0.30	4.66 ± 0.16	14.83 ± 1.42
	40	71.66 ± 1.66	2.85 ± 0.12	5.11 ± 0.16	4.33 ± 0.44	15.16 ±1.69
	60	63.33 ± 4.40	2.47 ± 0.20	5.24 ± 0.15	3.83 ± 0.16	10.50 ± 1.00
	80	45.00 ± 2.88	1.43 ± 0.12	6.41 ± 0.10	3.46 ± 0.48	9.16 ± 0.16
	100	31.33 ± 4.40	1.07 ± 0.15	6.01 ± 0.10	2.63 ± 0.31	8.66 ± 0.88
	120	16.66 ± 1.66	0.53 ± 0.04	6.27 ± 0.14	2.50 ± 0.50	6.66 ± 0.83
	140	8.33± 1.66	0.30 ± 0.05	5.50 ± 0.28	1.90 ± 0.05	5.26 ± 0.66
	160	3.33± 1.66	0.09 ± 0.04	4.66 ± 2.33	0.63 ± 0.31	3.00 ± 1.52
	180	1.66± 1.66	0.04± 0.04	2.33 ± 2.33	0.30 ± 0.30	1.06 ± 1.06
	200	3.33± 1.66	0.09 ± 0.04	4.66 ± 2.33	0.00	1.10 ± 0.05
	LSD (*P*<0.05)	4.80	0.61	1.61	0.67	1.25

Germination Percentage, (GP); Germination Rate, (GR); Mean Germination Time, (MGT); Seedling Plumule Length, (SPL); Seedling Radicle Length, (SRL). Note: Even though all concentrations were tested against all species, where the extract concentration led to complete inhibition of germination, the data are not shown.

**Table 3:** Linear slope and Pearson’s R^2^ values calculated from nonzero data points of germination and seedling parameters.

Value-Slope	*S. arvensis*	*A. retroflexus*	*A. fatua*	*V. faba*	*T. aestivum*	*S. cereale*
Germination Percentage	−0.24	−0.46	−1.27	−1.78	−1.64	−1.81
Germination Rate	−2.66	−3.59	−7.60	−37.94	−17.74	−19.79
Mean Germination Time	5.25	2.54	54.60	−4.09	−195.38	−6.95
Plumule length	−3.62	−14.77	−4.32	−34.82	−38.82	−31.08
Radicle length	−2.11	−26.50	−6.40	−11.44	−24.40	−20.64
**Value-R** ^**2**^	***S. arvensis***	***A. retroflexus***	***A. fatua***	***V. faba***	***T. aestivum***	***S. cereale***
Germination Percentage	0.98	0.87	0.95	0.93	0.97	0.97
Germination Rate	0.99	0.93	0.96	0.88	0.96	0.96
Mean Germination Time	0.57	0.04	0.35	0.02	0.25	0.00
Plumule length	0.91	0.86	0.93	0.78	0.95	0.94
Radicle length	0.99	0.91	0.79	0.79	0.97	0.98

Our data suggested that the weedy species have different responses to the crops. For instance, under control conditions, *A. retroflexus* and *A. fatua* presented the highest germination rate (14.86 ± 0.07 and 14.33 ± 0.83 seed/day) (Table 2), but these responses diverged in the presence of *A. repens* extract; *A. retroflexus* declined rapidly even at low concentrations, whereas *A. fatua* showed a more gradual reduction (Fig 1b). *Sinapis arvensis* was highly sensitive to *A. repens* extract, leading to a significant decrease in germination rates even at low concentration, whereas the germination rates of *S. cereale*, and *T. aestivum* were similar across various concentrations of *A. repens* extract and both decreased gradually (Table 2 and Fig 1b).

Mean germination time was only weakly affected by treatment, with no statistically significant changes detected; this is reflected in a very low analysis of variance effect of experimental treatments on the mean germination time (Tables 1 and 2). Although descriptive increases in mean germination time were observed at 40 g L^-1^ for *A. retroflexus* and at 20 g L^-1^ for *S. arvensis*, these changes were not statistically significant and remained within the range of natural variability relative to the control.

Application of *A. repens* extract also reduced the mean daily germination, peak value, and G value in a concentration-dependent and species-specific manner. Detailed results, including species-specific responses and statistical analyses, are provided in the Tables in S1–S3 Tables.

Seedling growth responses mirrored those observed for germination. Although the untreated plumule and radicle lengths varied greatly between the species, each parameter was reduced in response to the *A. repens* extract. Reductions were roughly linear for either plumule or radicle lengths (Table 3) and, as for germination, weed species exhibited stronger responses than crop species. Both parameters consistently declined in a concentration-dependent manner (*P* < *0.01*, Table 1) with increasing concentrations of *A. repens* extract (Table 2, Fig 1c-d). These results indicated that both germination and early growth metrics consistently distinguish between more sensitive weed species and more tolerant crops under the experimental conditions.

### Untargeted GC-MS results

Untargeted GC–MS analysis was conducted to provide a qualitative overview of compound classes present in aqueous extracts of *A. repens* and to place the observed phytotoxic effects within the context of existing chemical literature for this species. GC–MS preferentially detects volatile and semi-volatile constituents and does not capture the full spectrum of polar compounds present in aqueous extracts. Accordingly, compound identification was putative and based on library matching, and reported peak areas reflect relative signal intensities rather than quantitative concentrations. The GC–MS data therefore do not permit direct attribution of the observed biological effects to specific compounds. Instead, the detected metabolites, encompassing phenolic compounds, lipophilic allelochemicals, and nitrogen-containing derivatives (Tables 4 and Table in S4 Table) are consistent with chemical classes previously reported for *A. repens* using complementary analytical approaches. Taken together, these results provide qualitative, hypothesis-generating context for the phytotoxic effects observed in vitro, while highlighting the need for targeted chemical analyses and bioactivity-guided fractionation to establish mechanistic links between individual compounds and biological activity.

**Table 4:** Untargeted GC-MS results for *A. repens.*

Category	Area %	RT	NIST	m/z	CAS Number	Molecular Formula	Name GC	Name
Phenolic Compounds	5.4%	16.466	95	109.1	000090-05-1	C_7_H_8_O_2_	Phenol, 2-methoxy	Guaiacol (2-methoxyphenol)
	3.2%	17.382	89	110.1	000120-80-9	C_6_H_6_O_2_	1,2-Benzenediol	Catechol (1,2-benzenediol)
	8.9%	15.689	81	94.1	000108-95-2	C_6_H_6_O	Phenol	Carbolic acid
Terpenoids (sesquiterpenes)	6.7%	38.438	81	71.1	001139-30-6	C_15_H_24_O	Caryophyllene oxide	Caryophyllene oxide
Fatty acids (long-chain)	3.3%	33.183	98	71.2	000057-11-4	C_18_H_36_O_2_	Octadecanoic acid	Stearic acid
	7.3%	30.092	93	73.1	000057-10-3	C_16_H_32_O_2_	n-Hexadecanoic acid	Palmitic acid
Sterols	4.0%	31.073	90	73.1	007494-34-0	C_26_H_44_O	26-Nor-5-cholesten-3.beta.-ol-25	Sterol derivative
	61.3%	31.653	98	386.6	000057-88-5	C_27_H_46_O	Cholest-5-en-3-ol (3.beta.)-	Cholesterol

The chemical profile is dominated by sterol and lipid-derived compounds, alongside a smaller fraction of phenolic and terpenoid secondary metabolites (Table 4). The largest chromatographic contribution was attributed to cholesterol, indicating a substantial presence of the membrane in the extract. Long-chain fatty acids, including palmitic acid and stearic acid, were also detected, consistent with structural lipid components derived from plant tissue.

In addition, several low-molecular-weight phenolic compounds were identified, including phenol, catechol (1,2-benzenediol), and guaiacol (2-methoxyphenol). These compounds are commonly associated with lignin degradation and plant secondary metabolism. A sesquiterpene oxide, caryophyllene oxide, was also detected, representing the terpenoid fraction of the extract. The presence of phenolic compounds and terpenoid derivatives highlights a smaller but potentially more biologically active fraction, which may be relevant to plant–plant chemical interactions.

## Discussion

The scientific literature has increasingly demonstrated that extracts from allelopathic plants are promising alternatives to conventional herbicides for weed management in sustainable agriculture [46,47]. Since seed germination initiates all subsequent plant development and productivity, our attention was focused on how germination and early seedling development parameters were altered by *A. repens* extract. Our data showed that increasing concentrations of the *A. repens* aqueous extract significantly reduced various germination parameters, including the percentage, rate, peak value, and G value, as well as seedling parameters such as the plumule and radicle lengths of all species. Although similar inhibitory effects of aqueous extracts from allelopathic plants, including *A. repens*, have been reported previously [48–50], the key contribution of this study lies in the side-by-side comparison of weed and crop species, which revealed marked differences in sensitivity. Germination was fully inhibited at lower extract concentrations for weedy species (*S. arvensis* ≥ 40 g L^-1^, *A. retroflexus* ≥ 80 g L^-1^, *A. fatua* ≥ 120 g L^-1^) than for crops (*T. aestivum* ≥ 140 g L^-1^, *S. cereale* ≥ 160 g L^-1^), and this distinction was particularly evident in seedling growth responses. Among the weeds, *A. retroflexus* and *S. arvensis* were the most sensitive, showing strong inverse relationships between extract concentration and germination or growth, whereas cultivated species such as *V. faba* and *T. aestivum* exhibited a more gradual decline and tolerated higher concentrations. Although all treatments were prepared from a single homogenized batch of plant material, extraction efficiency may still vary across mass-to-volume ratios, representing a methodological consideration for future standardization. Overall, by systematically comparing germination and seedling responses of weed and crop species under controlled conditions, this study demonstrates a clear and quantitative differential response to *A. repens* aqueous extract that is consistent with, but more explicitly resolved than, previous reports.

Maternal effects, alongside seed maturity, age, and storage conditions, represent an important source of biological variation in germination assays. Although all species exhibited high germination levels in the absence of *A. repens* extract (Table 2, Fig 1), baseline physiological differences may have influenced sensitivity to the extract. In this study, seeds of the weedy species were freshly collected from field-grown plants, after‑ripened, and treated to break dormancy as required, whereas the crop species were obtained from Seed and Plant Improvement Institute of Iran, Karaj, Iran (as detailed in the Materials and Methods), which may differ in age, storage history, and vigour. These factors could contribute to the observed differences between weedy and cultivated species, and future studies should aim to standardize seed provenance and handling to better isolate species-specific responses from physiological variation.

Because the chemical analyses were qualitative and compound identification was putative (Tables 4 and S4 Table), the following is intended to contextualize the detected metabolite classes within the existing literature rather than to establish causal links with the observed biological effects. Relative Area (%) values reflect detector response rather than absolute concentrations, as no compound-specific calibration was performed. Consequently, while the GC–MS profiles provide useful chemical context, targeted analytical approaches and bioactivity-guided fractionation will be required to identify which compounds, if any, directly contribute to the phytotoxic effects observed.

Previous studies of *A. repens* have identified classes of secondary metabolites associated with phytotoxic effects, although their roles remain context-dependent and are not fully resolved. Polyacetylenes have been most consistently implicated: early work identified multiple root-derived polyacetylenes with inhibitory activity against germination and seedling growth of several species, and subsequent bioassay-guided fractionation confirmed that most isolated compounds exhibited phytotoxicity toward *Arabidopsis* seedlings [30,31]. Sesquiterpene lactones, including acroptilin and repin, have also been reported, although their effects appear dose-dependent, with stimulation of root elongation at low concentrations and inhibition at higher concentrations [51]; these data highlight the importance of concentration in determining biological outcome. In contrast, earlier claims regarding specific flavones such as 7,8-benzoflavone have not been substantiated, as later analyses failed to detect this compound in plant tissues or soils and the original report was subsequently retracted [30]. At the whole-extract level, aqueous extracts and powdered root material have been shown in greenhouse and laboratory studies to suppress germination and seedling growth [52], supporting a general phytotoxic effect, but without resolving the responsible compounds.

In the context of these studies, our findings are consistent with previously reported inhibitory effects of *A. repens* extracts, but extend this literature by quantitatively comparing responses across weed and crop species under controlled conditions. Importantly, the extracts analysed by GC–MS in the present study were prepared using a different protocol from that used in the biological assays, and compound identification is putative and qualitative. Therefore, the detected metabolites should be interpreted as providing chemical context rather than direct evidence linking specific compounds to the observed biological activity. Taken together with earlier work, the results support a model in which multiple classes of metabolites may contribute to phytotoxic effects *in vitro*, while the ecological relevance, mode of action, and compound-specific contributions remain to be established through targeted and soil-based studies.

Several phenolic compounds, including phenol, guaiacol (2-methoxyphenol), catechol (1,2-benzenediol), and carbolic acid were putatively detected in *A. repens* extracts. Phenolic compound have been reported to exhibit phytotoxic effects in other plant systems, including inhibiting seed germination [53–59] and, more specifically, weed seed germination [54]. Phenolics are known to induce oxidative stress, disrupt membrane integrity, and interfere with enzymatic processes associated with reserve mobilization during germination, and may interfere with respiratory enzyme activity [55–59].

The terpenoid, caryophyllene oxide, was identified (Table 4). This sesquiterpene is commonly reported in plant essential oil [60]. Terpenoid compounds have been associated with phytotoxic activity in a range of plant systems, often acting through effects on membrane integrity, respiration, or signalling pathways [60]. Caryophyllene oxide has been reported as a bioactive constituent in extracts and essential oils of *Euphorbiaceae Croton doctoris* S. Moore where it was demonstrated to have inhibitory effects on seed germination and seedling growth [61], although such effects are typically observed in complex mixtures rather than attributable to a single compound.

Long-chain fatty acids, including palmitic acid and stearic acid, were also detected (Table 4). These compounds are common components of plant lipid metabolism and cellular structure and are frequently observed in GC–MS profiles of plant extracts. While fatty acids have occasionally been reported to influence seedling growth and membrane function, their role in allelopathic interactions is generally considered secondary and concentration-dependent. Similarly, the extract was dominated by sterol compounds, primarily cholest-5-en-3-ol (cholesterol), along with a sterol derivative (Table 4). These compounds are key structural components of cellular membranes and are likely derived from bulk plant tissue rather than representing specialised secondary metabolites. Although sterols can influence membrane properties, they are not typically considered primary drivers of allelopathic activity. Accordingly, their high relative abundance in the extract is interpreted as reflecting the underlying biochemical composition of the tissue rather than a direct contribution to the observed phytotoxic effects.

## Supporting information

S1 Table Analysis of variance (mean square) of the effects of *A. repens* aqueous extract and plant species on germination and related characteristics. **Significant at the 1% probability level. Mean daily germination, (Ḡ); Peak value, (PV); G value, (G).
(TIF)

S2 Table Interaction effect of concentration and plant species on germination and related characteristics. Mean daily germination, (Ḡ); Peak value, (PV); G value, (G).
(TIF)

S3 Table Linear slope and Pearson’s R^2^ values calculated from nonzero data points of germination and seedling parameters. (TIF)

S4 Table Untargeted GC-MS results of *A. repens.* (TIF)

S1 Fig Pearson’s correlation coefficients (n = 198) among five quantitative traits on *A. retroflexus*, *S. cereale*, *A. fatua*, *S. arvensis*, *T. aestivum*, and *V. faba.* The correlation coefficients were significant at the statistical probability level of 1 and 5%. GP, germination percentage; GR, germination rate; MGT, Mean germination time; SPL, Seedling plumule length; SRL, Seedling radicle length.
(TIF)
