# Supplementary material for: Under controlled conditions, lower concentrations of aqueous extracts of Acroptilon repens are needed to inhibit seed germination and seedling growth of weeds than crops
Source: PLoS One. 2026 Aug 27;21(8):e0354454. doi: 10.1371/journal.pone.0354454 (PMC13521396; doi:10.1371/journal.pone.0354454)
Supplement: Supplementary file 1 [file pone.0354454.s009.pdf]

PLOS ONE

Aqueous extract of allelopathic Acroptilon repens (Russian knapweed) has the potential to be used as a bioherbicide for managing noxious weeds

--Manuscript Draft--

**Manuscript Number:**	PONE-D-25-29786
**Article Type:**	Research Article
**Full Title:**	Aqueous extract of allelopathic Acroptilon repens (Russian knapweed) has the potential to be used as a bioherbicide for managing noxious weeds
**Short Title:**	Allelopathic properties of Acroptilon repens
**Corresponding Author:**	Mohammad Taghi Alebrahim<br>University of Mohaghegh Ardabili<br>Ardabil, IRAN, ISLAMIC REPUBLIC OF
**Keywords:**	Acroptilon repens, Allelopathy, Bioherbicides, Germination inhibition
**Abstract:**	Preventing weeds from hindering agricultural production often relies on chemical inputs, which can increase costs, harm the environment, and be ineffective. Natural products, particularly allelopathic plant extracts, are being explored as potential sources for developing eco-friendly bioherbicides to minimize chemical herbicide dependency. This study assessed the herbicidal effects of aqueous extract from an allelopathic weed, Acroptilon repens (Russian knapweed). We assessed its impact on seed germination and early growth of three weedy species (Sinapis arvensis, Amaranthus retroflexus, and Avena fatua) and three crop species (Secale cereale, Triticum aestivum, and Vicia faba). The extract caused a significant ($P \leq 0.01$), linear, and concentration-dependent decline in several germination metrics (germination percentage, rate, index, mean daily germination, peak value, G value) and seedling growth (plumule and radicle length). In contrast, mean germination time (MGT) remained statistically unchanged ($P > 0.05$) across all concentrations tested. Pearson's correlation analysis revealed strong positive associations between germination percentage and the other variables. Germination was fully inhibited for Sinapis arvensis $\geq 40 gL^{-1}$, Amaranthus retroflexus $\geq 80 gL^{-1}$, Avena fatua $\geq 120 gL^{-1}$, Triticum aestivum $\geq 140 gL^{-1}$, Secale cereale $\geq 160 gL^{-1}$, and Vicia faba $\geq 200 gL^{-1}$ of Russian knapweed extract; suggesting the more invasive a species is, the more sensitive it is to Russian knapweed extract. To explore potential mechanisms, we used untargeted GC-MS profiling and identified several compounds with reported allelopathic activity, including carvacrol, phenol, benzenediols, anisaldehyde, and organic acids such as acetic and propanoic acid. These findings suggest that phytotoxic secondary metabolites in A. repens may underlie its inhibitory effects. Although further research is required to isolate and confirm the bioactivity of individual components, our findings provide preliminary evidence for the potential application of A. repens aqueous extract as a natural, broad-spectrum bioherbicide. However, comprehensive ecological and agronomic assessments are essential prior to field application.
**Order of Authors:**	Fatemeh Ahmadnia, Ph.D.
	Elham Samadi-Kalkhoran
	Mohammad Taghi Alebrahim
	Dana R. MacGregor
**Additional Information:**	
**Question**	**Response**
**Financial Disclosure**<br><br>Enter a financial disclosure statement that describes the sources of funding for the work included in this submission. Review the submission guidelines for detailed	Yes

Powered by Editorial Manager® and ProduXion Manager® from Aries Systems Corporation

requirements. View published research articles from *PLOS ONE* for specific examples.<br><br>This statement is required for submission and **will appear in the published article** if the submission is accepted. Please make sure it is accurate.<br><br>**Funded studies**<br>Enter a statement with the following details:<br>• Initials of the authors who received each award<br>• Grant numbers awarded to each author<br>• The full name of each funder<br>• URL of each funder website<br>• Did the sponsors or funders play any role in the study design, data collection and analysis, decision to publish, or preparation of the manuscript?<br><br>Did you receive funding for this work?	
Please add funding details.<br>as follow-up to "**Financial Disclosure**<br><br>Enter a financial disclosure statement that describes the sources of funding for the work included in this submission. Review the submission guidelines for detailed requirements. View published research articles from *PLOS ONE* for specific examples.<br><br>This statement is required for submission and **will appear in the published article** if the submission is accepted. Please make sure it is accurate.	Rothamsted Research receives strategic funding from the Biotechnology and Biological Sciences Research Council of the United Kingdom (BBSRC). We acknowledge support from the Growing Health Institute Strategic Programme [BB/X010953/1; BBS/E/RH/230003A] for DRM.

Powered by Editorial Manager® and ProduXion Manager® from Aries Systems Corporation

**Funded studies**<br>Enter a statement with the following details:<br>• Initials of the authors who received each award<br>• Grant numbers awarded to each author<br>• The full name of each funder<br>• URL of each funder website<br>• Did the sponsors or funders play any role in the study design, data collection and analysis, decision to publish, or preparation of the manuscript?<br><br>Did you receive funding for this work?"	
Please select the country of your main research funder (please select carefully as in some cases this is used in fee calculation).<br>as follow-up to "**Financial Disclosure**<br><br>Enter a financial disclosure statement that describes the sources of funding for the work included in this submission. Review the submission guidelines for detailed requirements. View published research articles from *PLOS ONE* for specific examples.<br><br>This statement is required for submission and **will appear in the published article** if the submission is accepted. Please make sure it is accurate.<br><br>**Funded studies**<br>Enter a statement with the following details:<br>• Initials of the authors who received each award<br>• Grant numbers awarded to each author<br>• The full name of each funder<br>• URL of each funder website<br>• Did the sponsors or funders play any role in the study design, data collection and analysis, decision to publish, or preparation of the manuscript?<br><br>Did you receive funding for this work?"	UNITED KINGDOM - GB
**Competing Interests**	NO authors have competing interests

Use the instructions below to enter a competing interest statement for this submission. On behalf of all authors, disclose any competing interests that could be perceived to bias this work—acknowledging all financial support and any other relevant financial or non-financial competing interests.<br><br>This statement is required for submission and **will appear in the published article** if the submission is accepted. Please make sure it is accurate and that any funding sources listed in your Funding Information later in the submission form are also declared in your Financial Disclosure statement.<br><br>View published research articles from *PLOS ONE* for specific examples.<br><br>**NO authors have competing interests**<br><br>Enter: *The authors have declared that no competing interests exist.*<br><br>**Authors with competing interests**<br><br>Enter competing interest details beginning with this statement:<br><br>*I have read the journal's policy and the authors of this manuscript have the following competing interests: [insert competing interests here]*<br><br>* typeset	
**Ethics Statement**<br><br>Enter an ethics statement for this submission. This statement is required if the study involved:<br><br>• Human participants<br>• Human specimens or tissue<br>• Vertebrate animals or cephalopods<br>• Vertebrate embryos or tissues	N/A

• Field research<br><br>Write "N/A" if the submission does not require an ethics statement.<br><br>General guidance is provided below. Consult the submission guidelines for detailed instructions. **Make sure that all information entered here is included in the Methods section of the manuscript.**	

Powered by Editorial Manager® and ProduXion Manager® from Aries Systems Corporation

**Format for specific study types**<br><br>**Human Subject Research (involving human participants and/or tissue)**<br>• Give the name of the institutional review board or ethics committee that approved the study<br>• Include the approval number and/or a statement indicating approval of this research<br>• Indicate the form of consent obtained (written/oral) or the reason that consent was not obtained (e.g. the data were analyzed anonymously)<br><br>**Animal Research (involving vertebrate animals, embryos or tissues)**<br>• Provide the name of the Institutional Animal Care and Use Committee (IACUC) or other relevant ethics board that reviewed the study protocol, and indicate whether they approved this research or granted a formal waiver of ethical approval<br>• Include an approval number if one was obtained<br>• If the study involved *non-human primates*, add *additional details* about animal welfare and steps taken to ameliorate suffering<br>• If anesthesia, euthanasia, or any kind of animal sacrifice is part of the study, include briefly which substances and/or methods were applied<br><br>**Field Research**<br><br>Include the following details if this study involves the collection of plant, animal, or other materials from a natural setting:<br>• Field permit number<br>• Name of the institution or relevant body that granted permission	
**Data Availability**<br><br>Authors are required to make all data underlying the findings described fully available, without restriction, and from the time of publication. PLOS allows rare exceptions to address legal and ethical concerns. See the PLOS Data Policy and FAQ for detailed information.	Yes - all data are fully available without restriction

A Data Availability Statement describing where the data can be found is required at submission. Your answers to this question constitute the Data Availability Statement and **will be published in the article**, if accepted.<br><br>**Important:** Stating 'data available on request from the author' is not sufficient. If your data are only available upon request, select 'No' for the first question and explain your exceptional situation in the text box.<br><br>Do the authors confirm that all data underlying the findings described in their manuscript are fully available without restriction?	
**Describe where the data may be found in full sentences. If you are copying our sample text, replace any instances of XXX with the appropriate details.**<br><br>• If the data are **held or will be held in a public repository**, include URLs, accession numbers or DOIs. If this information will only be available after acceptance, indicate this by ticking the box below. For example: *All XXX files are available from the XXX database (accession number(s) XXX, XXX.).*<br>• If the data are all contained **within the manuscript and/or Supporting Information files**, enter the following: *All relevant data are within the manuscript and its Supporting Information files.*<br>• If neither of these applies but you are able to provide **details of access elsewhere**, with or without limitations, please do so. For example:<br><br>*Data cannot be shared publicly because of [XXX]. Data are available from the XXX Institutional Data Access / Ethics Committee (contact via XXX) for researchers who meet the criteria for access to confidential data.*<br><br>*The data underlying the results presented in the study are available from (include the name of the third party*	Information will only be available after acceptance

Powered by Editorial Manager® and ProduXion Manager® from Aries Systems Corporation

and contact information or URL).<br>• This text is appropriate if the data are owned by a third party and authors do not have permission to share the data.<br><br>* typeset	
Additional data availability information:	Tick here if the URLs/accession numbers/DOIs will be available only after acceptance of the manuscript for publication so that we can ensure their inclusion before publication.

Powered by Editorial Manager® and ProduXion Manager® from Aries Systems Corporation

Manuscript
Click here to access/download;Manuscript;plos one manuscript 14040310.docx

Aqueous extract of allelopathic *Acroptilon repens* (Russian knapweed) has the potential to be used as a bioherbicide for managing noxious weeds

Short title: Allelopathic properties of *Acroptilon repens*

Fatemeh Ahmadnia[1¶], Elham Samadi-Kalkhoran[1¶], Mohammad Taghi Alebrahim[1*¶], Dana R. MacGregor[2¶]

[1] Department of Plant Production and Genetics, Faculty of Agricultural Sciences and Technology, University of Mohaghegh Ardabili, Ardabil, Iran

[2] Rothamsted Research, Protecting Crops and the Environment, West Common, Harpenden, Hertfordshire, AL5 2QJ, UK

* Corresponding author

Email: m_ebrahim@uma.ac.ir (M.T.A.)

¶These authors contributed equally to this work.

Abstract

Preventing weeds from hindering agricultural production often relies on chemical inputs, which can increase costs, harm the environment, and be ineffective. Natural products, particularly allelopathic plant extracts, are being explored as potential sources for developing eco-friendly bioherbicides to minimize chemical herbicide dependency. This study assessed the herbicidal effects of aqueous extract from an allelopathic weed, *Acroptilon repens* (Russian knapweed). We assessed its impact on seed germination and early growth of three weedy species (*Sinapis arvensis*, *Amaranthus retroflexus*, and *Avena fatua*) and three crop species (*Secale cereale*, *Triticum aestivum*, and *Vicia faba*). The extract caused a significant ($P \leq 0.01$), linear, and concentration-dependent decline in several germination metrics (germination percentage, rate, index, mean daily germination, peak value, G value) and seedling growth (plumule and radicle length). In contrast, mean germination time (MGT) remained statistically unchanged ($P > 0.05$) across all concentrations tested. Pearson's correlation analysis revealed strong positive associations between germination percentage and the other variables. Germination was fully inhibited for *Sinapis arvensis* ≥ 40 g L^{-1}, *Amaranthus retroflexus* ≥ 80 g L^{-1}, *Avena fatua* ≥ 120 g L^{-1}, *Triticum aestivum* ≥ 140 g L^{-1}, *Secale cereale* ≥ 160 g L^{-1}, and *Vicia faba* ≥ 200 g L^{-1} of Russian knapweed extract; suggesting the more invasive a species is, the more sensitive it is to Russian knapweed extract. To explore potential mechanisms, we used untargeted GC-MS profiling and identified several compounds with reported allelopathic activity, including carvacrol, phenol, benzenediols, anisaldehyde, and organic acids such as acetic and propanoic acid. These findings suggest that phytotoxic secondary metabolites in *A. repens* may underlie its inhibitory effects. Although further research is required to isolate and confirm the bioactivity of individual components, our findings provide preliminary evidence for the potential application of *A. repens* aqueous extract as a natural, broad-spectrum bioherbicide. However, comprehensive ecological and agronomic assessments are essential prior to field application.

Introduction

Traditional management approaches frequently depend on chemical inputs, particularly herbicides, to mitigate the adverse impacts of weeds on agricultural output [1]. The overuse and indiscriminate application of these substances, however, results in herbicide resistance [2], significant environmental issues, such as the degradation of agricultural ecosystems, accumulation of

chemical residues, and the contamination of vital resources like air, water, and soil [3,4]. Therefore, in line with environmental preservation and economic sustainability measures, less harmful but equally effective alternatives are needed for successful weed management strategies in agriculture production [5].

A promising eco-friendly method for controlling weeds is the use of allelopathic plants, which are presently being investigated as a substitute for synthetic pesticides [6,7]. The International Allelopathy Society (IAS) has extended its definition of allelopathy to include any processes involving secondary metabolites generated by plants, microorganisms, viruses, and fungi that impact the growth and development of agricultural and biological systems [8]. Thus, allelopathy is related to the inhibitory or promoting effects of biological molecules or secondary metabolites from one species on another, resulting in interactions between plants, soil, and pests, such as insects, fungi, or microbes [9,10,6,8].

Allelopathic compounds, which are non-nutritive, can be presented in various parts of the plant. They may be released into the environment through leachate, root secretions, and volatile or decomposing materials [11,12]. These allelochemicals have the potential to influence several physiological processes in plants, including photosynthesis, respiration, cell division, pigment and protein synthesis, hormone production, membrane permeability, nitrogen fixation, pollen germination, and the enzymatic activities essential for plant growth and development [13]. However, allelochemicals disrupt essential biological functions, including cell division, hormone biosynthesis, mineral absorption, and transport [14]. They also influence membrane permeability [15], stomatal fluctuations, and the water relations of plants, leading to a considerable reduction in growth [16,17]. The impacts of these disruptions include heightened root necrosis, decreased germination, twisting of root axes, discoloration, diminished weight gain, and reduced reproductive potential [18]. Plant-to-plant allelopathic effects or ‘phytoallelopathy’ have been reported across a wide range of interactions relevant within agricultural ecosystems, including between crop-crop, crop-weed, weeds-weeds, and weeds-trees [19].

Russian knapweed has a several homotypic scientific names (*Acroptilon repens, Rhaponticum repens*, *Centaurea repens*, and *Leuzea repens*); we will use *Acroptilon repens.* This herbaceous perennial plant belongs to the *Asteraceae* family, has allelopathic properties [20,21,22,23,24,25,26]. The literature demonstrates Russian knapweed extract reduces germination and seedling growth indicators of various weeds including Bladder campion (*Silene*

vulgaris), Cleavers (*Galium aparine*), Common purslane (*Portulaca oleracea*), Common Yellow marrow (*Abutilon theophrasti*), Redroot pigweed (*Amaranthus retroflexus*), Lamb's quarters (*Chenopodium album*), Velvetleaf (*Abutilon theophrasti*) as well as crop species including barley (*Hordeum vulgare* 'Vantage'), tomato (*Solanum lycopersicum* 'Firestell') and wheat (*Triticum aestivum*) [20,27,28,29,24,3]. Researchers like Nabati Souha et al., [24] found that at concentrations greater than 10%, Russian knapweed extract altered the germination indicators including percentage, rate, plumule length, radicle length, and the fresh weight of the seedlings and seedling dry weight of *Sinapis arvensis*. Alebrahim et al. [23] measured a 77% reduction in wheat weight, and Hatami hampa et al. [29] observed a 47.62% decrease in wheat dry weight at a 20% concentration of Russian knapweed extract. Russian knapweed extract has also been used in the treatment of epilepsy, inhibiting damage to the mucosa, and managing blood pressure [30,31,32].

Native to Eurasia, Russian knapweed is a highly competitive plant that can grow into large monotypic stands. This noxious weed can therefore be a serious crop pest across its native and introduced ranges. In Iran, its introduced range includes the northwestern provinces of Azerbayjan, Zanjan, and Tehran [33]. When Russian knapweed is present in wheat and corn fields, it dramatically reduces both yield and flour quality. Even small amounts of cross-contamination from Russian knapweed seeds into the wheat cause the flour to taste bitter [34,35]. Russian knapweed's biochemical defense system involves producing repin, acroptilin, and hyrcanin [36], sesquiterpene lactones that are highly toxic neurotoxins [37,38] and a sesquiterpenoid alkaloid that had potent cytotoxic activities against a tumor cell line [39].

Herein we evaluate how the aqueous extract of Russian knapweed (*Acroptilon repens* L.) influences germination and growth indicators of redroot pigweed (*Amaranthus retroflexus*), wild oats (*Avena fatua*), wild mustard (*Sinapis arvensis*), wheat (*Triticum aestivum* L.), fava bean (*Vicia faba*) and cereal rye (*Secale cereale* L.). *Amaranthus retroflexus*, *Avena fatua,* and *Sinapis arvensis* are all widespread and problematic weeds in many different cropping systems, including in *Triticum aestivum* [40] and *Avena fatua* is a problem in *Vicia faba* [40]. *Avena fatua* and its cultivated relative *Avena sativa* (edible oats) are estimated to have diverged between 0.4-2.3 million years ago[41] and while the latter is an important crop worldwide, the former is a problematic weed that has allelopathic properties of its own [42]. Although *Secale cereale* L. is reported as an herbicide-resistant weed in *Triticum aestivum* [40] and it is reported to be a

problematic, introduced weed in the inland western and midwestern parts of the United States [43, 44]. it is also an important cover crop that is used to improve soil health and suppress weeds [45,46,47,48] through its allelopathic activity [49]. Therefore, our data on the efficacy of Russian Knapweed extract on these weedy species compared to cultivated species will not only identify what concentrations are required to cause an effect and what growth indicators are affected, but they contextualize what potential Russian knapweed extract has as a field-relevant bioherbicide.

Materials and methods

Collection and storage of seeds

This research is designed to assess the impact of Russian knapweed (*Acroptilon repens* L.) extract on germination of various problematic weed species, including redroot pigweed (*Amaranthus retroflexus*) and wild mustard (*Sinapis arvensis*), as well as common crops such as wheat (*Triticum aestivum* L.) and fava bean (*Vicia faba*). Additionally, it will examine crops with weedy characteristics, including wild oat (*Avena fatua*) and wild rye (*Secale cereale* L.). In the summer of 2023, weed seeds were collected and prepared at the Moghan Research and Natural Resources Center (48°20′ E, 38°19′ N). The seeds were provided from ripening inflorescences in the fields, air-dried at a controlled room temperature of 25±2°C for three weeks, and then separated from the inflorescences by means of rubbing. Germination tests on freshly harvested seed showed that redroot pigweed, rye, oat, and wild mustard had high primary dormancy. We broke this primary seed dormancy of redroot pigweed, wild mustard, and oat seeds by treating them with gibberellic acid at 1500, 1000, or 600 ppm, respectively [50,51,52]. In this study, weed seeds were stored in a dry environment at a room temperature of (25±2°C) for 7 months leading up to the experimentation phase. The wheat seeds of the Cascogen variety (*Triticum aestivum*, Aras Bazr, Florimond Desprez, 1.1.1.67) were obtained from the Seed and Plant Improvement Institute of Iran, introduced in 1991. However, fava bean seeds of the Shadan variety (*Vicia faba* L. cv. Shadan/G-Faba–133) were also obtained from the Seed and Plant Improvement Institute of Iran, where it was introduced in 2017. Both seeds were provided from the Moghan Research and Natural Resources Center a month before the experiment and stored in a cool, dark environment.

Plant extract

In the spring of 2024, vegetative tissues of Russian knapweed were gathered from wheat fields affected by infection, located at a latitude of 38°13′ N, longitude of 48°18′ E, and an elevation of 1367 meters above sea level. The collected samples were fully dried at room temperature (25 ± 2°C) for 30 days, due to the instability of phenolics when exposed to high oven temperatures [53]. Romania-type ~3M mill was used to prepare Russian knapweed powder. The extract was prepared using the maceration method described in Ahmadnia et al., [47,54] and Trusheva et al., [55]. In short, specified amounts of Russian knapweed powder (0, 20, 40, 60, 80, 100, 120, 140, 160, 180, and 200 grams) were mixed with 1000 mL of distilled water. The solution was then placed on a shaker for 24 hours. The solution was filtered through Whatman No. 42 filter paper and centrifuged for 15 minutes at 5000 rpm to remove the solids. The separated supernatant, referred to hereafter as the Russian knapweed extract, was stored in the refrigerator at 4 °C until germination tests were performed.

Experimental design

The research was performed in 2024 at Weed Science Laboratory of the Faculty of Agriculture and Natural Resources at Mohaghegh Ardabili University. Its objective was to assess the impact of Russian knapweed extract on germination and growth characteristics of various weed and crop species. It was designed as a factorial experiment based on a completely randomized design, with three replications. Experimental treatments included different concentrations of Russian knapweed aqueous extracts (0, 20, 40, 60, 80, 100, 120, 140, 160, 180 and 200 g L^{-1}) on three problematic weeds (*Amaranthus retroflexus, Sinapis arvensis, and Avena fatua*), two commonly grown crops (*Triticum aestivum* L. and *Vicia faba*), and a cultivated cover crops that can act as volunteer plants or weeds in fields (*Secale cereale* L.). Distilled water was used as a control treatment for zero concentration. Before experimenting, the seeds of *Amaranthus retroflexus, Sinapis arvensis, Avena fatua, Secale cereale* L., *Triticum aestivum* L., and *Vicia faba* were sterilized with 1% sodium hypochlorite and washed at least three times with distilled water. In technical triplicate, sixty healthy sterile seeds were randomly placed in Petri dishes with a diameter of 9 cm (for small seeds) and 15 cm (for fava bean seeds) on No. 42 filter paper wetted with 10 ml of different concentrations of extract or distilled water in a Petri dish. Dishes were then sealed in a transparent plastic bag, and transferred to a BINDER KBW 240 germinator (Germany) set at 25°C without light. The

germination of the seeds was monitored for 14 days, at the same time each day, and if needed, an additional 10 ml of different concentrations of extract or distilled water was added to ensure that they did not dry out. Seeds were classified as germinated when the radicle measured 2 mm in length [56]. Measurements of seed growth traits were conducted on day 14.

Germination measurements

The germination percentage (GP) and germination rate (GR) were recorded daily, allowing for the calculation of mean germination time (MGT), mean daily germination ($\bar{G}$), germination index (GI), peak value (PV), and germination value (GV). Measurements of seedling radicle and plumule lengths, along with fresh and dry weights of the seedlings, were taken 14 days after incubation at a temperature of 25°C. Protocols and Equations are further described in [57] or elsewhere as indicated.

Germination percentage (GP)

Equation 1, described by Scott et al. [58], Burnett et al. [59], and MacGregor et al. [60] was used to calculate germination percentage (GP) which equals the ratio of the number of germinated seeds (S) over the total number of seeds in the experimental sample (T).

$$\mathrm{GP} = \frac{\mathrm{S}}{\mathrm{T}} \times 100 \qquad (1)$$

Germination rate (GR)

Equation 2 from Maguire [61] was used to calculate the germination rate (GR) which is the number of germinated seeds over time.

$$\mathrm{GR} = \frac{Number\ of\ normal\ seedlings}{Days\ to\ first\ count} + \cdots \frac{Number\ of\ normal\ seedlings}{Days\ to\ final\ count} \qquad (2)$$

Germination index (GI)

Equation 3 described by TeKrony and Egli, [62] generates the germination index (GI) where ni is the total number of germinated seeds and ti is the number of days after germination.

$$\mathrm{GI} = \sum_{\mathrm{i}=1}^{\mathrm{k}} \mathrm{ni}/\mathrm{ti} \qquad (3)$$

Mean daily germination ($\bar{G}$)

Equation (4), described by TeKrony & Egli, [62] generates mean daily germination ($\bar{G}$). It represents the mean number of seeds germinated per day.

$$\overline{G} = \frac{GP}{T_n} \quad (4)$$

$\bar{G}$ is the ratio of GP, thefinal cumulative germination percentage over T_n ,the total number of days required for final germination.

Peak value (PV)

Equation 5 described by Saeb et al. [63] was used to generate peak value (PV) which is the accumulated number of seeds that have germinated at the point on the germination curve where the rate of germination starts to decrease. It is calculated by finding the highest ratio of cumulative germination to the corresponding incubation time.

$$PV = \frac{MDG_{max}}{N_d} \quad (5)$$

Where, PV is the ratio of maximum mean daily germination (MDG_{max}) over the number of days after germination (N_d).

Germination value (GV)

Equation (6) described by Czabator [64] was used to calculate germination value (GV).

$$GV = MDG \times PV \quad (6)$$

Where, germination value (GV), is the mean daily germination (MDG) multiplied by peak value (PV).

Mean germination time (MGT)

Equation (7) described by Ellis, [65] was used to calculate the mean germination time (MGT).

$$MGT = \frac{\sum(nd)}{\sum n} \quad (7)$$

The mean time required for germination (MGT), was calculated by the ratio of the sum of number of days counted from the start of the experiment (d), divided by the sum of n which represents the number of germinated seeds on day d.

Seedling radicle and plumule length

Radicle, and plumule lengths were taken on day 14 and were measured using a centimeter ruler with an accuracy of 1 mm.

Statistical analysis

As in Ahmadnia et al. [57], the data were analyzed using RStudio and SAS 9.4 software where $LSD_{5\%}$ were used to define any differences among the treatments Pearson's correlation coefficient was used to define relationships between traits, and we used the R corrplot package within RStudio software to do this.

Gas Chromatography–Mass Spectrometry (GC-MS)

Five grams of Russian knapweed powder were shaken in 50 ml of deionized water at 25±2°C for 24 hours. The solution was then ultrasound waves (Ultrasonic Homogenizer, UP100H, manufactured by Hielscher, Germany) for 15 minutes [66] and filtered through Whatman No. 54 paper using vacuum filtration (ROCKER 811 Oil-Free Vacuum Pump, AC220V/50Hz, manufactured by QTE Technologies, Taiwan). The extract was dried at 25±2°C for 7 days, followed by freeze-drying ($VaCo_5$ ZiRBUS, manufactured by ZIBRUS technology GmbH, Germany) (Adapted from the methods of [67,68] with slight modifications). The resulting solid was dissolved in 10 mL deionized water containing 0.5% DMSO, filtered through a 0.22 µm Sarstedt filter, and immediately analyzed by GC-MS. An Agilent 7890B Gas Chromatograph coupled with an Agilent 5977A Mass Selective Detector (MSD), manufactured by Agilent Technologies Inc., Palo Alto, USA, was employed for analysis. Samples (0.1 µL) were injected using helium as the carrier gas. The multimode inlet (MMI) operated in split mode (15:1 split ratio, 48 mL/min flow rate). The MMI temperature program commenced at 100 °C, increasing to 380 °C at a rate of 8 °C per minute. The oven temperature began at 40 °C, ramped up to 325 °C at 4 °C/min, and then to 400 °C at 10 °C/min, where it was maintained for 12.5 minutes. The entire analysis lasted 46.95 minutes.

Results

Russian knapweed extracts significantly reduced the measured germination parameters germination percentage (Fig 1a, Table 1), germination rate (Fig 1b, Table 1), germination index (Table 1) of the seeds of all species tested in a concentration-dependent manner ($P < 0.01$) (Table 2). Mean germination time was largely unaffected by the application of extract which is reflected in a very low analysis of variance effect of experimental treatments on mean germination time (Tables 1 and 2, Fig 1c). Likewise, seedling parameters {radicle length (Fig 1d) and plumule length (Figure 1e)} of the seeds of all species tested were also reduced in a concentration-dependent

manner ($P \leq 0.01$) (Tables 1 and 2). Each of the species responded to Russian knapweed extract and although the effective concentration was species-specific, the responses were all roughly linear (Table 3) and the relationship is directly proportional to the concentration of the allelopathic extracts applied in the treatment (Tables 1-3, Fig 1). Analyzing the data relative to the control shows that although all species responded to the Russian knapweed extract, their behaviors split into two types which are differentiated by the slope of the response as concentration increased – the more sensitive type includes *Amaranthus retroflexus*, and *Sinapis arvensis*, while the less sensitive type includes *Triticum aestivum, Vicia faba,* and *Secale cereal* (Tables 1 and 3, Fig 1). The response of *Avena fatua* was intermediate and the data show that although some seedling can still germinate at ≥100 g L^{-1}, these seedlings were smaller, with lower plumule or radicle lengths (Table 1 and 3, Fig 1). *Sinapis arvensis* seeds were particularly sensitive to Russian knapweed extract and concentrations above 20 g L^{-1} fully inhibited germination, therefore there are less data to analyze (Tables 1-3, Fig 1).

280

281

Table 1. The interaction effect of concentration × plant type on germination percentage, germination rate, mean germination time, seedling plumule length, seedling radicle length, germination index, mean daily germination, peak value and G value.

Plant	Concentration ($g\ L^{-1}$)	Germination percentage (%)	Germination rate (Seed/day)	Mean germination time (Seed/day)	Seedling plumule length (cm)	Seedling radicle length (cm)	Germination index	Mean daily germination	Peak value	G value
Amaranthus retroflexus	0	100.00 ± 0.00	14.86± 0.07	1.55 ± 0.02	3.37 ± 0.09	2.09 ± 0.26	14.86 ± 0.07	7.14 ± 0.00	50.00 ± 0.00	357.14 ± 0.00
	20	100.00 ± 0.00	8.30 ± 0.21	2.51 ± 0.07	3.12 ± 0.52	1.16 ± 0.12	8.30 ± 0.21	7.14 ± 0.00	32.77 ± 0.55	234.13 ± 3.96
	40	20.00 ± 0.00	1.00 ±0.00	4.66 ± 0.16	1.53 ± 0.06	0.50 ± 0.05	1.00 ± 0.00	1.42 ± 0.00	3.17 ± 0.15	4.53 ± 0.22
	60	1.66 ± 1.66	0.05 ± 0.05	2.00 ± 2.00	0.00 ± 0.00	0.03 ± 0.03	0.05 ± 0.05	0.11± 0.11	0.27 ± 0.27	0.09 ± 0.09
	80	0.00 ± 0.00	0.00 ± 0.00	0.00 ± 0.00	0.00 ± 0.00	0.00 ± 0.00	0.00 ± 0.00	0.00 ± 0.00	0.00 ± 0.00	0.00 ± 0.00
	100	0.00 ± 0.00	0.00 ± 0.00	0.00 ± 0.00	0.00 ± 0.00	0.00 ± 0.00	0.00 ± 0.00	0.00 ± 0.00	0.00 ± 0.00	0.00 ± 0.00
	120	0.00 ± 0.00	0.00 ± 0.00	0.00 ± 0.00	0.00 ± 0.00	0.00 ± 0.00	0.00 ± 0.00	0.00 ± 0.00	0.00 ± 0.00	0.00 ± 0.00
	140	0.00 ± 0.00	0.00 ± 0.00	0.00 ± 0.00	0.00 ± 0.00	0.00 ± 0.00	0.00 ± 0.00	0.00 ± 0.00	0.00 ± 0.00	0.00 ± 0.00
	160	0.00 ± 0.00	0.00 ± 0.00	0.00 ± 0.00	0.00 ± 0.00	0.00 ± 0.00	0.00 ± 0.00	0.00 ± 0.00	0.00 ± 0.00	0.00 ± 0.00
	180	0.00 ± 0.00	0.00 ± 0.00	0.00 ± 0.00	0.00 ± 0.00	0.00 ± 0.00	0.00 ± 0.00	0.00 ± 0.00	0.00 ± 0.00	0.00 ± 0.00
	200	0.00± 0.00	0.00± 0.00	0.00 ± 0.00	0.00 ± 0.00	0.00 ± 0.00	0.00 ± 0.00	0.00 ± 0.00	0.00 ± 0.00	0.00 ± 0.00
Secale cereal L.	0	100.00 ± 0.00	9.13± 0.10	2.30 ± 0.02	7.00 ± 0.01	8.50 ± 0.09	9.13 ± 0.10	7.14 ± 0.00	39.16 ± 1.66	279.76 ± 11.90
	20	86.66 ± 3.33	7.95 ± 0.10	2.31 ± 0.09	5.91 ± 0.06	7.42 ± 0.08	7.95 ± 0.10	6.19 ± 0.23	35.00 ± 1.44	216.07 ± 4.72
	40	86.66 ± 1.66	7.83 ± 0.12	2.30 ± 0.01	5.12 ± 0.09	6.19 ± 0.09	7.83 ± 0.12	6.19 ± 0.11	31.66 ± 0.83	196.13 ± 7.75
	60	73.33 ± 1.66	6.52 ± 0.03	2.44 ± 0.11	4.59 ± 0.14	5.03 ± 0.03	6.52 ± 0.03	5.23 ± 0.11	27.50 ± 1.44	143.75 ± 4.97
	80	61.66 ± 1.66	5.86 ± 0.12	2.16 ± 0.04	3.62 ± 0.06	4.16 ± 0.07	5.86 ± 0.12	4.40 ± 0.11	26.66 ± 0.83	117.56 ± 5.93
	100	51.66 ± 1.66	4.88 ± 0.14	2.16 ± 0.03	3.16 ± 0.08	3.12 ± 0.01	4.88 ± 0.14	3.69 ± 0.11	21.66 ± 0.83	80.05 ± 4.89
	120	40.00 ± 0.00	3.72 ± 0.11	2.20 ± 0.08	2.99 ± 0.008	2.87 ± 0.05	3.72 ± 0.11	2.85 ± 0.00	16.11 ± 1.38	46.03 ± 3.96
	140	21.66 ± 1.66	1.83 ± 0.16	2.46 ± 0.03	2.82 ± 0.06	1.77 ± 0.11	1.83 ± 0.16	1.54 ± 0.11	7.22 ± 0.55	11.30 ± 1.78
	160	0.00 ± 0.00	0.00 ± 0.00	0.00 ± 0.00	0.00 ± 0.00	0.00 ± 0.00	0.00 ± 0.00	0.00 ± 0.00	0.00 ± 0.00	0.00 ± 0.00
	180	0.00 ± 0.00	0.00 ± 0.00	0.00 ± 0.00	0.00 ± 0.00	0.00 ± 0.00	0.00 ± 0.00	0.00 ± 0.00	0.00 ± 0.00	0.00 ± 0.00
	200	0.00 ± 0.00	0.00 ± 0.00	0.00 ± 0.00	0.00 ± 0.00	0.00 ± 0.00	0.00 ± 0.00	0.00 ± 0.00	0.00 ± 0.00	0.00 ± 0.00
Avena fatua	0	100.00 ± 0.00	14.33± 0.83	1.80 ± 0.11	21.03 ±0.93	13.40 ± 0.87	14.33 ± 0.83	7.14 ± 0.00	53.33 ± 8.81	380.95 ± 62.99
	20	81.66 ± 4.40	11.83 ±0.94	1.67 ± 0.06	19.50 ± 0.57	8.20 ± 0.15	11.83 ± 0.94	5.83± 0.31	41.66 ± 4.40	245.83 ± 39.61
	40	71.66 ± 1.66	9.72 ± 0.68	2.05 ± 0.19	9.83 ± 0.20	2.66 ± 0.16	9.72 ± 0.68	5.11 ± 0.11	35.00± 2.88	179.76 ± 18.70
	60	60.00 ± 2.88	7.27 ± 0.45	1.98 ± 0.20	6.00 ± 0.57	1.20 ± 0.15	7.27 ± 0.45	4.28 ± 0.20	25.83 ± 0.83	110.42 ± 2.83
	80	48.33 ± 1.66	4.38 ± 0.30	2.31 ± 0.17	3.63 ± 0.31	1.16 ± 0.16	4.38 ± 0.30	3.45 ± 0.11	18.33 ± 3.33	63.69 ± 12.89
	100	18.33 ± 1.66	1.66 ± 1.66	2.33 ± 0.33	1.30 ± 0.30	0.66 ± 0.16	1.66 ± 0.16	1.30 ± 0.11	7.50 ± 1.44	9.82 ± 2.24
	120	0.00 ± 0.00	0.00 ± 0.00	0.00 ± 0.00	0.00 ± 0.00	0.00 ± 0.00	0.00 ± 0.00	0.00 ± 0.00	0.00 ± 0.00	0.00 ± 0.00
	140	0.00 ± 0.00	0.00 ± 0.00	0.00 ± 0.00	0.00 ± 0.00	0.00 ± 0.00	0.00 ± 0.00	0.00 ± 0.00	0.00 ± 0.00	0.00 ± 0.00
	160	0.00 ± 0.00	0.00 ± 0.00	0.00 ± 0.00	0.00 ± 0.00	0.00 ± 0.00	0.00 ± 0.00	0.00 ± 0.00	0.00 ± 0.00	0.00 ± 0.00
	180	0.00 ± 0.00	0.00 ± 0.00	0.00 ± 0.00	0.00 ± 0.00	0.00 ± 0.00	0.00 ± 0.00	0.00 ± 0.00	0.00 ± 0.00	0.00 ± 0.00
	200	0.00 ± 0.00	0.00 ± 0.00	0.00 ± 0.00	0.00 ± 0.00	0.00 ± 0.00	0.00 ± 0.00	0.00 ± 0.00	0.00 ± 0.00	0.00 ± 0.00
Sinapis arvensis	0	100.00 ± 0.00	8.27± 0.18	2.68 ± 0.04	7.50 ± 0.57	10.43 ± 0.44	8.27 ± 0.18	7.14 ± 0.00	31.11 ± 0.55	222.22 ± 3.96
	20	16.66 ± 6.00	0.86 ± 0.39	4.86 ± 0.94	2.46 ± 0.53	1.03 ± 0.03	0.86 ± 0.39	1.19 ± 0.42	3.13 ± 1.22	4.60 ± 2.16
	40	0.00 ± 0.00	0.00 ± 0.00	0.00 ± 0.00	0.00 ± 0.00	0.00 ± 0.00	0.00 ± 0.00	0.00 ± 0.00	0.00 ± 0.00	0.00 ± 0.00
	60	0.00 ± 0.00	0.00 ± 0.00	0.00 ± 0.00	0.00 ± 0.00	0.00 ± 0.00	0.00 ± 0.00	0.00 ± 0.00	0.00 ± 0.00	0.00 ± 0.00
	80	0.00 ± 0.00	0.00 ± 0.00	0.00 ± 0.00	0.00 ± 0.00	0.00 ± 0.00	0.00 ± 0.00	0.00 ± 0.00	0.00 ± 0.00	0.00 ± 0.00
	100	0.00 ± 0.00	0.00 ± 0.00	0.00 ± 0.00	0.00 ± 0.00	0.00 ± 0.00	0.00 ± 0.00	0.00 ± 0.00	0.00 ± 0.00	0.00 ± 0.00
	120	0.00 ± 0.00	0.00 ± 0.00	0.00 ± 0.00	0.00 ± 0.00	0.00 ± 0.00	0.00 ± 0.00	0.00 ± 0.00	0.00 ± 0.00	0.00 ± 0.00
	140	0.00 ± 0.00	0.00 ± 0.00	0.00 ± 0.00	0.00 ± 0.00	0.00 ± 0.00	0.00 ± 0.00	0.00 ± 0.00	0.00 ± 0.00	0.00 ± 0.00
	160	0.00 ± 0.00	0.00 ± 0.00	0.00 ± 0.00	0.00 ± 0.00	0.00 ± 0.00	0.00 ± 0.00	0.00 ± 0.00	0.00 ± 0.00	0.00 ± 0.00
	180	0.00 ± 0.00	0.00 ± 0.00	0.00 ± 0.00	0.00 ± 0.00	0.00 ± 0.00	0.00 ± 0.00	0.00 ± 0.00	0.00 ± 0.00	0.00 ± 0.00
	200	0.00 ± 0.00	0.00 ± 0.00	0.00 ± 0.00	0.00 ± 0.00	0.00 ± 0.00	0.00 ± 0.00	0.00 ± 0.00	0.00 ± 0.00	0.00 ± 0.00

Triticum aesticum L.	0	100.00 ± 0.00	9.44± 0.14	2.18 ± 0.06	7.15 ± 0.02	7.21 ± 0.11	9.44 ± 0.14	7.14 ± 0.00	42.50 ± 1.44	303.57 ± 10.30
	20	86.66 ± 1.66	8.08 ± 0.20	2.21 ± 0.02	7.00 ± 0.01	6.40 ± 0.07	8.08 ± 0.20	6.19 ± 0.11	35.00 ± 1.44	216.66 ± 12.82
	40	75.00 ± 0.00	6.88 ± 0.05	2.24 ± 0.02	6.34 ± 0.02	5.69 ± 0.08	6.88 ± 0.05	5.35 ± 0.00	28.33 ± 0.83	151.79 ± 4.46
	60	61.66 ± 1.66	5.61 ± 0.19	2.29 ± 0.03	5.97 ± 0.04	5.28 ± 0.02	5.61 ± 0.19	4.40 ± 0.11	23.33 ± 0.83	102.98 ± 6.54
	80	43.33 ± 3.33	4.08 ± 0.25	2.18 ± 0.05	5.65 ± 0.11	4.33 ± 0.05	4.08 ± 0.25	3.09 ± 0.23	18.33 ± 0.83	57.14 ± 7.14
	100	40.00 ± 2.88	3.88 ± 0.24	2.07 ± 0.03	5.13 ± 0.06	3.52 ± 0.07	3.88 ± 0.24	2.85 ± 0.20	18.33 ± 0.83	52.67 ± 6.07
	120	31.66 ±1.66	3.11 ± 0.20	2.05 ± 0.05	4.06 ± 0.034	2.12 ± 0.01	3.11 ± 0.20	2.26 ± 0.11	15.00 ± 1.44	34.22 ± 5.00
	140	0.00 ± 0.00	0.00 ± 0.00	0.00 ± 0.00	0.00 ± 0.00	0.00 ± 0.00	0.00 ± 0.00	0.00 ± 0.00	0.00 ± 0.00	0.00 ± 0.00
	160	0.00 ± 0.00	0.00 ± 0.00	0.00 ± 0.00	0.00 ± 0.00	0.00 ± 0.00	0.00 ± 0.00	0.00 ± 0.00	0.00 ± 0.00	0.00 ± 0.00
	180	0.00 ± 0.00	0.00 ± 0.00	0.00 ± 0.00	0.00 ± 0.00	0.00 ± 0.00	0.00 ± 0.00	0.00 ± 0.00	0.00 ± 0.00	0.00 ± 0.00
	200	0.00 ± 0.00	0.00 ± 0.00	0.00 ± 0.00	0.00 ± 0.00	0.00 ± 0.00	0.00 ± 0.00	0.00 ± 0.00	0.00 ± 0.00	0.00 ± 0.00
Vicia faba	0	100.00 ± 0.00	4.88 ± 0.25	4.23 ± 0.24	3.16 ± 0.16	10.76 ± 0.81	4.88 ± 0.25	7.14 ± 0.00	20.16 ± 1.30	144.05 ± 9.29
	20	83.33 ± 1.66	3.39 ± 0.15	4.99 ± 0.30	4.66 ± 0.16	14.83 ± 1.42	3.39 ± 0.15	5.92 ± 0.11	15.16 ± 1.36	90.17 ± 7.91
	40	71.66 ± 1.66	2.85 ± 0.12	5.11 ± 0.16	4.33 ± 0.44	15.16 ±1.69	2.85 ± 0.12	5.11 ± 0.11	12.00 ± 1.00	61.54 ± 5.92
	60	63.33 ± 4.40	2.47 ± 0.20	5.24 ± 0.15	3.83 ± 0.16	10.50 ± 1.00	2.47 ± 0.20	4.52 ± 0.31	9.53 ± 0.78	43.61 ± 6.43
	80	45.00 ± 2.88	1.43 ± 0.12	6.41 ± 0.10	3.46 ± 0.48	9.16 ± 0.16	1.43 ± 0.12	3.21 ± 0.20	6.42 ± 0.41	20.83 ± 2.65
	100	31.33 ± 4.40	1.07 ± 0.15	6.01 ± 0.10	2.63 ± 0.31	8.66 ± 0.88	1.07 ± 0.15	2.26 ± 0.31	4.52 ± 0.62	10.62 ± 2.96
	120	16.66 ± 1.66	0.53 ± 0.04	6.27 ± 0.14	2.50 ± 0.50	6.66 ± 0.83	0.53 ± 0.04	1.19 ± 0.11	2.38 ± 0.23	2.89 ± 0.59
	140	8.33± 1.66	0.30 ± 0.05	5.50 ± 0.28	1.90 ± 0.05	5.26 ± 0.66	0.30 ± 0.05	0.59 ± 0.11	1.36 ± 0.19	0.85 ± 0.25
	160	3.33± 1.66	0.09 ± 0.04	4.66 ± 2.33	0.63 ± 0.31	3.00 ± 1.52	0.09 ± 0.04	0.23 ± 0.11	0.47 ± 0.23	0.17 ± 0.08
	180	1.66± 1.66	0.04± 0.04	2.33 ± 2.33	0.30 ± 0.30	1.06 ± 1.06	0.04 ± 0.04	0.11 ± 0.11	0.23 ± 0.23	0.08 ± 0.08
	200	3.33± 1.66	0.09 ± 0.04	4.66 ± 2.33	0.00	1.10 ± 0.05	0.09 ± 0.04	0.23 ± 0.11	0.47 ± 0.23	0.17 ± 0.08
	LSD (*P*<0.05)	4.80	0.61	1.61	0.67	1.25	0.61	0.34	4.18	29.05

Table 2. Analysis of variance (Mean Squares) the effect of experimental treatments on germination percentage, germination rate, mean germination time, seedling plumule length, and seedling radicle length.

Source of Variation	df	Germination percentage	Germination rate	Mean germination time	Seedling plumule length	Seedling radicle length	Germination index	Mean daily germination	Peak value	G value
Concentration	10	20246.23**	191.84**	5.40**	143.81**	14.82**	191.84**	103.29**	2904.47**	531.68**
Plant type	5	6284.44**	77.85**	12.62**	110.41**	23.16**	77.85**	32.06**	1373.31**	192.65**
Concentration × Plant type	50	804.38**	13.25**	0.77**	24.03**	1.00**	13.25**	4.10**	174.97**	24.65**
Error	132	8.83	0.14	0.14	0.17	0.05	0.14	0.04	6.72	0.38
Coeff Var (%)	-	9.31	13.38	25.63	14.97	18.82	13.38	9.31	22.49	12.36

**Significant at 1% probability level.

Table 3. Linear slope and Pearson's R^2 values calculated from non-zero datapoints of germination and seedling parameters

Value-Slope	*Sinapis arvensis*	*Amaranthus retroflexus*	*Avena fatua*	*Vicia faba*	*Triticum aestivum*	*Secale cereale*
Percent Germination	-0.24	-0.46	-1.27	-1.78	-1.64	-1.81
Germination Rate	-2.66	-3.59	-7.60	-37.94	-17.74	-19.79
Mean Germination Time	5.25	2.54	54.60	-4.09	-195.38	-6.95
Germination Index	-2.66	-3.59	-7.60	-37.94	-17.74	-19.79
Mean Daily Germination Time	-3.29	-6.46	-17.83	-24.94	-22.95	-25.28
Peak value	-1.05	-1.04	-1.45	-6.00	-4.04	-4.47
G value	-0.09	-0.14	-0.24	-1.20	-0.41	-0.53
Plumule length	-3.62	-14.77	-4.32	-34.82	-38.82	-31.08
Radicle length	-2.11	-26.50	-6.40	-11.44	-24.40	-20.64
**Value-R^2**	***Sinapis arvensis***	***Amaranthus retroflexus***	***Avena fatua***	***Vicia faba***	***Triticum aestivum***	***Secale cereale***
Percent Germination	0.98	0.87	0.95	0.93	0.97	0.97
Germination Rate	0.99	0.93	0.96	0.88	0.96	0.96
Mean Germination Time	0.57	0.04	0.35	0.02	0.25	0.00
Germination Index	0.99	0.93	0.96	0.88	0.96	0.96
Mean Daily Germination Time	0.98	0.87	0.95	0.93	0.97	0.97
Peak value	0.82	0.93	0.65	0.92	0.91	0.93
G value	1.00	0.90	0.85	0.74	0.90	0.98
Plumule length	0.91	0.86	0.93	0.78	0.95	0.94
Radicle length	0.99	0.91	0.79	0.79	0.97	0.98

Fig 1. The effect of Russian Knapweed extract on percent germination (a), germination rate (b), mean germination time (c), radicle length (d), and plumule length (e). Data are shown as averages ± standard deviation of 20 plants observed for 14 days as described in the methods. For c, d and e, the data are presented as relative to the control values.

Germination percentage

In the control treatment, 100% of seeds of *Amaranthus retroflexus*, *Secale cereal*, *Vicia faba, Triticum aestivum,* and 98.33% of *Sinapis arvensis* seeds germinated (Table 1, Fig 1a). The minimum dosage (20 g L^{-1}) exhibited a moderate impact on the germination of all seeds except for *Amaranthus retroflexus,* which showed 100% germination at this rate and Sinapis arvensis which was strongly inhibited even at this low dose (Table 1, Fig 1a). Extract concentrations above 160 g L^{-1} fully the inhibited germination of *Amaranthus retroflexus*, *Avena fatua*, *Triticum aestivum* and *Secale cereal* seeds while *Vicia faba* had a low-level germination at all doses studied. All studied species and crops were sensitive to Russian knapweed extracts, although these dose-response experiments show some were more sensitive than others. As all the other parameters were dependent on whether or not the seeds germinated, data could not be collected for percentage of germination, rate of germination, time of germination, length of the plumule, length of the radicle, germination index, average daily germination, peak value, or G value for *Sinapis arvensis* > 40 g L^{-1}, *Amaranthus retroflexus* > 80 g L^{-1}, *Avena fatua* > 120 g L^{-1}, *Triticum aestivum* > 140 g L^{-1} and *Secale cereal* > 160 g L^{-1}, and *Vicia faba* > 200 g L^{-1}.

Germination rate

Aqueous extracts of Russian knapweed significantly inhibited germination rates of all tested species. As shown in Table 1 and Fig 1b, aerial extracts of Russian knapweed either entirely inhibited or partially diminished the germination rates, where impact was dependent on the concentration of extract. *Amaranthus retroflexus* and *Avena fatua* had the highest mean germination rate under control conditions (Fig 1b, Table 1). While germination rate of *Amaranthus retroflexus* was rapidly decreased in the presence of any concentration of Russian knapweed extract, *Avena fatua* showed a slow decrease in rate in response to extract (Fig1b, Table 1). *Secale cereale*, *Triticum aestivum*, and *Sinapis arvensis* had similar germination rates without extract and while the germination rate of *Sinapis arvensis* was strongly inhibited, the germination rates of *Secale cereale* and *Triticum aestivum* were similar and the rate was slowly inhibited with increasing doses of Russian knapweed extract (Fig 1b, Table 1). *Vicia faba* had the lowest germination rates at the control condition and like *Secale cereale* and *Triticum aestivum,* the rate of inhibition was slow (Fig 1b, Table 1).

Germination index

Data presented in Table 1 and Fig 1c show that aqueous extracts of aerial Russian knapweed inhibited the germination index. An increase in concentrations of the extract was associated with a drastic reduction of the germination index and germination index of plants varied by species and exhibited a direct proportionality to concentration of allelopathic extracts applied during the treatment (Table 1). The differences in maximum and minimum germination indexes ranked in order: *Amaranthus retroflexus* 14.86-0.06*, Avena fatua* 14.33-1.76, *Secale cereal* 9.13-1.86, *Triticum aestivum* 9.44-3.11, *Sinapis arvensis* 8.28-0.87, and *Vicia faba* 4.84-0.05. Without Russian knapweed extract, *Amaranthus retroflexus* and *Avena fatua* recorded a germination index significantly higher than other treatments but the germination index of *Amaranthus retroflexus* was reduced by 44% with as little as 20 g L^{-1} Russian knapweed extract while to reach a similar reduction in *Avena fatua* required between 60 to 80 g L^{-1} (Table 1). Russian knapweed extract affected the germination index of *Secale cereal*, and *Triticum aestivum* similarly. Like for the other parameters, germination index of *Sinapis arvensis* was dramatically reduced with low-dose concentrations, while *Vicia faba*'s germination index was the lowest under control conditions and slowly and linearly declined in response to increasing doses (Table 1).

Mean daily germination

The study revealed that increasing the extract concentrations led to a decrease in the mean daily germination in all the tested species (Table 1). The comparison of the means demonstrated that maximum mean daily germination (7.143 days) resulted in control of *Amaranthus retroflexus*, *Secale cereal*, *Avena fatua, Triticum aestivum,* and the concentration of 20 g L^{-1} in *Amaranthus retroflexus.*

Peak value

The peak value refers to the highest quotient obtained by dividing the total number of seeds that germinated at by the corresponding number of days. In other words, it represents the mean daily germination of the most vigorous component of a seed lot [64]. The influence of the Russian knapweed extracts on peak value is shown in Table 1. The highest rates of peak value were recorded in the control treatment in *Amaranthus retroflexus*.

G value

The modifications in the G value resulting from the allelopathic compounds found in the aqueous extracts of Russian knapweed exhibited notable variability among different plant species. A significant reduction in the G value was observed in all treatments and for every plant species examined. The greatest rate of G value was observed in the control treatment of *Amaranthus retroflexus* (Table 1).

Mean germination time

The mean germination time exhibited the least variation among the assessed parameters due to application of Russian knapweed extract. Nevertheless, the use of this extract did produce a noticeable effect. Although application of Russian knapweed extract increased mean germination time for *Amaranthus retroflexus* at 40 g L^{-1} threefold and *Sinapis arvensis* at 20 g L^{-1} twofold, the other species had mean germination rates that were within 1.5-fold of control (Fig 1c, Table 1). For each species, no statistical difference in mean germination time was seen when any concentration of extract was applied compared to control (Tables 1 and 2).

Seedling plumule and radicle lengths

Increasing concentrations of Russian knapweed extract reduced both seedling radicle and plumule lengths gradually in each of the species (Fig 1d and e). The control treatment showed that *Avena fatua* were the longest, then *Sinapis arvensis, Triticum aestivum,* and *Secale cereal* had similar-length plumules, which were all bigger than *Vicia faba, and Amaranthus retroflexus* (Fig 1e, Table 1). Radicle lengths differed more between the species; from the longest *Avena fatua,* then *Sinapis arvensis*, and *Vicia faba,* then similarly sized *Secale cereal,* and *Triticum aestivum*, then *Amaranthus retroflexus* as the smallest (Fig 1d, Table 1). In each species, the reduction in length was roughly linear for either plumule or radicle lengths. The square of the Pearson product-moment correlation coefficient (R^2) was greater than 0.78 for plumule or 0.79 for radicle (Table 3). The Russian knapweed extracts reduced seedling plumule or radicle lengths in a concentration-dependent manner, but the sensitivity differed between the species. Interestingly the sensitivity of these seedling parameters to Russian knapweed extract divides along weedy/cultivated boundaries where weedy species were more sensitive than cultivated species for all measured parameters (Fig 1 d and e).

Correlation

The results of Pearson's correlation analysis indicated that the germination rates of both weed and crop seeds were influenced by varying concentrations of Russian knapweed aqueous extract, demonstrating a strong positive correlation with mean daily germination (r=0.92**), germination percentage (r=0.92**), germination index (r=0.99**), germination rate (r=0.99**), G value (r=0.97**), seedling plumule length (r=0.82**), seedling radicle length (r=0.57**), and mean germination time (r=023**) effect (Fig 2).

Fig 2. Pearson's correlation coefficients (n = 198) among eleven quantitative traits on *Amaranthus retroflexus*, *Secale cereal*, *Avena fatua*, *Sinapis arvensis*, *Triticum aestivum*, and *Vicia faba*. The correlation coefficients were significant at the statistical probability level of 1 and 5%. GP, germination percentage; GR, germination rate; MGT, Mean germination time; MDG, Mean daily germination; SPL, Seedling plumule length; SRL, Seedling radicle length; GI, Germination index; PV, Peak value; GV, Germination value.

Untargeted GC-MS results

To gain mechanistic insight into what allelopathic compound or compounds are within the Russian knapweed extract, we used untargeted GC-MS profiling of the aqueous extract. GC-MS analysis of the derivatized aqueous extract identified several compounds with reported allelopathic or phytotoxic activity, including carvacrol, phenol, benzenediols, anisaldehyde, and organic acids such as propanoic and acetic acid (Table 4). The two compounds with the largest peak areas were stigmasterol and methanesulfonic acid (Table 4). Several other small organic acids, silicone and organophosphorus compounds, and other compounds were also identified (Table 4). These findings support the hypothesis that allelopathic secondary metabolites that could contribute to its inhibitory effects are present in the Russian knapweed extract.

Table 4. Untargeted GC-MS results of Russian knapweed

Chemical Class	Functional Group / Bioactivity	Compound Name	Molecular Formula	Retention Time (min)
Pyridine Derivatives	Alkylated Pyridines	Conyrine	$C_8H_{11}N$	21.146
	Hydroxyalkyl Pyridines	2-Pyridinepropanol	$C_8H_{11}NO$	20.479
	Dimethyl Pyridines	2,5-Lutidine	C_7H_9N	21.227
Tricyclic Aromatic Compounds	Acridine Derivatives	2-Ethylacridine	$C_{15}H_{13}N$	45.636
Amino Acids & Derivatives	Imines	Prolinal	C_5H_9NO	16.613
Monoterpenoids	Phenolic Monoterpenes	Carvacrol	$C_{10}H_{14}O$	18.822
Steroids	Phytosterols	Stigmasterol	$C_{29}H_{48}O$	42.671
	Spirostanols	Diosgenin	$C_{27}H_{42}O_3$	27.263
Simple Hydrocarbons	Alkanes	Methane	CH_3	18.281
Heterocyclic Compounds	Furan Derivatives	Furan	C_4H_4O	20.13
Phenolic Compounds	Simple Phenols	Phenol	C_6H_6O	17.175
		Carbolic acid	C_6H_6O	15.906
	Dihydroxybenzenes	Pyrocatechol	$C_6H_6O_2$	27.195
		Benzenediol	$C_6H_6O_2$	17.519
Polycyclic Aromatic Compounds	Thioanthraquinones	1-Methylthioanthraquinone	$C_{16}H_{10}OS$	21.788
Oxygenated Terpenoids	Sesquiterpenoids	Viminalol	$C_{10}H_{18}O$	45.97
Pyridine Derivatives	Aldehyde Pyridines	Isonicotinaldehyde	C_6H_5NO	17.936
Aromatic Aldehydes	Methoxybenzaldehydes	Anisaldehyde	$C_8H_8O_2$	18.331
Carboxylic Acids	Short-chain Fatty Acids	Propanoic acid	$C_3H_6O_2$	16.981
	Dicarboxylic Acids	Butanedioic acid	$C_4H_6O_4$	19.436
	Monocarboxylic Acids	Acetic acid	$C_2H_4O_2$	26.691
	Aromatic Dicarboxylic Acids	Phthalic acid	$C_8H_6O_4$	39.835
Aromatic Amines	Diaminobenzoic Acids	Diaminobenzoic acid	$C_7H_8N_2O_2$	18.526
Esters	Aromatic Esters	Benzyl acetate	$C_9H_{10}O_2$	16.181
Aromatic Ethers	Simple Ethers	Phenetole	$C_8H_{10}O$	27.359
Organophosphorus Compounds	Phosphorodithioates	O,O,S-Trimethyl phosphorodithioate	$C_3H_9O_2PS_2$	19.782
Acid Halides	Carbonyl Halides	Carbonic dichloride	$COCl_2$	17.742
Amides	Halogenated Amides	Chlorinated amide	$C_{14}H_{19}ClN_4O_2$	19.908
Sulfur Compounds	Anabolic Steroid Derivatives	Sulfide	S^{2-}	28
Sulfoxides	Organosulfur Compounds	Dimethyl sulfoxide	C_2H_6OS	15.077
Sulfonic Acids	Sulfonic Acids	Methanesulfonic acid	CH_4O_3S	15.974
Sulfonothioic Acids	Mercaptotriazole Derivatives	Methane sulfonothioic acid	$CH_4O_2S_2$	16.479
Sulfones	Fluorinated Oxadiazoles	MT-sulfone	$C_9H_{12}O_2S_2$	16.555
	Organosulfur Compounds	Thiomethyl sulfoxide	C_2H_6OS	17.445
Polysulfides	Cyclic Polysulfides	1,2,4,6-Tetrathiepane	$C_4H_8S_4$	23.186

Discussion

Recently, there have been calls to reduce environmental pollution, and increase healthy food production, to better human health. Heavy reliance on herbicides has increasingly raised environmental concerns [69,70], driven the evolution of herbicide-resistant populations [71,72], and non-target toxicity [3,4,52]. This has led to a collective push from the agroindustry, farmers, and consumers for more ecologically-friendly weed management protocols that are less dependent on herbicides, forcing weed researchers to reach for innovative tools for weed control from novel sources [73,25].

One solution is to use natural compounds that have safer toxicological and environmental profiles and new modes of action to provide effective control [74]. The increasing interest in natural products, including essential oils, plant extracts, and allelochemicals use, can be attributed to their short environmental half-life and low toxicity. These substances are regarded as more environmentally sustainable [75] and frequently operate through various mechanisms, thereby minimizing the likelihood of developing resistance [76]. Although there are biopesticides [77] and bio-fungicides [78] on the market, there are very few marketed bioherbicides despite the great impact that weeds have on agricultural yields and productivity [79]. To understand whether natural products can be used as bioherbicides, we and others (reviewed in [79] have explored allelopathic plants for their potential use within sustainable agriculture. The *Asteraceae* family serves as a significant reservoir of diverse phytochemicals [20,80]. This family is recognized for its allelochemicals, such as alcohols, fatty acids, phenolics, flavonoids, terpenoids, and steroids, which inhibit the reproduction, growth, and development of neighboring plant species, including weeds [81], may have a role to play here, particularly as efficacy typically increases in a concentration-dependent manner [82,83,84].

Numerous factors within the agroecosystem can affect the efficacy of bioherbicides. These factors encompass the concentration of bioactive compounds or allelochemicals, growth stage of the plants, type of formulation used, preparation of spray, method of application, soil characteristics, and various environmental conditions as well as differences in tolerance of the target species. Therefore, specific combinations need to be empirically tested. In the present research, Russian knapweed was selected as a biological herbicide due to its recognized allelopathic characteristics [20,21,22,23,25]. Our results revealed that the application of increasing concentrations of aqueous extracts from Russian knapweed significantly reduced germination

parameters including percentage, rate, index, mean daily germination, peak value, and G value as well as seedling parameters plumule length, radicle length, of *Sinapis arvensis*, *Amaranthus retroflexus*, *Avena fatua, Sinapis arvensis, Triticum aestivum, Vicia faba,* and *Secale cereal* (Fig 1). Seed germination dictates all other aspects of plant development and productivity; therefore, we focused on how Russian knapweed extract would alter germination parameters. Compared to complete or nearly complete germination in the control treatment, germination of *Sinapis arvensis* > 40 g L^{-1}, *Amaranthus retroflexus* > 80 g L^{-1}, *Avena fatua* > 120 g L^{-1}, *Triticum aestivum* > 140 g L^{-1} and *Secale cereal* > 160 g L^{-1}, and *Vicia faba* > 200 g L^{-1}, was fully inhibited at the concentration indicated (Table 3). The level of inhibition was concentration dependent and concentrations above 160 g L^{-1} had an effectively full inhibitory effect. In addition to germination inhabitation, we see that Russian knapweed extract suppresses the seedling growth as measured by radicle and plumule lengths of the species tested in a concentration-dependent manner (Figure 1 d and e). Our results were similar to previous studies e.g., Safahani and Ghooshchi [85], Alebrahim et al. [86] and Pannacci et al. [87] but here we show that weedy and cultivated species had different tolerances to Russian knapweed extract. In our data, the many weedy species, *Amaranthus retroflexus* and *Sinapis arvensis,* were most sensitive showing a steep slope of decline in response to increasing concentrations of extract compared to the cultivated species *Vicia faba* and *Triticum aestivum* (Table 2). This weedy versus cultivated separation is most clear when focusing on seedling parameters (the relative radicle or plumule length shown in Fig 1 d and e), which clearly split into two types – the more sensitive type had a steeper slope and included *Sinapis arvensis*, *Amaranthus retroflexus*, and *Avena fatua* while the less sensitive type had a more gradual decline and included *Secale cereal*, *Vicia faba* and *Triticum aestivum,* (Table 3, Fig 1). Therefore, we demonstrate that different plant species respond differently to the allelochemicals in Russian knapweed extract and interestingly the split is along plants typically thought of as "weedy" versus those considered "cultivated".

Of the compounds we putatively identified by GC-MS, stigmasterol had the largest peak area. Although one of the most common plant sterols, stigmasterol is interesting as it is published to have diverse allelopathic [88] and pharmacological properties such as anticancer, anti-osteoarthritis, anti-inflammatory, anti-diabetic, immunomodulatory, antiparasitic, antifungal, antibacterial, antioxidant, and neuroprotective activities [89]. Similar to our results, it was identified as a component in *Euphorbia heterophylla* root extracts that showed strong allelopathic

activity against indicator plants [88]. The second most abundant compound was methanesulfonic acid. Methanesulfonic acid (MSA) is a strong organic acid involved in the global sulfur cycle. Koc et al. [90] examined the effects of MSA on the subantarctic species *Colobanthus apetalus* and the Antarctic species *Colobanthus quitensis,* looking for changes in their physiology and/or biochemistry, including changes to germination capacity and germination rate. They determined that their higher doses reduced the germination rate and capacity of both species. Together, these data suggest that either or both compounds in the aqueous Russian knapweed extract may be active and repress the gemination of the species we studied here.

Flavonoids and phenolic compounds are recognized for their ability to inhibit the germination and growth of various plant species [91]. Phenol, carvacrol, and benzenediols were all putatively identified from the GC-MS of the extract (Table 4). Research has indicated that allelochemicals can prevent seed germination by inhibiting the development of the radicle and the elongation of the hypocotyl. This inhibition occurs through the production of reactive oxygen species (ROS), which can damage sub-cellular structures, disrupt protein metabolism, and interfere with phytohormones [92]. Consequently, these processes affect cell division, hormone biosynthesis, mineral uptake and transport [14], membrane permeability [15], stomatal movements, photosynthesis [16], respiration, and protein metabolism [17], ultimately leading to a reduction in auxin levels [93].

Certain allelochemicals obstruct the uptake of water and nutrients by plant roots while also inhibiting essential processes such as photosynthesis, respiration, protein synthesis, cell division, and the development of seminal roots. These effects can lead to delayed maturation and potential reproductive failure [94]. Additionally, various factors, including reduced mitotic divisions in roots, diminished activity of catalytic enzymes, and interference with the absorption of mineral ions due to the presence of allelochemicals, contribute to a decline in plant growth [95]. The elongation of cells is also affected by allelochemicals, which hinder the functioning of gibberellin and indoleacetic acid [96]. Although it is thought that Russian knapweed transfers allelochemical substance(s) to the target plant's roots at the beginning of growth and allelopathic substance(s) accumulate in the roots to decrease target plant seedling length [94], it is unclear how the active allelopathic compound(s) in Russian knapweed extract inhibiting germination as well as subsequent plant growth. Several compounds putatively identified in the Russian knapweed extract, including phenol, carvacrol, benzenediols (e.g., catechol), anisaldehyde, and short-chain

organic acids such as propanoic and acetic acid, can impair root membrane integrity, disrupt cell division and hormone signaling, and inhibit water and nutrient uptake, and could, possibly in combination, be contributing to reduced seedling vigor we observed [97, 98, 99, 100]. Similar to our work, the bioherbicidal potential of essential oil from *Thymbra capitata* (L.) Cav., where the main compound is carvacrol, was explored and the data show germination and seedling growth of *Erigeron canadensis* L., *Sonchus oleraceus* (L.) L., *Chenopodium album* L., *Setaria verticillata* (L.) P.Beauv., *Avena fatua* L., *Solanum nigrum* L., *Amaranthus retroflexus* L., *Portulaca oleracea* L., and *Echinochloa crus-galli* (L.) P.Beauv. were inhibited [101]. Further research would be required to determine which effect is, or effects are, occurring in response to the putative allelopathic compounds within the Russian knapweed extract.

Conclusion

This research evaluates the impact of aqueous extract from Russian knapweed on the germination and initial growth of problematic weed seedlings (*Sinapis arvensis*, *Amaranthus retroflexus*, and *Avena fatua*) and cultivated species (*Triticum aestivum, Vicia faba,* and *Secale cereal*) ~~was investigated.~~ We found that Russian knapweed aqueous extract was effective in inhibiting the germination of all species in a concentration-dependent manner. A similar concentration-dependent effect on seedling parameters also measured. Particularly for the seedling parameters, our results show these species split into two types, a more tolerant type (*Secale cereal*, *Triticum aestivum,* and *Vicia faba*) and a more sensitive type (*Avena fatua*, *Amaranthus retroflexus,* and *Sinapis arvensis,*); with the species ranked from most tolerant to most sensitive. More research is needed to identify which, if any, of the putatively identified active allelopathic compounds are responsible for the reductions we observe and to then derive a mechanistic understanding of how they influence germination and seedling vigor. However, we clearly show that Russian knapweed extract has bioherbicidal potential. Moreover, the differential sensitivity we observe between weedy and cultivated species, implies Russian knapweed extract could be used within an agricultural setting to reduce weed germination and establishment without significant damage to the sympatric cultivated species.

Acknowledgments

The authors would like to thank the Faculty of Agricultural Sciences and Technology, University of Mohaghegh Ardabili, Ardabil, Iran, and Rothamsted Research, Harpenden, Hertfordshire, UK for their support.

Author contributions

Conceptualization: Fatemeh Ahmadnia, Dana R. MacGregor
Formal analysis: Elham Samadi Kalkhoran, Dana R. MacGregor
Funding acquisition: Mohammad Taghi Alebrahim, Dana R. MacGregor
Investigation: Fatemeh Ahmadnia, Mohammad Taghi Alebrahim
Methodology: Fatemeh Ahmadnia, Elham Samadi Kalkhoran, Dana R. MacGregor
Project administration: Mohammad Taghi Alebrahim
Supervision: Mohammad Taghi Alebrahim
Validation: Elham Samadi Kalkhoran, Dana R. MacGregor
Writing–original draft: Fatemeh Ahmadnia, Elham Samadi Kalkhoran,
Writing–review & editing: Dana R. MacGregor

References

1. Okey-Onyesolu C, Hassanisaadi M, Bilal M, Barani M, Rahdar A, Iqbal J, Kyzas GZ. Nanomaterials as Nanofertilizers and Nanopesticides: An Overview. ChemistrySelect. 2021;6(33): 8645-8663. https://doi.org/10.1002/slct.202102379
2. Alebrahim MT, Zangoueinejad R., Tseng TM. Biochemical and molecular knowledge about developing herbicide-resistant weeds. In:Herbicide resistance in weeds and crops. 2017;101-132. https://doi.org/10.5772/intechopen.69211
3. Alebrahim MT, Samadi-Kalkhoran E, Tseng TMP. Joint action of herbicides on weeds and their risk assessment on earthworm (*Eisenia fetida* L.). In: New insights in herbicide science. 2022b; IntechOpen. https://doi.org/10.5772/intechopen.105462.
4. Samadi-Kalkhoran E, Alebrahim MT, Mohammaddoust Chamn Abad HR, Streibig JC, Ghavidel A, Tseng TMP. The survival response of earthworm (*Eisenia fetida* L.) to individual and binary mixtures of herbicides. Toxics. 2022;10(6):320. https://doi.org/10.3390/toxics10060320
5. Saif S, Tahir A, Chen Y. Green Synthesis of Iron Nanoparticles and Their Environmental Applications and Implications. Nanomaterials. 2016;6(11): 209: 1-26. https://doi.org/10.3390/nano6110209

6. Sharma AR, Satsangi S. Potential allelopathic influence of sunflower (*Helianthus anuus* L.) on germination and growth be haviour of two weeds in- vitro conditions. Int J Biotech Bioeng Res. 2013;4(5): 421-426.
7. Hickman DT, Comont D, Rasmussen A, Birkett MA. Novel and holistic approaches are required to realize allelopathic potential for weed management. Ecol and Evol. 2023;13: e10018. https://doi.org/10.1002/ece3.10018
8. Aci MM, Sidari R, Araniti F, Lupini A. Emerging trends in allelopathy: A Genetic Perspective for Sustainable Agriculture. Agronomy. 2022;12: 2043. https://doi.org/10.3390/ agronomy12092043
9. Rice EL. Allelopathy. 1984. 2nd ed.; Academic Press: Orlando, FL, USA, 400.p.
10. Einhellig F, Putnam A, Tang C. The science of allelopathy. New York, NY: Wiley. 1986;71-188
11. Qasem J. Allelopathy in crop-weed interaction field studies. Allelopathy J. 2012;30(2):159-176.
12. Bonamoni G, Ippolito F, Sematore M, Cesarano G, Incerti G, Saracino A. Water extracts of charred litter cause opposite effects on growth of plants and fungi. Soil Biol and Biochem. 2016;92:133-141. https://doi.org/10.1016/j.soilbio.2015.10.003
13. Desai N, Dethe U, Gaikwad D. Allelopathic effect of *Excoecaria agallocha* L. mangrove leaf leachate on germination and growth behavior of *Eleusine coracana* (Finger Millet). American J Plant Physiol. 2017;12, 38-44. https://doi.org/10.3923/ajpp.2017.38.44.
14. Rizvi SJH, Haque H, Singh VK, Rizvi V. A discipline called allelopathy. Allelopathy: basic and applied aspects. 1992;1-10. https://doi.org/10.1007/978-94-011-2376-1_1
15. Harper JR, Balke NE. Characterization of the inhibition of K+ absorption in oat roots by salicylic acid. Plant Physiol. 1981;68(6): 1349-1353. https://doi.org/10.1104/pp.68.6.1349
16. Einhellig FA, Rasmussen JA. Effects of three phenolic acids on chlorophyll content and growth of soybean and grain sorghum seedlings. J of Chem Ecol. 1979;5: 815-824. https://doi.org/10.1007/BF00986566.
17. Kruse M, Strandberg M, Strandberg, B. Ecological effects of allelopathic plants-a review. NERI technical report. 2000; 315.
18. Olivia M, Tatenda RJ, Stanford M, Ronald M. Study of pre and post early emergence allelopathic effects on selected weeds of several sorghum (*Sorghum bicolor* (L) Moench) and Pearl millet (*Pennisentum glaucuum* L.) cultivars in Zimbabwe. African J Agri Res. 2019;25(1):1–15. https://doi.org/10.28941/25-1(2019)-1
19. Hickman DT, Rasmussen A, Ritz K, Birkett MA. Neve P. Review: Allelochemicals as multi-kingdom plant defence compounds: towards an integrated approach. Pest Manag Sci. 2021;77: 1121-1131. https://doi.org/10.1002/ps.6076
20. Fletcher RA, Renney AJ. A growth inhibitor found in *centaurea* spp. Canadian J of Plant Sci. 1963;43: 475-481. https://doi.org/10.4141/cjps63-098
21. Quintana N, Weir TL, Du J, Brockling CD, Rieder JP, Stermitz FR, Pasckke MW, Vivanco JM. Phytotoxic polyacetylenes from roots of Russian knapweed (*Acroptilon repens* L.). Phytochemistry. 2008;69(14): 2572–2578. https://doi.org/10.1016/j.phytochem.2008.07.015
22. Alebrahim MT, Rouhi H, Serajchi M, Majd R, Ghorbani R. Study of dormancy-breaking and optimum temperature for germination of Russian knapweed (*Acroptilon repens* L.). Int J of Agri. 2011;1(1):19-25.

23. Alebrahim MT, Azadbakht A, Jafarzadeh Gallo P. Study on the effect of Russian knapweed (*Acroptilon repens* L.) aqueous extract on seed germination and growth characteristics of purslane, common yellow mallow and wheat. Iran J Seed Sci Res. 2018; 5(4):13–24. https://doi.org/10.22124/jms.2018.2942
24. Nabati Souha L, Alebrahim MT, Zangoeinejad R, Samadi-Kalkhoran E. Evaluation of the effect of Russian knapweed (*Acroptilon repens* L.) water extract on the control of some weeds in wheat (*Triticum aestivum* L.) formulated and pure as a biological herbicide. MSc thesis, Mohaghegh Ardabili University, Ardabil, Iran; 2021. 226 p.
25. Alebrahim MT, Hajjzadeh A, Ahmadnia F, Nabati Soha L. Assessing the different effect of aqueous and formulated extracts of Russian knapweed (*Acroptilon repens* L.) with chitosan on the germination of rye weed (*Secale cereale* L.). J Seed Res. 2022a;12(4):39–38. https://doi.org/10.30495/jsr.2023.1994021.1262
26. Nabati Souha L, Alebrahim MT, Habibi Yangjeh A, Feizpoor S. Green synthesis of iron oxide nanoparticles (Fe_3O_4) by extract of aerial organs of Russian knapweed (*Acroptilon repens* L.). Cell Mol Res (Iran J Biol). 2022;35(4):640–654. https://doi.org/10.1001.1.23832738.1401.35.4.7.7
27. Alebrahim MT, Fakhari R, Sharifi K. Allelopathic effect of *Acroptilon repens* extract on emergence of some crops and weeds. J Seed Res. 2016;20(6):21–13.
28. Eskandari Lombar M, Ebadi A, Alebrahim MT, Kheirizadeh Y. Effectiveness of some characteristics of Chenopodium album with foliar spraying of aqueous extract of *Acroptilon repens* as a bioherbicide. 1th International and 4th National Organic and Conventional Agriculture Congress. Mohaghegh Ardabili University. 2017.
29. Hatami Hampa A, Javanmard A, Alebrahim MT, Sofalian O. Allelopathic effects of aqueous extracts of Sorghum bicolor and *Acroptilon repens* on seedling growth and activity of antioxidant enzymes in wheat, sugar beet, *Chenopodium album* and *Amaranthus retroflexus*. J Plant Prot. 2018;32(1):101–119. https://doi.org/10.22067/JPP.V32I1.62909
30. Ghitasi I, Nikbakht M, Sadeghi H, Sabzali V, Sabzali S, Shahrani M. The hypoglycemic effects of a hydro-alcoholic extract from *Securigera securidaca* seeds on induced diabetic in male rats. Shahrekord Univ Med Sci J. 2007;8(4):68–73. https://sid.ir/paper/58693/en
31. Hoodgar F, Nasri S, Amin G. Investigation of antinociceptive and anti-inflammatory effects of hydro-alcoholic extract of *Securigera securidaca* L. Intern Med Today. 2011;17(1):12–9. http://imtj.gmu.ac.ir/article-1-1077-en.html
32. Afsharnezhad M, Shahangian SS, Rasti B, Ghasemi MF. Inhibitory potential of *Acroptilon repens* against key enzymes involved in Alzheimer and diabetes, phytochemical profile, radical scavenging, and antibacterial activity. Iran Biomed J. 2021;25(1):21–32. https://doi.org/10.29252/ibj.25.1.21
33. Akhgari Z, Tanomand A, Nazari R, Zargar M. Biological and antibacterial features of *Acroptilon repens* (L.) DC. Cres J Med Biol Sci. 2022;9(2):116–122. https://doi.org/10.34172/CJMB.2022.20
34. Watson AK. The biology of Canadian weeds: 43. *Acroptilon* (*Centaurea*) *repens* (L.) DC. Can J Plant Sci. 1980;60:993–1004. https://doi.org/10.4141/cjps80-142
35. Bai X, Tang J, Safaralizadeh R, Feizi MAH, Delirezh N, Zangeneh A, Abbasi N, Zanganeh MM. Green fabrication of bioactive copper nanoparticles using *Acroptilon repens* extract: An enhanced anti-lung cancer activity. Inorg Chem Commun. 2024;164:112393. https://doi.org/10.1016/j.inoche.2024.112393
36. Jacobs J, Denny K. Ecology and management of Russian knapweed [*Acroptilon repens* (L.) DC]. USDA NRCS Invasive Species Tech Note No. MT-7; 2006. https://www.nrcs.usda.gov/plantmaterials/mtpmctn13104.pdf
37. Robles M, Wang N, Kim R, Choi BH. Cytotoxic effects of repin, a principal sesquiterpene lactone of Russian knapweed. J Neurosci Res. 1997;47:90–97. https://doi.org/10.1002/(SICI)1097-4547(19970101)47:1<90::AID-JNR10>3.0.CO;2-E

38. Stevens KL, Riopelle RJ, Wong RY. Repin, a sesquiterpene lactone from *Acroptilon repens* possessing exceptional biological activity. J Nat Prod. 1990;53:218–221. https://doi.org/10.1021/np50067a038
39. Zhan ZJ, Hou XR. Sesquiterpenoid alkaloid from *Acroptilon repens*. Nat Prod Res. 2008;22(3):222–226. https://doi.org/10.1080/14786410701389159
40. Heap I. The International herbicide-resistant weed database. WeedScience.org; 2024. Available from: https://www.weedscience.org
41. Kumar S, Suleski M, Craig JE, Kasprowicz AE, Sanderford M, Li M, Stecher G, Hedges SB. TimeTree 5: An expanded resource for species divergence times. Mol Biol Evol. 2022;39(8):msac174. https://doi.org/10.1093/molbev/msac174
42. Bajwa AA, Akhter MJ, Iqbal N, Peerzada AM, Hanif Z, Manalil S, et al. Biology and management of *Avena fatua* and *Avena ludoviciana*: two noxious weed species of agro-ecosystems. Environ Sci Pollut Res Int. 2017;24(24):19465–19479. https://doi.org/10.1007/s11356-017-9810-y
43. Burger JC, Lee S, Ellstrand NC. Origin and genetic structure of feral rye in the western United States. Mol Ecol. 2006;15:2527–2539. https://doi.org/10.1111/j.1365-294X.2006.02938.x
44. Brunharo CACG, Gast R, Kumar V, Mallory-Smith CA, Tidemann BD, Beckie HJ. Western United States and Canada perspective: are herbicide-resistant crops the solution to herbicide-resistant weeds? Weed Sci. 2022;70:272–286. https://doi.org/10.1017/wsc.2022.6
45. Ahmadnia F, Ebadi A, Hashemi M, Ghavidel A. Investigating the short time effect of cover crops on physical and biological properties of soil. J Water Soil Conserv. 2020;26(6):277–290. https://doi.org/10.22069/jwsc.2019.16172.3145
46. Ahmadnia F, Ebadi A, Hashemi M, Nabati L. Investigating the effectiveness of Sunn hemp (*Crotalaria juncea*) and rye (*Secale cereale* L.) in weed suppression and yield of kohlrabi (Brassica oleracea var. gongylodes). J Agric Sci Sustain Prod. 2021;31(2):43–56. https://doi.org/10.22034/saps.2021.13090
47. Ahmadnia F, Ebadi A, Gudarzi M. Interaction effect of cover crop residues and hand weeding of weeds on iceberg lettuce (Lactuca sativa var. iceberg) yield. J Crop Ecophysiol. 2022;15(4):555–574. https://doi.org/10.30495/jcep.2022.689806
48. Camargo Silva G, Bagavathiannan M. Mechanisms of weed suppression by cereal rye cover crop: A review. Agron J. 2023;115:1571–1585. https://doi.org/10.1002/agj2.21347
49. Rebong D, Henriquez Inoa S, Moore VM, Reberg-Horton C, Mirsky S, Murphy JP, Leon RG. Breeding allelopathy in cereal rye for weed suppression. Weed Sci. 2023;72(1):30–40. https://doi.org/10.1017/wsc.2023.64
50. Shahvand B, Miri HR, Bagheri AR. Effect of different treatments on dormancy breaking of wild oat (*Avena fatua*). Int J Biosci. 2015;6(6):61–67. https://doi.org/10.12692/ijb/6.6.61-67
51. Şin B, Kadıoğlu I. A study on germination biology of wild mustard (Sinapis arvensis L.). Turk J Agric Food Sci Technol. 2021;9(4):728–732. https://doi.org/10.24925/turjaf.v9i4.728-732.4054
52. Ahmadnia F, Mokhtari AM. Allelopathic effects of Sunn Hemp (*Crotalaria juncea*): A review. 2023. In: 3th International and 7th National Conferance Organic and Conventional Agriculture. Ardabil, Iran; https://civilica.com/doc/1775754
53. Hussain MI, El-Sheikh MA, Reigosa MJ. Allelopathic potential of aqueous extract from *Acacia melanoxylon* R. Br. on *Lactuca sativa*. Plants. 2020;9(9):1228. https://doi.org/10.3390/plants9091228
54. Ahmadnia F, Ebadi A, Alebrahim MT, Parmoon G, Feizpoor S, Hashemi M. The efficacy of Sunn Hemp (*Crotalaria juncea*) and Fe_3O_4 nanoparticles in controlling weed seed germination. Agronomy. 2025;15:795. https://doi.org/10.3390/agronomy15040795
55. Trusheva B, Trusnkova D, Bankova V. Different extraction methods of biologically active components from propolis: A preliminary study. Chem Cent J. 2007;1:13. https://doi.org/10.1186/1752-153X-1-13
56. Perry D. Methodology and application of vigour tests. In: Handbook of vigour test methods. 1981.

57. Ahmadnia F, Alebrahim MT, Nabati Souha L, MacGregor DR. Evaluation of techniques to break seed dormancy in Redroot pigweed (*Amaranthus retroflexus*). Food Sci Nutr. 2024;12(4):2334–2345. https://doi.org/10.1002/fsn3.3920
58. Scott SJ, Jones RA, Williams WA. Review of data analysis methods for seed germination. Crop Sci. 1984;24(6):1192–1199. https://doi.org/10.2135/cropsci1984.0011183X002400060043x
59. Burnett SE, Pennisi SV, Thomas PA, van Iersel MW. Controlled drought affects morphology and anatomy of *Salvia splendens*. J Am Soc Hortic Sci. 2005;130(5):775–781. https://doi.org/10.21273/JASHS.130.5.775
60. MacGregor DR, Kendall SL, Florance H, Fedi F, Moore K, Paszkiewicz K, Penfield S. Seed production temperature regulation of primary dormancy occurs through control of seed coat phenylpropanoid metabolism. New Phytol. 2015;205(2):642–652. https://doi.org/10.1111/nph.13090
61. Maguire JD. Speed of germination—aids in selection and evaluation for seedling emergence and vigor. Crop Sci. 1962;2:176–177. https://doi.org/10.2135/cropsci1962.0011183X000200020033x
62. Tekrony DM, Egli DB. Relationship of seed vigor to crop yield: A review. Crop Sci. 1991;31(3):816–822. https://doi.org/10.2135/cropsci1991.0011183X003100030054x
63. Saeb H, Khayyat M, Zarezadeh A, Moradinezhad F, Samadzadeh A, Safaee M. Effects of NaCl stress on seed germination attributes of periwinkle (*Catharanthus roseus* L.) and corn poppy (*Papaver rhoeas* L.) plants. Plant Breed Seed Sci. 2013;67:115–123. https://doi.org/10.2478/v10129-011-0074-2
64. Czabator FJ. Germination value: An index combining speed and completeness of pine seed germination. For Sci. 1962;8:386–396. https://doi.org/10.1093/forestscience/8.4.386
65. Ellis RH. Seed and seedling vigor in relation to crop growth and yield. Plant Growth Regul. 1992;11:249–255. https://doi.org/10.1007/BF00024563
66. Covarrubias-Cárdenas AG, Martínez-Castillo JI, Medina-Torres N, Ayora-Talavera T, Espinosa-Andrews H, García-Cruz NU, Pacheco N. Antioxidant capacity and UPLC-PDA ESI-MS phenolic profile of *Stevia rebaudiana* dry powder extracts obtained by ultrasound assisted extraction. Agronomy. 2018;8(9):170. https://doi.org/10.3390/agronomy8090170
67. Sirdaarta J, Matthews B, White A, Cock IE. GC-MS and LC-MS analysis of Kakadu plum fruit extracts displaying inhibitory activity against microbial triggers of multiple sclerosis. Pharmacogn Commun. 2015;5(2):100–115. https://doi.org/10.5530/pc.2015.2.2
68. Wright MH, Sirdaarta J, White A, Greene AC, Cock IE. GC-MS headspace analysis of *Terminalia ferdinandiana* fruit and leaf extracts which inhibit Bacillus anthracis growth. Pharmacogn Commun. 2017;9(1):73–82. https://doi.org/10.5530/pj.2017.1.14
69. Mehdizadeh M, Alebrahim MT, Roushani M, Streibig JC. Evaluation of four different crops' sensitivity to sulfosulfuron and tribenuron methyl soil residues. Acta Agric Scand B Soil Plant Sci. 2016;66(8):706–713. https://doi.org/10.1080/09064710.2016.1212919
70. Mehdizadeh M, Alebrahim MT, Roushani M. Determination of two sulfonylurea herbicides residues in soil environment using HPLC and phytotoxicity of these herbicides by lentil bioassay. Bull Environ Contam Toxicol. 2017;99:93–99. https://doi.org/10.1007/s00128-017-2076-8
71. Tahmasebi BK, Alebrahim MT, Roldán-Gómez RA, da Silveira HM, de Carvalho LB, Alcántara-de la Cruz R, De Prado R. Effectiveness of alternative herbicides on three Conyza species from Europe with and without glyphosate resistance. Crop Prot. 2018;112:350–355. https://doi.org/10.1016/j.cropro.2018.06.021
72. Hicks HL, Comont D, Coutts SR, Crook L, Hull R, Norris K, Neve P, Childs DZ, Freckleton RP. The factors driving evolved herbicide resistance at a national scale. Nat Ecol Evol. 2018;2:529–536. https://doi.org/10.1038/s41559-018-0470-1
73. Kanatas PJ, Travlos IS, Gazoulis J, Antonopoulos N, Tsekoura A, Tataridas A, Zannopoulos S. The combined effects of false seedbed technique, post-emergence chemical control and cultivar on weed management and yield of barley in Greece. Phytoparasitica. 2020;48(3):131–143. https://doi.org/10.1007/s12600-020-00783-x

74. Dayan FE, Duke SO. Natural compounds as next-generation herbicides. Plant Physiol. 2014;166:1090–1105. https://doi.org/10.1104/pp.114.239061
75. Hasan M, Ahmad-Hamdani MS, Rosli AM, Hamdan H. Bioherbicides: An eco-friendly tool for sustainable weed management. Plants. 2021;10(6):1212. https://doi.org/10.3390/plants10061212
76. Bailey KL. The bioherbicide approach to weed control using plant pathogens. Integr Pest Manag. 2014; 245–266. https://doi.org/10.1016/B978-0-12-398529-3.00014-2
77. Miranda MP, Fitches EC, Sukiran NA, Eduardo WI, Garcia RB, Jaciani FJ, Readshaw JJ, Bell J, Peña L. Spider venom neurotoxin based bioinsecticides: A novel bioactive for the control of the Asian citrus psyllid *Diaphorina citri* (Hemiptera). Toxicon. 2024;239:107616. https://doi.org/10.1016/j.toxicon.2024.107616
78. Cenobio-Galindo AJ, Hernández-Fuentes AD, González-Lemus U, Zaldívar-Ortega AK, González-Montiel L, Madariaga-Navarrete A, Hernández-Soto I. Biofungicides based on plant extracts: On the road to organic farming. Int J Mol Sci. 2024;25(13):6879. https://doi.org/10.3390/ijms25136879
79. Duke SO, Pan Z, Bajsa-Hirschel J, Boyette CD. The potential future roles of natural compounds and microbial bioherbicides in weed management in crops. Adv Weed Sci. 2022;40(1):e020210054. https://doi.org/10.51694/AdvWeedSci/2022;40.
80. Scavo A, Mauromicale G. Crop allelopathy for sustainable weed management in agroecosystems: Knowing the present with a view to the future. Agronomy. 2021;11(11):2104. https://doi.org/10.3390/agronomy11112104
81. Soltys D, Krasuska U, Bogatek R, Gniazdowska A. Allelochemicals as bioherbicides: Present and perspectives. Herbic Curr Res Case Stud Use. 2013; IntechOpen. https://doi.org/10.5772/56185
82. Ahmadnia F, Ebadi A, Hashemi M, Ghavidel A, Alebrahim MT. Investigating the effect of aqueous extracts of sunn hemp (*Crotalaria juncea*) and oats (*Avena sativa* L.) on the germination of wild mustard weed (*Sinapis arvensis*). Iran J Seed Sci Res. 2023;10(2):1–19. https://doi.org/10.22124/jms.2023.7605
83. Alshahrani TS, Suansa NI. Application of biochar to alleviate effects of allelopathic chemicals on seed germination and seedling growth. BioResources. 2020;15(1):382–400. https://doi.org/10.15376/biores.15.1.382-400
84. Hosseini M, Mojab M, Samadi-Kalkhoran E, Zamani G, Alebrahim MT. The allelopathic effects of extract and wheat residue rates on prostrate pigweed (*Amaranthus bilituides*) and common lambsquarters (*Chenopodium album*). Plant Prod Genet. 2022;3(2):261–274. https://doi.org/10.34785/J020.2022.005
85. Safahani AR, Ghooshchi F. Allelopathic effects of aqueous and residue of different weeds on germination and seedling growth of wheat. J Plant Res Iran J Biol. 2014;27(1,1):100–109. https://doi.org/20.1001.1.23832592.1393.27.1.11.9
86. Alebrahim MT, Hajzadeh A, Ahmadnia F, Farzaneh S. Investigating the effect of pure and nonencapsulated extract of Russian knapweed (*Acroptilon repens* L.) with chitosan on the germination of redroot pigweed (*Amaranthus retroflexus*). Plant Prod Genet. 2023; Available Online from 05 December. https://doi.org/10.22034/PLANT.2023.62944
87. Pannacci E, Masi M, Farneselli M, Tei F. Evaluation of mugwort (*Artemisia vulgaris* L.) aqueous extract as a potential bioherbicide to control *Amaranthus retroflexus* L. in maize. Agriculture. 2020;10(12):642. https://doi.org/10.3390/agriculture10120642
88. Da Silva UP, Furlani GM, Demuner AJ, da Silva OLM, Varejão EVV. Allelopathic activity and chemical constituents of extracts from roots of *Euphorbia heterophylla* L. Nat Prod Res. 2019;33(18):2681–2684. https://doi.org/10.1080/14786419.2018.1460829
89. Bakrim S, Benkhaira N, Bourais I, Benali T, Lee LH, Omari NE, Sheikh RA, Goh KW, Ming LC, Bouyahya A. Health benefits and pharmacological properties of stigmasterol. Antioxidants. 2022;11(10):1912. https://doi.org/10.3390/antiox11101912
90. Koc J, Wasilewski J, Androsiuk P, Kellmann-Sopyla W, Chwedorzewska KJ, Gielwanowska I. The effects of methanesulfonic acid on seed germination and morphophysiological changes in the

seedlings of two Colobanthus species. Acta Soc Bot Pol. 2018;87(4):3601. https://doi.org/10.5586/asbp.3601

91. Sadeghi S, Rahnavard A, Ashrafi ZY. Allelopathic effect of *Helianthus annuus* (sunflower) on Solanum nigrum (black nightshade) seed germination and growth in laboratory condition. J Hortic Sci Ornamental Plants. 2010;2(1):32–37
92. Feng C, Yongjie M, Haiwei S, Xiaofeng L, Wenguan Z, Jianwei L, Wenyu Y, Kai S. Effect of plant allelochemicals on seed germination and its ecological significance. Chin J Eco-Agric. 2017;25(1):36–46. https://doi.org/10.13930/j.cnki.cjea.160632
93. Burgos NR, Talbert RE. Differential activity of allelochemicals from Secale cereale in seedling bioassays. Weed Sci. 2000;48(3):302–310. https://doi.org/10.1614/0043-1745(2000)048[0302:DAOAFS]2.0.CO;2
94. Jafariehyazdi E, Javidfar F. Comparison of allelopathic effects of some Brassica species in two growth stages on germination and growth of sunflower. Plant Soil Environ. 2011;57(2):52–56. https://doi.org/10.17221/139/2010-PSE
95. Tokasi S, Rashed Mohasel M, Bannayan Aval M. Allelopathic potential of alfalfa shoot aqueous extract on germination and seedling growth of four weed species. Iran J Field Crops Res. 2011;9(1):60–69. https://doi.org/10.22067/GSC.V9I1.10495
96. Jmii G, Khadhri A, Haouala R. Thapsia garganica allelopathic potentialities explored for lettuce growth enhancement and associated weed control. Sci Hortic. 2020;262:109068. https://doi.org/10.1016/j.scienta.2019.109068
97. Li Y, Li X, Li Q, Yue M. Aqueous extract of fallen Indian soapberry (*Sapindus mukorossi*) leaves decreased seed germination but increased seedling growth of four vegetables. Appl Ecol Environ Res. 2021;19(1):331–347. http://dx.doi.org/10.15666/aeer/1901_331347
98. Nikolova M, Traykova B, Yankova-Tsvetkova E, Stefanova T, Dzhurmanski A, Aneva I, Berkov S. Herbicide potential of selected essential oils from plants of Lamiaceae and Asteraceae families. Acta Agrobot. 2021;74(1):1–7. https://doi.org/10.5586/aa.7411
99. Tutenocakli T, Coskun Y, Tas I, Oral A, Turker G. Allelopathic effects of some essential oil components on germination and seedling growth of wheat. Curr Trends Nat Sci. 2022;11(21):513–520. https://doi.org/10.47068/ctns.2022.v11i21.055
100. Zhao L, Wang J, Zhang H, Wang P, Wang C, Zhou Y, Li H, Yu S, Wu R. Inhibitory effect of carvacrol against *Alternaria alternata* causing goji fruit rot by disrupting the integrity and composition of cell wall. Front Microbiol. 2023;14:1139749. https://doi.org/10.3389/fmicb.2023.1139749
101. Verdeguer M, Torres-Pagan N, Muñoz M, Jouini A, García-Plasencia S, Chinchilla P, Merbegal M, Salamone A, Agnello S, Carrubba A, Cabeiras-Freijanes L, Regueira-Marcos L, Sánchez-Moreiras AM, Blázquez MA. Herbicidal activity of Thymbra capitata (L.) Cav. essential oil. Molecules. 2020;25(12):2832. https://doi.org/10.3390/molecules25122832

Fig 1. The effect of Russian Knapweed extract on percent germination (a), germination rate (b), mean germination time (c), radicle length (d), and plumule length (e). Data are shown as averages ± standard deviation of 20 plants observed for 14 days as described in the methods. For c, d and e, the data are presented as relative to the control values

Fig 2. Pearson's correlation coefficients (n = 198) among eleven quantitative traits on *Amaranthus retroflexus*, *Secale cereal*, *Avena fatua*, *Sinapis arvensis*, *Triticum aestivum*, and *Vicia faba*. The correlation coefficients were significant at the statistical probability level of 1 and 5%. GP, germination percentage; GR, germination rate; MGT, Mean germination time; MDG, Mean daily germination; SPL, Seedling plumule length; SRL, Seedling radicle length; GI, Germination index; PV, Peak value; GV, Germination value.

Figure

Click here to access/download;Figure;Figures 1 2.docx

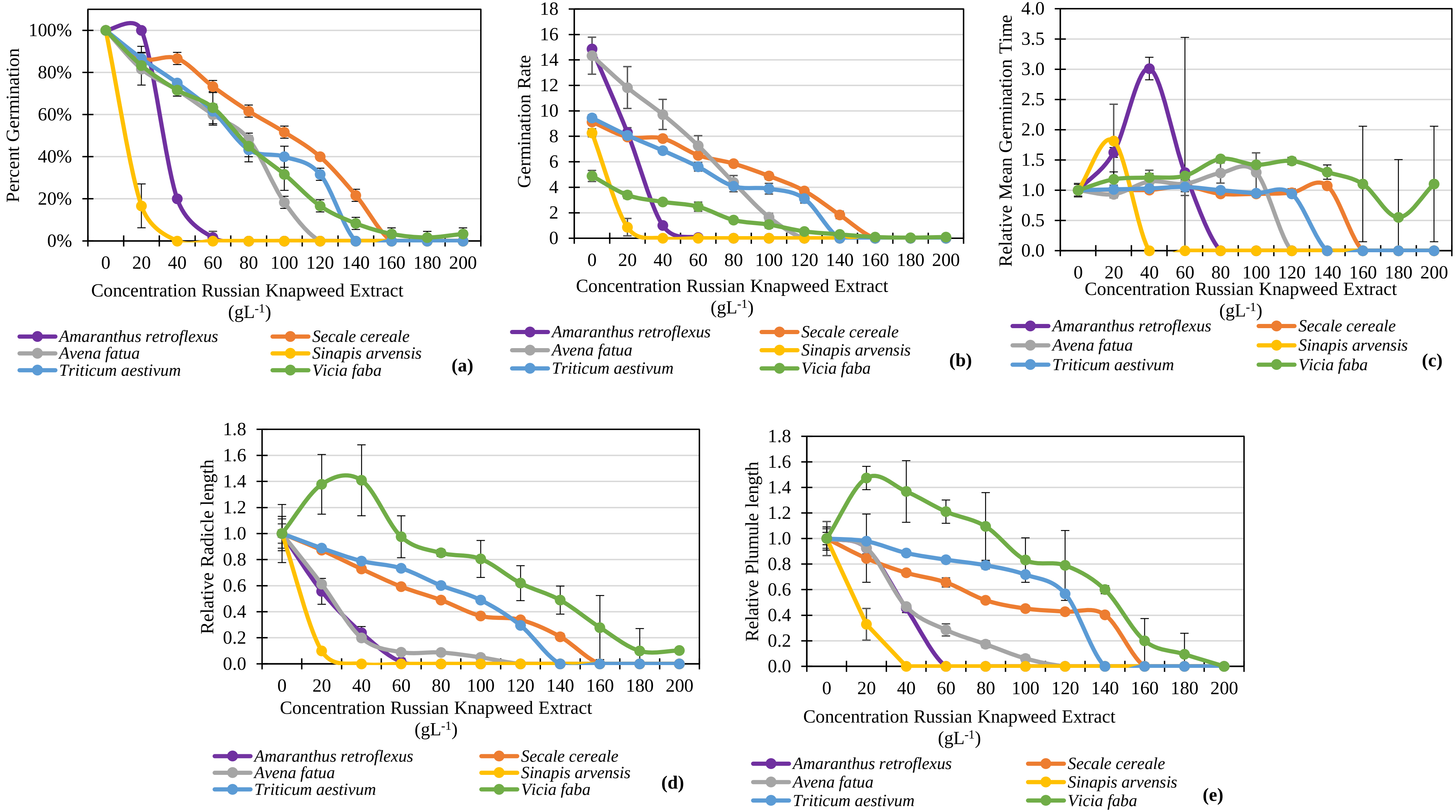

Fig 1. The effect of Russian Knapweed extract on percent germination (a), germination rate (b), mean germination time (c), radicle length (d), and plumule length (e). Data are shown as averages ± standard deviation of 20 plants observed for 14 days as described in the methods. For c, d and e, the data are presented as relative to the control values.

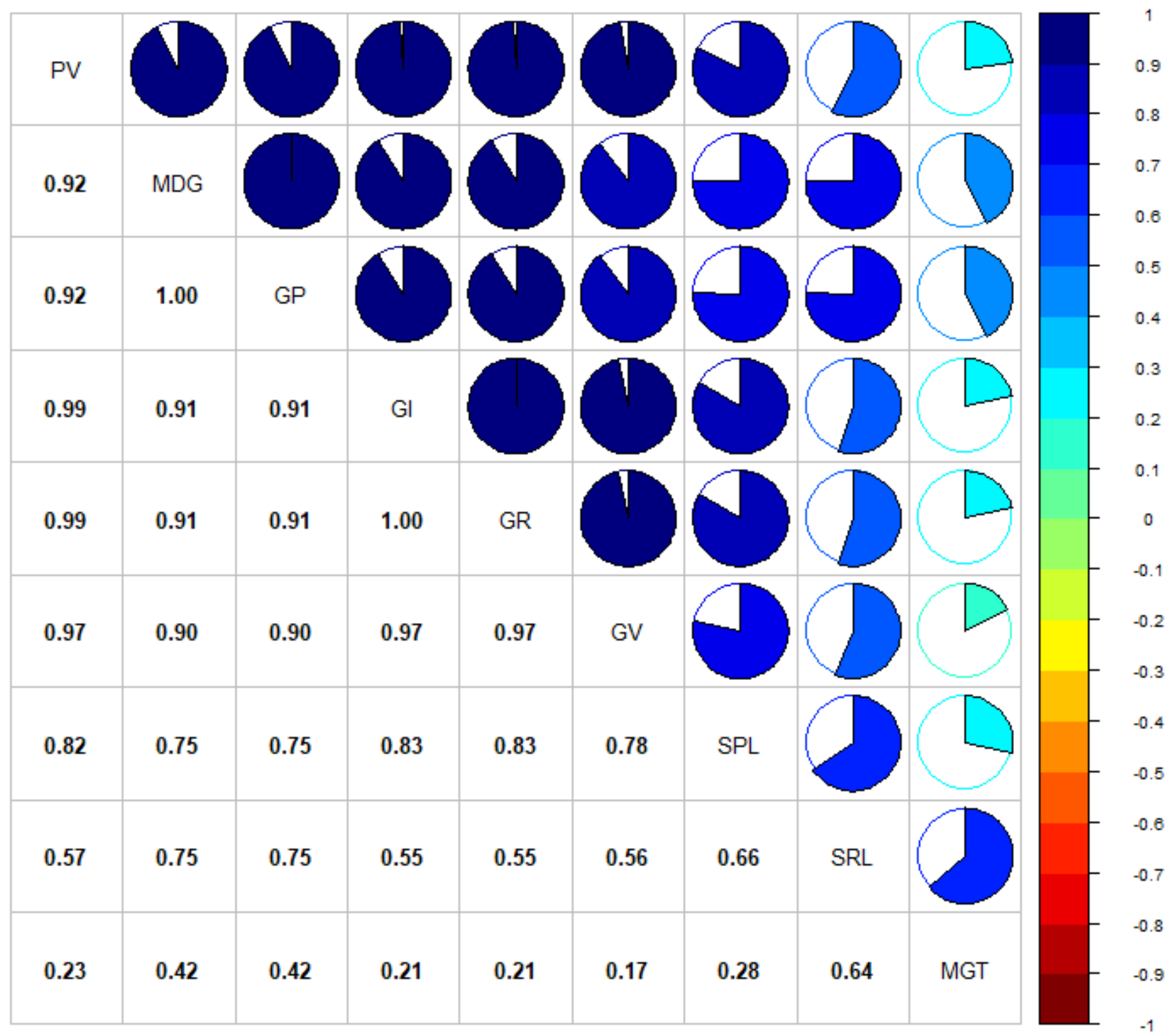

Fig 2 Pearson's correlation coefficients (n = 198) among eleven quantitative traits on *Amaranthus retroflexus*, *Secale cereal*, *Avena fatua*, *Sinapis arvensis*, *Triticum aestivum*, and *Vicia faba*. The correlation coefficients were significant at the statistical probability level of 1 and 5%. GP, germination percentage; GR, germination rate; MGT, Mean germination time; MDG, Mean daily germination; SPL, Seedling plumule length; SRL, Seedling radicle length; GI, Germination index; PV, Peak value; GV, Germination value.
