# Supplementary material for: Under controlled conditions, lower concentrations of aqueous extracts of Acroptilon repens are needed to inhibit seed germination and seedling growth of weeds than crops
Source: PLoS One. 2026 Aug 27;21(8):e0354454. doi: 10.1371/journal.pone.0354454 (PMC13521396; doi:10.1371/journal.pone.0354454)
Supplement: Supplementary file 2 [file pone.0354454.s007.docx]

**Dear Editor and Authors,**

I have many concerns about this research. In my opinion, the manuscript was written below the standards of the journal PLOS ONE. The research is at a preliminary stage, with inadequately chosen parameters and highly questionable results. Most parts of the abstract, introduction, material and methods, results, and conclusion must be rewritten. In this form, the text is very difficult to read. **ENGLISH LANGUAGE EDITING AND STYLE POLISHING FOR THIS MANUSCRIPT ARE NECESSARY.** In this form, my recommendation for this manuscript is **REJECT.** The authors must be making a **MAJOR REVISION** and resubmitting the manuscript (it is necessary to expand the effect of aqueous extract in the research on plants, to calculate and present the results in another way, etc).

**Comments:**

**Line 1:** Title: The title is not adequate; it is more of a declarative sentence. Please see below my proposal for the manuscript title:

**Allelopathic potential of aqueous extract of *Acroptilon repens* (Russian knapweed) for management of noxious weeds**

**Introduction**

**Lines 46-56:** Traditional management approaches frequently depend on chemical inputs, particularly herbicides, to mitigate the adverse impacts of weeds on agricultural output [1]. The overuse and indiscriminate application of these substances, however, results in herbicide resistance [2], significant environmental issues, such as the degradation of agricultural ecosystems, accumulation of chemical residues, and the contamination of vital resources like air, water, and soil [3,4]. Therefore, in line with environmental preservation and economic sustainability measures, less harmful but equally effective alternatives are needed for successful weed management strategies in agriculture production [5].

**Please replace with text below:**

**Traditional management approaches often rely on chemical inputs, particularly herbicides, to mitigate the adverse effects of weeds on agricultural yields [1]. The overuse and indiscriminate application of these substances result in herbicide resistance [2], as well as significant environmental issues, including the degradation of agricultural ecosystems, the accumulation of chemical residues, and the contamination of vital resources such as air, water, and soil [3,4]. Therefore, in line with environmental preservation and economic sustainability measures, less harmful but equally effective alternatives are needed for successful weed management strategies in agricultural production [5].**

**Lines 57-78:** This part of the introduction has basic information and a definition of allelopathy. It is well-known literature and need not be detailed in the introduction. Please delete this part, or write one or two sentences about this.

**Lines 79-117:** This part is written too extensively. A lot of unnecessary information is written. In contrast, the objectives of the work are unclear.

**Lines 117-120: This part is written like a conclusion, but an introduction should contain clear objectives of the research. In addition, it is impossible to draw such conclusions based on the results presented in the manuscript. Only a seed bioassay was performed here, which is the basis for preliminary research. After that, it is necessary to do a plant bioassay in controlled conditions and the field. This type of research can not give us clear answers about the effectiveness of plant extracts in controlling weed species and their herbicidal potential.**

**FINALLY, THE COMPLETE INTRODUCTION MUST BE REWRITTEN.**

**Material and methods**

The methods of work and the material used are written unreasonably and include too much unnecessary information (for example: “were obtained from the Seed and Plant Improvement Institute of Iran, introduced in 1991.” “were also obtained from the Seed and Plant Improvement Institute of Iran, where it was introduced in 2017.” “Both seeds were provided from the Moghan Research and Natural Resources Center a month before the experiment and stored in a cool, dark environment.”).

Also, I do not agree with all the selected measured parameters. In general, in addition to the percentage of germination, germination rate, and seedling length, other parameters are not of great importance for this type of research.

Decide whether to use Latin or common names. It is unnecessary to repeat both several times.

**Line 144:** “affected by infection”, - **What did you mean? Weeds may be present in a wheat field, but they are not an infection.**

You have a wide range of solution concentrations from 2% to 20%. It would be better to calculate the dose-response EC50 using R or another program. Correlation is not the best method for this type of data.

**Results**

The results section is impossible to follow. **Table 1** is overwhelming, with too many parameters that do not show any value or significance of the results. Each parameter is described in a separate subheading. Also, redundant and, for the most part, unreasonable. Not a good enough choice of statistics and presentation of results.

**Table 4**: Could you please explain the type of result you present here? You have a molecular formula, but you do not have the percentage content of the compound. In mass spectrometry, retention time, molecular ions [M-H]-, and MS2 fragments (specifically, Q1 and Q3 in a triple quadrupole system) are key parameters used for compound identification and quantification. But they are not a response to the presence of components in the plant extract.

**Line 397-405:** In this part of the results, we do not have any valuable information about the content of allelopathic compounds, and which one could be most effective for weed control.

The manuscript has about 40 pages. It is too much, too much unnecessary and repeated information.

**FINALLY, THE COMPLETE RESULTS MUST BE RECALCULATED, AND ALSO THE TEXT OF THIS SECTION AND THE DISCUSSION MUST BE REWRITTEN.**

**Literature**

In this manuscript, you listed 101 literature sources. That amount is usually suitable for a PhD thesis, but not for a research article. Please choose 20-30 of the most relevant references for your research.
