# Supplementary material for: Under controlled conditions, lower concentrations of aqueous extracts of Acroptilon repens are needed to inhibit seed germination and seedling growth of weeds than crops
Source: PLoS One. 2026 Aug 27;21(8):e0354454. doi: 10.1371/journal.pone.0354454 (PMC13521396; doi:10.1371/journal.pone.0354454)
Supplement: Supplementary file 3 [file pone.0354454.s010.docx]

**Additional Editor Comments:**

This is a good study comparing to similar studies. However, it needs to be well organized and rewritten. I think you presented more statistical analysis in your abstract. I recommend using passive sentences are better in our area. Title can be "Control potential of aqueous extract of *Acroptilon repens* (Russian knapweed) on some important weed species". Could you please carefully follow the reviewers' recommendations.

***Response:*** *We appreciate both the Editor’s and Reviewer 1’s comments on our title. We have generated a title that combines the best of both suggestions and emphasizes that we also studied the relevant crop species - “Allelopathic potential of Acroptilon repens (Russian knapweed) aqueous extract for managing agriculturally important weed species in comparison to relevant crops”.*

**Reviewer 1**

Dear Authors,

**General comment:** I have many concerns about this research. In my opinion, the manuscript was written below the standards of the journal PLOS ONE. The research is at a preliminary stage, with inadequately chosen parameters and highly questionable results. Most parts of the abstract, introduction, material and methods, results, and conclusion must be rewritten. In this form, the text is very difficult to read. ENGLISH LANGUAGE EDITING AND STYLE POLISHING FOR THIS MANUSCRIPT ARE NECESSARY. In this form, my recommendation for this manuscript is REJECT. The authors must be making a MAJOR REVISION and resubmitting the manuscript (it is necessary to expand the effect of aqueous extract in the research on plants, to calculate and present the results in another way, etc).

***General response:*** *We appreciate your valuable comments. We've addressed your questions and rewritten the paper.*

**Comments:**

Line 1: Title: The title is not adequate; it is more of a declarative sentence. Please see below my proposal for the manuscript title:

Allelopathic potential of aqueous extract of *Acroptilon repens* (Russian knapweed) for management of noxious weeds

***Response:*** *We have changed the title combining this suggestion with the one made by the Editor.*

**Introduction**
Lines 46-56: Traditional management approaches frequently depend on chemical inputs, particularly herbicides, to mitigate the adverse impacts of weeds on agricultural output [1]. The overuse and indiscriminate application of these substances, however, results in herbicide resistance [2], significant environmental issues, such as the degradation of agricultural ecosystems, accumulation of chemical residues, and the contamination of vital resources like air, water, and soil [3,4]. Therefore, in line with environmental preservation and economic sustainability measures, less harmful but equally effective alternatives are needed for successful weed management strategies in agriculture production [5].

***Response*:** *The text written by the referee was replaced.*

Lines 57-78: This part of the introduction has basic information and a definition of allelopathy. It is well-known literature and need not be detailed in the introduction. Please delete this part, or write one or two sentences about this.

***Response*:** *We removed the definitions related to allelopathy and rewrote the introduction.*

Lines 79-117: This part is written too extensively. A lot of unnecessary information is written. In contrast, the objectives of the work are unclear.

***Response*:** *The introduction has been completely rewritten.*

Lines 117-120: This part is written like a conclusion, but an introduction should contain clear objectives of the research. In addition, it is impossible to draw such conclusions based on the results presented in the manuscript. Only a seed bioassay was performed here, which is the basis for preliminary research. After that, it is necessary to do a plant bioassay in controlled conditions and the field. This type of research can not give us clear answers about the effectiveness of plant extracts in controlling weed species and their herbicidal potential.

***Response*:** *The introduction has been completely rewritten.*

FINALLY, THE COMPLETE INTRODUCTION MUST BE REWRITTEN.

***Response*:** *The introduction has been completely rewritten.*

**Material and methods**

The methods of work and the material used are written unreasonably and include too much unnecessary information (for example: “were obtained from the Seed and Plant Improvement Institute of Iran, introduced in 1991.” “were also obtained from the Seed and Plant Improvement Institute of Iran, where it was introduced in 2017.” “Both seeds were provided from the Moghan Research and Natural Resources Center a month before the experiment and stored in a cool, dark environment.”).

***Response:*** *We removed unnecessary sections based on the referee's opinion and rewrote the materials and methods.*

Also, I do not agree with all the selected measured parameters. In general, in addition to the percentage of germination, germination rate, and seedling length, other parameters are not of great importance for this type of research.

***Response:*** *Based on the Reviewer’s suggestion, we have moved the data pertaining to the other parameters to supplementary files – Figure 1 and the tables in the main paper now only deal with percentage of germination, germination rate, and seedling length. We kept the data concerning Mean Germination Time in the main paper as it was the one parameter unchanged by application of* A. repens *extract.*

Decide whether to use Latin or common names. It is unnecessary to repeat both several times.

***Response:*** *Common names were removed.*

Line 144: “affected by infection”, - What did you mean? Weeds may be present in a wheat field, but they are not an infection.

***Response:*** *By the word infection, we meant "the excessive and unwanted presence of A. repens weeds in a wheat field." The sentence was rewritten.*

You have a wide range of solution concentrations from 2% to 20%. It would be better to calculate the dose-response EC50 using R or another program. Correlation is not the best method for this type of data.

***Response*:** *We initially used R software to conduct dose-response (EC₅₀) analysis; however, the model fit was poor, so we did not proceed with this method for formal interpretation. The R output is provided in the appendix for transparency. Other dose-response tools were considered but found to be less reliable. Instead, we used ANOVA and regression analyses to evaluate germination responses across concentrations. Summary statistics and mean comparisons are presented in Table 1, while detailed outputs are available in the supplementary material.*

| Germination Percentage | | | | | | |
| --- | --- | --- | --- | --- | --- | --- |
| Type of plant | b | d | ED_10_ | ED_50_ | ED_90_ | Lack of fit |
| *Amaranthus retroflexus* | 8.81 (6.29) | 100.43 (3.25) | 26.64 (7.78) | 34.17 (3.97) | 43.84 (3.06) | <0.01 |
| *Secale cereal* | 4.29 (0.44) | 89.27 (2.38) | 61.68 (4.82) | 102.83 (3.38) | 171.46 (6.92) | <0.01 |
| *Avena Fatua* | 4.67 (0.64) | 85.95 (2.76) | 47.49 (4.63) | 75.99 (3.08) | 121.59 (5.53) | <0.01 |
| *Sinapis arvensis* | 7.83 (23.83) | 99.99 (3.71) | 12.30 (18.18) | 16.28 (10.18) | 21.59 (4.99) | <0.01 |
| *Triticum aestivum* | 3.10 (0.29) | 91.47 (2.90) | 38.69 (4.08) | 78.44 (3.66) | 159.04 (7.89) | <0.01 |
| *Vicia faba* | 3.14 (0.33) | 90.11 (2.94) | 38.05 (4.30) | 76.54 (3.62) | 153.94 (8.42) | <0.01 |
| Germination Rate | | | | | | |
| *Amaranthus retroflexus* | 4.20 (0.58) | 14.85 0.38) | 12.54 (0.96) | 21.16 (0.58) | 35.71 (2.81) | <0.01 |
| *Secale cereal* | 4.61 (0.58) | 8.04 (0.24) | 65.50 (5.84) | 105.40 (3.83) | 169.61 (7.60) | <0.01 |
| *Avena Fatua* | 3.52 (0.28) | 13.04 (0.31) | 32.53 (2.51) | 60.67 (2.08) | 113.18 (4.17) | <0.01 |
| *Sinapis arvensis* | 5.44 (13.77) | 8.27 (0.38) | 9.01 (18.20) | 13.50 (13.50) | 20.21 (1.88) | <0.01 |
| *Triticum aestivum* | 2.94 (0.31) | 8.60 (0.30) | 36.74 (4.57) | 77.49 (4.25) | 163.40 (9.12) | <0.01 |
| *Vicia faba* | 2.11 (0.52) | 4.49 (0.42) | 18.93 (7.99) | 53.45 (9.94) | 150.89 (22.41) | <0.01 |
| Germination Index | | | | | | |
| *Amaranthus retroflexus* | 4.20 (0.58) | 14.85 (0.38) | 12.54 (0.96) | 21.16 (0.58) | 35.71 (2.81) | <0.01 |
| *Secale cereal* | 4.61 (0.58) | 8.04 (0.24) | 65.50 (5.84) | 105.40 (3.83) | 169.61 (7.60) | <0.01 |
| *Avena Fatua* | 3.52 (0.28) | 13.04 (0.31) | 32.53 (2.51) | 60.67 (2.08) | 113.18 (4.17) | <0.01 |
| *Sinapis arvensis* | 5.44 (13.77) | 8.27 (0.38) | 9.01 (18.20) | 13.50 (13.50) | 20.21 (1.88) | <0.01 |
| *Triticum aestivum* | 2.94 (0.31) | 8.60 (0.30) | 36.74 (4.57) | 77.49 (4.25) | 163.40 (9.12) | <0.01 |
| *Vicia faba* | 2.11 (0.52) | 4.49 (0.42) | 18.93 (7.99) | 53.45 (9.94) | 150.89 (22.41) | <0.01 |
| Mean Germination Ttime | | | | | | |
| *Amaranthus retroflexus* | 30.74 (9.99) | 2.91 (0.34) | 57.77 (1.67) | 61.27 (1.70) | 60.49 (2.23) | 0.11 |
| *Secale cereal* | 109.14 (9.99) | 2.29 (0.21) | 149.05 (9.66) | 152.08 (9.85) | 155.17 (10.00) | 0.11 |
| *Avena Fatua* | 80.76 (9.99) | 2.02 (0.24) | 109.01 (9.70) | 111.78 (9.94) | 114.61 (10.20) | 0.11 |
| *Sinapis arvensis* | 30.68 (9.99) | 3.77 (0.42) | 30.73 (9.41) | 30.26 (9.98) | 34.63 (10.61) | 0.11 |
| *Triticum aestivum* | 90.75 (9.99) | 2.20 (0.24) | 120.57 (5.04) | 123.31 (5.16) | 126.12 (5.30) | 0.11 |
| *Vicia faba* | 0.12 (4.98) | 5.04 (0.18) | 1.19 *10^49^ (8.98*10^51^) | 9.55*10^56^ (10.00) | 7.62 *10^64^ (5.72*10^67^) | 0.11 |
| Plumule length | | | | | | |
| *Amaranthus retroflexus* | 31.22 (1295.44) | 3.24 (0.25) | 37.15 (113.87) | 39.85 (5.86) | 42.76 (118.57) | <0.01 |
| *Secale cereal* | 2.80 (0.43) | 6.21 (0.29) | 43.44 (7.96) | 95.09 (7.00) | 208.16 (17.00) | <0.01 |
| *Avena Fatua* | 2.87 (0.11) | 21.34 (0.32) | 18.80 (0.87) | 40.40 (0.92) | 86.82 (2.49) | <0.01 |
| *Sinapis arvensis* | 5.63 (5.00) | 7.50 (0.36) | 11.92 (5.51) | 17.61 (2.14) | 26.01 (6. 10) | <0.01 |
| *Triticum aestivum* | 28.05 (24.06) | 6.21 (0.15) | 113.40 (6.13) | 122.64 (2.05) | 132.63 (10.82) | <0.01 |
| *Vicia faba* | 5.24 (1.14) | 3.96 (0.19) | 82.65 (10.57) | 125.71 (6.62) | 191.19 (14.72) | <0.01 |
| Radicle length | | | | | | |
| *Amaranthus retroflexus* | 2.64 (2.05) | 2.08 (0.55) | 9.76 (8.76) | 22.40 (9.59) | 51.44 (32.28) | <0.01 |
| *Secale cereal* | 2.62 (0.43) | 7.88 (0.46) | 34.13 (7.41) | 78.83 (7.38) | 182.07 (17.93) | <0.01 |
| *Avena Fatua* | 2.47 (0.30) | 13.41 (0.54) | 9.78 (1.47) | 23.77 (1.60) | 57.74 (6.10) | <0.01 |
| *Sinapis arvensis* | 6.97 (34.75) | 10.43 (0.55) | 10.64 (33.43) | 14.57 (22.96) | 19.97 (1.70) | <0.01 |
| *Triticum aestivum* | 5.18 (1.27) | 6.29 (0.34) | 63.88 (9.81) | 97.60 (6.23) | 149.12 (11.68) | <0.01 |
| *Vicia faba* | 3.50 (0.31) | 13.18 (0.34) | 61.03 (4.81) | 114.30 (3.74) | 214.07 (10.34) | <0.01 |
| Peak Value | | | | | | |
| *Amaranthus retroflexus* | 4.83 (0.98) | 49.99 (2.00) | 14.51 (1.27) | 22.84 (1.02) | 35.97 (4.16) | <0.01 |
| *Secale cereal* | 5.35 (0.93) | 33.66 (1.22) | 72.65 (7.52) | 109.49 (4.45) | 164.99 (8.83) | <0.01 |
| *Avena Fatua* | 3.23 (0.37) | 47.76 (1.74) | 30.62 (3.79) | 60.43 (3.32) | 119.23 (6.53) | <0.01 |
| *Sinapis arvensis* | 6.88 (25.95) | 31.10 (2.00) | 10.58 (25.38) | 14.56 (17.42) | 20.04 (2.07) | <0.01 |
| *Triticum aestivum* | 2.55 (0.34) | 38.48 (1.84) | 31.38 (5.80) | 74.09 (5.98) | 174.90 (12.84) | <0.01 |
| *Vicia faba* | 2.13 (0.57) | 18.95 (2.04) | 19.11 (8.65) | 53.39 (10.85) | 149.12 (27.05) | <0.01 |
| G Value | | | | | | |
| *Amaranthus retroflexus* | 4.83 (0.98) | 49.99 (2.00) | 14.51 (1.27) | 22.84 (1.02) | 35.97 (4.16) | <0.01 |
| *Secale cereal* | 5.35 (0.93) | 33.66 (1.22) | 72.65 (7.52) | 109.49 (4.45) | 164.99 (8.83) | <0.01 |
| *Avena Fatua* | 3.23 (0.37) | 47.76 (1.74) | 30.62 (3.79) | 60.43 (3.32) | 119.23 (6.53) | <0.01 |
| *Sinapis arvensis* | 6.88 (25.95) | 31.10 (2.00) | 10.58 (25.38) | 14.56 (17.42) | 20.04 (2.07) | <0.01 |
| *Triticum aestivum* | 2.55 (0.34) | 38.48 (1.84) | 31.38 (5.80) | 74.09 (5.98) | 174.90 (12.84) | <0.01 |
| *Vicia faba* | 2.13 (0.57) | 18.95 (2.04) | 19.11 (8.65) | 53.39 (10.85) | 149.12 (27.05) | <0.01 |
| Mean Daily Germination | | | | | | |
| *Amaranthus retroflexus* | 8.81 (6.30) | 7.17 (0.23) | 26.63 (7.81) | 34.17 (3.99) | 43.84 (3.06) | <0.01 |
| *Secale cereal* | 4.29 (0.44) | 6.37 (0.17) | 61.68 (4.82) | 102.83 (3.38) | 171.46 (6.92) | <0.01 |
| *Avena Fatua* | 4.67 (0.64) | 6.13 (0.19) | 47.49 (4.63) | 75.99 (3.08) | 121.59 (5.53) | <0.01 |
| *Sinapis arvensis* | 7.80 (15.58) | 7.14 (0.26) | 12.29 (11.94) | 16.28 (6.68) | 21.57 (3.37) | <0.01 |
| *Triticum aestivum* | 3.10 (0.29) | 6.53 (0.20) | 38.69 (4.08) | 78.44 (3.66) | 159.04 (7.89) | <0.01 |
| *Vicia faba* | 3.14 (0.33) | 6.43 (0.21) | 38.05 (4.30) | 76.54 (3.62) | 153.94 (8.42) | <0.01 |

**Results**
The results section is impossible to follow. Table 1 is overwhelming, with too many parameters that do not show any value or significance of the results. Each parameter is described in a separate subheading. Also, redundant and, for the most part, unreasonable. Not a good enough choice of statistics and presentation of results.

***Response:*** *In response to this comment, and comments above, we have streamlined the results section to keep the most relevant parameters and moved the remaining analyses to the supplementary material. Please also see comments above regarding statistical analysis and changes we have made.*

Table 4: Could you please explain the type of result you present here? You have a molecular formula, but you do not have the percentage content of the compound. In mass spectrometry, retention time, molecular ions [M-H]-, and MS2 fragments (specifically, Q1 and Q3 in a triple quadrupole system) are key parameters used for compound identification and quantification. But they are not a response to the presence of components in the plant extract.

***Response:*** *The data we present are putative compounds as we were unable to validate the targets using known quantities of standards. Although the analysis we did is limited, as we had to work within the limitations of the equipment and standards available to us, it is indicative of the types of compounds that are within the A. repens extract and could be useful to guide future research in this area.*

Line 397-405: In this part of the results, we do not have any valuable information about the content of allelopathic compounds, and which one could be most effective for weed control.

***Response:*** *We have added in additional data on the putative compounds including the m/z and the Match Score (NIST). Due to the limitations explained above, we were unable to generate quantitative data or to validate these peaks with known standards.*

FINALLY, THE COMPLETE RESULTS MUST BE RECALCULATED, AND ALSO THE TEXT OF THIS SECTION AND THE DISCUSSION MUST BE REWRITTEN.

***Response:*** *We have reconfigured the analyses, rewritten the results to ensure the presentation is clear and accurate, and reworked the discussion.*

**Literature**
In this manuscript, you listed 101 literature sources. That amount is usually suitable for a PhD thesis, but not for a research article. Please choose 20-30 of the most relevant references for your research.

***Response****:* *We have streamlined the references ensuring that only the most appropriate are kept.*

**Reviewer 2**

**General comment:** Relevance and Importance: The search for alternatives to chemical herbicides is crucial for sustainable agriculture and environmental protection. The article effectively highlights this necessity.

***General response:*** *We appreciate your valuable comments. We've addressed your questions and rewritten the paper.*

The full scientific name of *Acroptilon repens* (L.) DC. should be provided upon first mention and in the title.

***Response:*** *The scientific names of plant species were written in full.*

The full scientific names of weeds should also be included at their first mention.

***Response:*** *The scientific names of plant species were written in full.*

Lines 82 and 88 contain excessive literature references; three current sources would suffice.

***Response:*** *Some references were removed based on reviewer feedback.*

**Materials and Methods**

The specific parts of the plant material used for the preparation of the aqueous extract should be clarified, as well as the developmental stage of the collected plant, which is currently unspecified.

***Response:*** *The stage of collecting the A. repens plant was before flowering. The parts used include the A. repens vegetative tissues, including leaves and stems. Also, based on the comments of the first reviewer, the experimental design section has been completely rewritten.*

Simply stating stigmasterol and methanesulfonic acid as the "most abundant compounds" may neglect the contributions of other compounds. Allelopathic effects typically arise from the combination of multiple compounds.

***Response:*** *Although we have identified compounds that may be conferring the allelopathic effects of the extract, we have not tested the individually, or in combination, to demonstrate that they are the active compounds. We have altered the text appropriately to make this point more clearly.*

While references to "Table 1" and "Fig 1" are frequent, the tables and figures in the PDF do not consistently match the references in the text or are missing (for instance, "Table 1" and "Table 2" have been merged).

***Response:*** *The results in Table 1 are the results of comparing the average effect of different concentrations on germination and germination characteristics. However, the results in Figure 1 are presented in relative terms for each trait. We have not merged tables one and two. They are plotted separately.*

Combining "Table 1" and "Table 2" into a single table while referencing them separately in the text creates confusion. It is advisable to present the tables in a clearer manner.

***Response:*** *We have not merged tables one and two. They are plotted separately.* *We presented them on separate pages to reduce the possibility of them being merged.*

Although Fig 1c is labeled as "relative to control values" for mean germination time, the MGT values in Table 1 are presented directly relative to concentrations, leading to discrepancies between visual and numerical data.

***Response:*** *Figure C1 is plotted based on* ***RELATIVE*** *mean germination time, while the results of the mean comparison table are the actual mean of the three replicates.*

*For example, we have three numbers 1.55,1.60, and 1.50.*

*The mean of these three numbers is equal to 1.55*

*If we divide each of the above three numbers by the mean, we get three numbers: 1.00, 1.03, and 0.97.*

*The average of the new numbers is equal to 1.00.*

*In this case, you have the relative numbers, which in our figures start from the same point in all plant species.*

*For example, in MGT, this number is equal to 1.00.*

***Consider the two figures below, one based on relative mean germination time and the other based on the mean presented in Table s1.***

Mean Germination Time

| Concentration Russian Knapweed Extract (gL-1) | Amaranthus retroflexus | Secale cereale | Avena fatua | Sinapis arvensis | Triticum aestivum | Vicia faba |
| --- | --- | --- | --- | --- | --- | --- |
| 0 | 1.55 | 2.3 | 1.8 | 2.68333 | 2.18333 | 4.23333 |
| 20 | 2.51667 | 2.31944 | 1.67963 | 4.86667 | 2.21242 | 4.99265 |
| 40 | 4.66667 | 2.30719 | 2.05556 | 0 | 2.24444 | 5.11746 |
| 60 | 2 | 2.44921 | 1.9899 | 0 | 2.29915 | 5.24009 |
| 80 | 0 | 2.16026 | 2.31111 | 0 | 2.18333 | 6.41944 |
| 100 | 0 | 2.16061 | 2.33333 | 0 | 2.0787 | 6.01111 |
| 120 | 0 | 2.20833 | 0 | 0 | 2.05556 | 6.27778 |
| 140 | 0 | 2.46667 | 0 | 0 | 0 | 5.5 |
| 160 | 0 | 0 | 0 | 0 | 0 | 4.66667 |
| 180 | 0 | 0 | 0 | 0 | 0 | 2.33333 |
| 200 | 0 | 0 | 0 | 0 | 0 | 4.66667 |

Relative Mean Germination Time

| Concentration Russian Knapweed Extract (gL-1) | Amaranthus retroflexus | Secale cereale | Avena fatua | Sinapis arvensis | Triticum aestivum | Vicia faba |
| --- | --- | --- | --- | --- | --- | --- |
| 0 | 1 | 1 | 1 | 1 | 1 | 1 |
| 20 | 1.62366 | 1.00845 | 0.933128 | 1.81366 | 1.01332 | 1.17937 |
| 40 | 3.01075 | 1.00313 | 1.14198 | 0 | 1.02799 | 1.20885 |
| 60 | 1.29032 | 1.06487 | 1.1055 | 0 | 1.05304 | 1.23782 |
| 80 | 0 | 0.939242 | 1.28395 | 0 | 1 | 1.5164 |
| 100 | 0 | 0.939394 | 1.2963 | 0 | 0.952078 | 1.41995 |
| 120 | 0 | 0.960145 | 0 | 0 | 0.941476 | 1.48294 |
| 140 | 0 | 1.07246 | 0 | 0 | 0 | 1.29921 |
| 160 | 0 | 0 | 0 | 0 | 0 | 1.10236 |
| 180 | 0 | 0 | 0 | 0 | 0 | 0.551181 |
| 200 | 0 | 0 | 0 | 0 | 0 | 1.10236 |

For Sinapis arvensis*, it was reported that germination was completely inhibited at concentrations above 20 g L-¹, resulting in limited data analysis. This complicates the understanding of the dose-response curve for this species at higher concentrations.

***Response:*** *We initially used R software to conduct dose-response (EC₅₀) analysis; however, the model fit was poor, so we did not proceed with this method for formal interpretation. The R output is provided in the appendix for transparency. Other dose-response tools were considered but found to be less reliable. Instead, we used ANOVA and regression analyses to evaluate germination responses across concentrations. Summary statistics and mean comparisons are presented in Table s1, while detailed outputs are available in the supplementary material.*

| Germination Percentage | | | | | | |
| --- | --- | --- | --- | --- | --- | --- |
| Type of plant | b | d | ED_10_ | ED_50_ | ED_90_ | Lack of fit |
| *Amaranthus retroflexus* | 8.81 (6.29) | 100.43 (3.25) | 26.64 (7.78) | 34.17 (3.97) | 43.84 (3.06) | <0.01 |
| *Secale cereal* | 4.29 (0.44) | 89.27 (2.38) | 61.68 (4.82) | 102.83 (3.38) | 171.46 (6.92) | <0.01 |
| *Avena Fatua* | 4.67 (0.64) | 85.95 (2.76) | 47.49 (4.63) | 75.99 (3.08) | 121.59 (5.53) | <0.01 |
| *Sinapis arvensis* | 7.83 (23.83) | 99.99 (3.71) | 12.30 (18.18) | 16.28 (10.18) | 21.59 (4.99) | <0.01 |
| *Triticum aestivum* | 3.10 (0.29) | 91.47 (2.90) | 38.69 (4.08) | 78.44 (3.66) | 159.04 (7.89) | <0.01 |
| *Vicia faba* | 3.14 (0.33) | 90.11 (2.94) | 38.05 (4.30) | 76.54 (3.62) | 153.94 (8.42) | <0.01 |
| Germination Rate | | | | | | |
| *Amaranthus retroflexus* | 4.20 (0.58) | 14.85 0.38) | 12.54 (0.96) | 21.16 (0.58) | 35.71 (2.81) | <0.01 |
| *Secale cereal* | 4.61 (0.58) | 8.04 (0.24) | 65.50 (5.84) | 105.40 (3.83) | 169.61 (7.60) | <0.01 |
| *Avena Fatua* | 3.52 (0.28) | 13.04 (0.31) | 32.53 (2.51) | 60.67 (2.08) | 113.18 (4.17) | <0.01 |
| *Sinapis arvensis* | 5.44 (13.77) | 8.27 (0.38) | 9.01 (18.20) | 13.50 (13.50) | 20.21 (1.88) | <0.01 |
| *Triticum aestivum* | 2.94 (0.31) | 8.60 (0.30) | 36.74 (4.57) | 77.49 (4.25) | 163.40 (9.12) | <0.01 |
| *Vicia faba* | 2.11 (0.52) | 4.49 (0.42) | 18.93 (7.99) | 53.45 (9.94) | 150.89 (22.41) | <0.01 |
| Germination Index | | | | | | |
| *Amaranthus retroflexus* | 4.20 (0.58) | 14.85 (0.38) | 12.54 (0.96) | 21.16 (0.58) | 35.71 (2.81) | <0.01 |
| *Secale cereal* | 4.61 (0.58) | 8.04 (0.24) | 65.50 (5.84) | 105.40 (3.83) | 169.61 (7.60) | <0.01 |
| *Avena Fatua* | 3.52 (0.28) | 13.04 (0.31) | 32.53 (2.51) | 60.67 (2.08) | 113.18 (4.17) | <0.01 |
| *Sinapis arvensis* | 5.44 (13.77) | 8.27 (0.38) | 9.01 (18.20) | 13.50 (13.50) | 20.21 (1.88) | <0.01 |
| *Triticum aestivum* | 2.94 (0.31) | 8.60 (0.30) | 36.74 (4.57) | 77.49 (4.25) | 163.40 (9.12) | <0.01 |
| *Vicia faba* | 2.11 (0.52) | 4.49 (0.42) | 18.93 (7.99) | 53.45 (9.94) | 150.89 (22.41) | <0.01 |
| Mean Germination Ttime | | | | | | |
| *Amaranthus retroflexus* | 30.74 (9.99) | 2.91 (0.34) | 57.77 (1.67) | 61.27 (1.70) | 60.49 (2.23) | 0.11 |
| *Secale cereal* | 109.14 (9.99) | 2.29 (0.21) | 149.05 (9.66) | 152.08 (9.85) | 155.17 (10.00) | 0.11 |
| *Avena Fatua* | 80.76 (9.99) | 2.02 (0.24) | 109.01 (9.70) | 111.78 (9.94) | 114.61 (10.20) | 0.11 |
| *Sinapis arvensis* | 30.68 (9.99) | 3.77 (0.42) | 30.73 (9.41) | 30.26 (9.98) | 34.63 (10.61) | 0.11 |
| *Triticum aestivum* | 90.75 (9.99) | 2.20 (0.24) | 120.57 (5.04) | 123.31 (5.16) | 126.12 (5.30) | 0.11 |
| *Vicia faba* | 0.12 (4.98) | 5.04 (0.18) | 1.19 *10^49^ (8.98*10^51^) | 9.55*10^56^ (10.00) | 7.62 *10^64^ (5.72*10^67^) | 0.11 |
| Plumule length | | | | | | |
| *Amaranthus retroflexus* | 31.22 (1295.44) | 3.24 (0.25) | 37.15 (113.87) | 39.85 (5.86) | 42.76 (118.57) | <0.01 |
| *Secale cereal* | 2.80 (0.43) | 6.21 (0.29) | 43.44 (7.96) | 95.09 (7.00) | 208.16 (17.00) | <0.01 |
| *Avena Fatua* | 2.87 (0.11) | 21.34 (0.32) | 18.80 (0.87) | 40.40 (0.92) | 86.82 (2.49) | <0.01 |
| *Sinapis arvensis* | 5.63 (5.00) | 7.50 (0.36) | 11.92 (5.51) | 17.61 (2.14) | 26.01 (6. 10) | <0.01 |
| *Triticum aestivum* | 28.05 (24.06) | 6.21 (0.15) | 113.40 (6.13) | 122.64 (2.05) | 132.63 (10.82) | <0.01 |
| *Vicia faba* | 5.24 (1.14) | 3.96 (0.19) | 82.65 (10.57) | 125.71 (6.62) | 191.19 (14.72) | <0.01 |
| Radicle length | | | | | | |
| *Amaranthus retroflexus* | 2.64 (2.05) | 2.08 (0.55) | 9.76 (8.76) | 22.40 (9.59) | 51.44 (32.28) | <0.01 |
| *Secale cereal* | 2.62 (0.43) | 7.88 (0.46) | 34.13 (7.41) | 78.83 (7.38) | 182.07 (17.93) | <0.01 |
| *Avena Fatua* | 2.47 (0.30) | 13.41 (0.54) | 9.78 (1.47) | 23.77 (1.60) | 57.74 (6.10) | <0.01 |
| *Sinapis arvensis* | 6.97 (34.75) | 10.43 (0.55) | 10.64 (33.43) | 14.57 (22.96) | 19.97 (1.70) | <0.01 |
| *Triticum aestivum* | 5.18 (1.27) | 6.29 (0.34) | 63.88 (9.81) | 97.60 (6.23) | 149.12 (11.68) | <0.01 |
| *Vicia faba* | 3.50 (0.31) | 13.18 (0.34) | 61.03 (4.81) | 114.30 (3.74) | 214.07 (10.34) | <0.01 |
| Peak Value | | | | | | |
| *Amaranthus retroflexus* | 4.83 (0.98) | 49.99 (2.00) | 14.51 (1.27) | 22.84 (1.02) | 35.97 (4.16) | <0.01 |
| *Secale cereal* | 5.35 (0.93) | 33.66 (1.22) | 72.65 (7.52) | 109.49 (4.45) | 164.99 (8.83) | <0.01 |
| *Avena Fatua* | 3.23 (0.37) | 47.76 (1.74) | 30.62 (3.79) | 60.43 (3.32) | 119.23 (6.53) | <0.01 |
| *Sinapis arvensis* | 6.88 (25.95) | 31.10 (2.00) | 10.58 (25.38) | 14.56 (17.42) | 20.04 (2.07) | <0.01 |
| *Triticum aestivum* | 2.55 (0.34) | 38.48 (1.84) | 31.38 (5.80) | 74.09 (5.98) | 174.90 (12.84) | <0.01 |
| *Vicia faba* | 2.13 (0.57) | 18.95 (2.04) | 19.11 (8.65) | 53.39 (10.85) | 149.12 (27.05) | <0.01 |
| G Value | | | | | | |
| *Amaranthus retroflexus* | 4.83 (0.98) | 49.99 (2.00) | 14.51 (1.27) | 22.84 (1.02) | 35.97 (4.16) | <0.01 |
| *Secale cereal* | 5.35 (0.93) | 33.66 (1.22) | 72.65 (7.52) | 109.49 (4.45) | 164.99 (8.83) | <0.01 |
| *Avena Fatua* | 3.23 (0.37) | 47.76 (1.74) | 30.62 (3.79) | 60.43 (3.32) | 119.23 (6.53) | <0.01 |
| *Sinapis arvensis* | 6.88 (25.95) | 31.10 (2.00) | 10.58 (25.38) | 14.56 (17.42) | 20.04 (2.07) | <0.01 |
| *Triticum aestivum* | 2.55 (0.34) | 38.48 (1.84) | 31.38 (5.80) | 74.09 (5.98) | 174.90 (12.84) | <0.01 |
| *Vicia faba* | 2.13 (0.57) | 18.95 (2.04) | 19.11 (8.65) | 53.39 (10.85) | 149.12 (27.05) | <0.01 |
| Mean Daily Germination | | | | | | |
| *Amaranthus retroflexus* | 8.81 (6.30) | 7.17 (0.23) | 26.63 (7.81) | 34.17 (3.99) | 43.84 (3.06) | <0.01 |
| *Secale cereal* | 4.29 (0.44) | 6.37 (0.17) | 61.68 (4.82) | 102.83 (3.38) | 171.46 (6.92) | <0.01 |
| *Avena Fatua* | 4.67 (0.64) | 6.13 (0.19) | 47.49 (4.63) | 75.99 (3.08) | 121.59 (5.53) | <0.01 |
| *Sinapis arvensis* | 7.80 (15.58) | 7.14 (0.26) | 12.29 (11.94) | 16.28 (6.68) | 21.57 (3.37) | <0.01 |
| *Triticum aestivum* | 3.10 (0.29) | 6.53 (0.20) | 38.69 (4.08) | 78.44 (3.66) | 159.04 (7.89) | <0.01 |
| *Vicia faba* | 3.14 (0.33) | 6.43 (0.21) | 38.05 (4.30) | 76.54 (3.62) | 153.94 (8.42) | <0.01 |

While natural products are generally safer, high concentrations of identified compounds, such as carvacrol and phenol, may harm beneficial soil organisms and aquatic ecosystems.

***Response:*** *Thank you for pointing this out. If we take this to field trials, we will be sure to include the appropriate eco-tox analyses to ensure that there are no off-target effects. We have highlighted this need in the discussion*

***Carvacrol***

***1. Effects on Soil Microorganisms***

- ***Carvacrol****, a monoterpene found in essential oils, exhibits antimicrobial properties that can affect soil microbial communities and enzyme activities.*
- *Laboratory studies show that carvacrol can inhibit several soil microbial enzyme activities, with observed effect concentrations (EC10 and EC25) for 10% and 25% inhibition ranging from less than 1 µg to several grams per gram of soil. Some soils showed hormesis (stimulation at low doses), but inhibition was clear at higher concentrations.*
- *Soil microbial communities demonstrated resilience, but substrate utilization (especially for polymers and amines) decreased at higher concentrations. The EC50 (concentration causing 50% effect) for soil microorganisms was estimated at about* ***29.79 mg/L*** *over 120 hours.*
- *Soil microorganisms can degrade carvacrol, reducing its long-term ecological risk, but acute effects at high concentrations are possible.*

***2. Effects on Aquatic Ecosystems***

- *Carvacrol displays acute toxicity to aquatic organisms, though soil microorganisms are generally more sensitive. For aquatic bioindicators:*
  - ***Vibrio fischeri****: LC50 (lethal concentration for 50% of organisms) =* ***0.59 mg/L***
  - ***Daphnia magna****: LC50 =* ***4.75 mg/L***
- *No significant inhibition of microbial growth in aquatic communities was observed up to 100 mg/L, but metabolic activity decreased at higher concentrations.*
- *Carvacrol is classified as hazardous to the aquatic environment in some safety data sheets, particularly at higher concentrations.*

***Phenol***

***1. Effects on Soil Microorganisms***

- ***Phenol*** *is toxic to soil bacteria, affecting growth rates and community structure. Direct inhibition of bacterial growth occurs at concentrations above* ***1 µmol/g wet soil*** *(approximately 94 µg/g or 94 mg/kg).*
- *At 1–10 µmol/g soil, bacterial growth can recover within a week, but higher concentrations (30 µmol/g, or about 2.8 mg/g) cause prolonged inhibition.*
- *Phenol contamination can also reduce soil fertility, disrupt enzyme activities, and alter microbial community composition, with some recovery possible through microbial adaptation or degradation.*

***2. Effects on Aquatic Ecosystems***

- *Phenol is bioaccumulative and highly toxic to aquatic organisms, especially fish, causing physiological and histopathological changes at low concentrations.*
- *Chronic safe concentration for aquatic ecosystems is estimated at* ***0.63 mg/L****; concentrations above this threshold can cause toxic effects in sensitive species such as fish and algae.*
- *Regulatory standards for phenol in drinking water are typically set at* ***0.002 mg/L*** *in some countries, reflecting its high toxicity and potential for bioaccumulation.*

**Some references:**

*Wang, Z., Zhang, H., Zhang, D., Wang, Y., Han, Y., Xue, X., & Jiang, Y. (2024). Biodegradation of phenol-contaminated soil and plant growth promotion by Myroides xuanwuensis H13. Microbiology Spectrum, 12(8), e00266-24.*

*Zeng, X., Yu, J., Zhang, S., Ni, T., & Ma, D. (2023). Ecological risk of phenol on typical biota of the northern Chinese river from an integrated probability perspective: the Hun River as an example. Environmental Monitoring and Assessment, 195(12), 1512.*

*Thiele-Bruhn, S., Shikuku, V., Dittrich, F., Torjir, D. N., Saini, M., & Getenga, Z. (2024). Soil sorption and effects on soil microorganisms of thymol and carvacrol monoterpenes from essential oils of aromatic plants. Frontiers in Environmental Science, 12, 1379018.*

*Gan, C., Langa, E., Ballestero, D., & Pino-Otín, M. R. (2024). Comparative ecotoxicity assessment of highly bioactive isomeric monoterpenes carvacrol and thymol on aquatic and edaphic indicators and communities. Chemosphere, 368, 143666.*

*Demoling, L. A., & Bååth, E. (2008). Use of pollution‐induced community tolerance of the bacterial community to detect phenol toxicity in soil. Environmental Toxicology and Chemistry, 27(2), 334-340.*

*Abdel-Hameid, N. A. H. (2007). Physiological and histopathological alterations induced by phenol exposure in Oreochromis aureus juveniles. Turkish Journal of Fisheries and Aquatic Sciences, 7(2).*

It is evident that the quantity of plant materials used to obtain extracts can negatively impact their application potential. Suggested methods should be developed to mitigate this issue.

***Response:*** *We thank the reviewer for raising this point. We agree that the quantity of plant material required for extract preparation is an important consideration in evaluating the potential for field or farm scale application. In this manuscript, we have not considered scalability or evaluated the feasibility of application with regards to large-scale application – however, our data simply focused on whether A. repens extract had bioherbicidal activity against problematic rather than desirable species; as it does, our research then opens the door for future research into whether or not it could be scaled up appropriately.*

Further research is essential to transform this potential into a commercial product. Specifically, the extract needs to be standardized, active ingredients isolated, and their efficacy tested both individually and in combination under field conditions.

***Response:*** *We agree with your idea. This article is a preliminary study. However, more field studies are needed to report the herbicidal effects of A. repens. The potential of the identified allelopathic compounds should be investigated separately. We have generally mentioned the need for further research in the conclusion section.* ***“More research is needed to identify which, if any, of the putatively identified active allelopathic compounds are responsible for the reductions we observe and to then derive a mechanistic understanding of how they influence germination and seedling vigor.”***
