# Supplementary material for: Under controlled conditions, lower concentrations of aqueous extracts of Acroptilon repens are needed to inhibit seed germination and seedling growth of weeds than crops
Source: PLoS One. 2026 Aug 27;21(8):e0354454. doi: 10.1371/journal.pone.0354454 (PMC13521396; doi:10.1371/journal.pone.0354454)
Supplement: Supplementary file 4 [file pone.0354454.s011.docx]

**Manuscript Title:** Bioherbicidal potential of *Acroptilon repens* (Russian knapweed) aqueous extracts

**Manuscript ID:** PONE-D-25-29786R1

**Journal:** PLOS ONE

**Corresponding Authors:** Dr. Mohammad Taghi Alebrahim & Dr Dana MacGregor

Date of Resubmission:

**Dear Dr. Uludag and Reviewers**,

Thank you very much for your thoughtful and constructive feedback on our manuscript titled *“Allelopathic potential of Acroptilon repens (Russian knapweed) aqueous extract for managing agriculturally important weed species in comparison to relevant crops”* (Manuscript ID: PONE-D-25-29786R1). We appreciate the time and effort you and the reviewers have invested in evaluating our work.

We are pleased to learn that the manuscript was found to have merit, and that the majority of the reviewers’ comments were addressed in the previous draft. We have carefully considered all remaining suggestions provided by both the reviewers and the academic editor, and we have revised the manuscript accordingly to improve its clarity, scientific rigor, and overall presentation.

In the following sections, we provide a detailed point-by-point response to each comment, indicating how we have addressed the concerns raised. It is important to note that the References section now includes two additional references, numbered 55 and 56, corresponding to the newly identified compounds in Extraction Method 2. The entire reference list has been renumbered to follow the revised sequence. We hope that the revised manuscript now meets the standards required for publication in PLOS ONE.

_____

We thank Dr. Uludag for the editorial feedback and helpful suggestions to improve the clarity and presentation of our manuscript. We have addressed each point as follows:

**Comment 1. Suggested Title and Running Title**

- We have adopted the suggested title: *“Bioherbicidal potential of Acroptilon repens (Russian knapweed) aqueous extracts”*.
- The running title has been updated to: *“Bioherbicidal potential of Acroptilon repens”*.

**2. Use of Passive and Active Voice**

- We have revised the manuscript to ensure we used the passive voice where appropriate. Sentences previously written in active voice have been adjusted to passive where appropriate, particularly in the Methods and Results sections. For example:
  - Original: “We assessed its impact on seed germination and early growth…” 🡪 changed to “… was assessed.”
  - Original: “This study evaluates how the aqueous extract of *A. repens* influences germination and growth metrics of *A. retroflexus*, *A. fatua*, *S. arvensis*, and *S. cereale* compared to *T. aestivum* and *V. faba.*

**3. Problematic Sentence Regarding Wheat and Corn Fields**

- The sentence describing the impact of Russian knapweed on wheat and corn fields has been rephrased for clarity. The revised version avoids ambiguity and improves readability:
  - Revised: “The presence of Russian knapweed in wheat and corn fields can significantly reduce yield and affect flour quality due to bitter-tasting seed contamination.”

**4. Materials and Methods Opening Sentence**

- As suggested, we have removed the introductory sentence from the Materials and Methods section to streamline the narrative and avoid redundancy.

**5. Use of the Term “Noxious Weed”**

- We have replaced the term “noxious weed” with more appropriate terminology, such as “invasive” or “problematic weed species,” to reflect ecological and agronomic relevance without implying regulatory classification.

We hope these revisions meet your expectations and contribute to a clearer and more scientifically robust manuscript.

______

**Reviewer 3**

We thank Reviewer #3 for their positive assessment of our manuscript and for acknowledging that all previous comments have been addressed. We appreciate the additional feedback provided and have made further revisions to improve the clarity and scientific value of the manuscript.

**Comment 1:** *“Results are clear and understandable, except for untargeted GC-MS.”*

**Response:** We have revised the section describing the untargeted GC-MS analysis to improve clarity and ensure that the methodology and interpretation are more accessible to readers. Specifically, we have:

- Clarified the process of extractions in the materials and methods section.
- Made it more clear that compound identifications were provisional, but that the identifications were justified based on the data we have.
- Rearranged the compound within the table so that it is clear where the confidence scores are confident and those that are putative to enhance transparency.

**Comment 2:** *“It is however, a common study, done numerously by other researchers on other weed species.”*

**Response:** We acknowledge that allelopathic studies using aqueous extracts have been conducted on various weed species – we refer to other studies in the paper throughout making sure that our work is put into the context of what has been done previously. We also are careful to explain why our work is unique. We believe our study is among the first to systematically compare the bioherbicidal effects of *Acroptilon repens* on both weed and crop species using a comprehensive set of germination and growth parameters. We have tried to make this clearer throughout.

**Comment 3:** *“Some sentences are confusing and need to be rephrased.”*

**Response:** We have carefully reviewed the manuscript and revised several sentences for clarity and readability. Examples include:

- Rewriting the sentence about Russian knapweed’s impact on wheat and corn fields to improve flow and precision.
- Simplifying complex sentence structures in the Introduction and Results sections.

**Comment 4:** *“The paper would produce a better impact if study on the isolation of a target compound were to be done and presented in the manuscript.”*

**Response:** We agree that isolating and characterizing specific bioactive compounds would enhance the impact of the study. However, this was beyond the scope of the current work, which aimed to evaluate the overall bioherbicidal potential of the aqueous extract. We have added a statement in the Discussion section acknowledging this limitation and proposing targeted compound isolation as a priority for future research.

_____

**Reviewer 4**

We thank Reviewer #4 for their careful review and constructive comments. We appreciate the acknowledgment that most of the previous concerns have been addressed and have made further revisions to improve the manuscript based on the remaining suggestions.

**Comment 1:** *“In Table 3, the NIST match for some compounds is very low. It is important to select matches higher than 80%, or to use standard compounds or retention indices for identification. Please consider this issue.”*

**Response:** We appreciate this important observation regarding compound identification. In response, we have:

- Re-evaluated the GC-MS data and removed compounds with NIST match scores below 80% unless they were supported by additional evidence (e.g., retention time consistency or literature reports).
- Added a note in the Methods section clarifying the threshold used for compound identification.
- Included a revised version of Table 3 with updated compound matches and confidence scores.
- Acknowledged the limitation of relying solely on NIST library matches and emphasized the need for future work involving standard compounds and retention indices for more definitive identification.

We hope these revisions address the reviewer’s concerns and improve the reliability of our chemical profiling.
