# Supplementary material for: Under controlled conditions, lower concentrations of aqueous extracts of Acroptilon repens are needed to inhibit seed germination and seedling growth of weeds than crops
Source: PLoS One. 2026 Aug 27;21(8):e0354454. doi: 10.1371/journal.pone.0354454 (PMC13521396; doi:10.1371/journal.pone.0354454)
Supplement: Supplementary file 5 [file pone.0354454.s012.docx]

**Response to Reviewers**

**General comment:** *We thank the reviewers for their constructive feedback. Below are our detailed responses to each comment.*

# Reviewer #5:

Subject:

Comments and Observations on Manuscript [Bioherbicidal potential of *Acroptilon repens* L. (Russian knapweed) aqueous extracts]

Dear Editor’s,

After a thorough review of the submitted manuscript titled (Bioherbicidal potential of *Acroptilon repens* L. (Russian knapweed) aqueous extracts), we would like to bring to your attention several observations and points that may require clarification or revision. These observations are summarized below under scientific, methodological, linguistic, and reference-related aspects:

## 1. Scientific / Methodological Issues

### • Experimental Design:

o The rationale for selecting the concentrations (0–200 g L⁻¹) was not explained, nor was it indicated whether these levels are field-applicable.

- **Response:** *Thank you for raising this point. The concentrations (0 to 200 g L⁻¹) were selected to cover a broad range for initial screening purposes. This study aimed to first and foremost establish a baseline understanding of whether the extract would be effective; therefore, considerations regarding field applicability were outside the scope of this experiment.*

o Inconsistencies in reporting the number of seeds per replicate (60 seeds mentioned in some sections, not in others) need correction.

- **Response:** *We appreciate your observation. We have changed the text to make it clearer that three technical replicates of 20 seeds per replicate were used and that the total seeds per analysis was 60. The number of seeds per replicate has been standardized and made consistent throughout the manuscript.*

o Environmental control was limited to temperature; humidity and ventilation in the incubator were not mentioned.

- **Response:** *Thank you for noting this. The plates containing the seeds were sealed and therefore humidity and ventilation details of the chamber itself did not affect the conditions that the seeds were experiencing. We have added text that clarifies this and highlighted that the aim was to ensure consistent and equivalent conditions between the dishes.*

### • Statistical Analysis:

o Only LSD at 5% was reported, without verification of ANOVA assumptions such as variance homogeneity.

o Pearson correlation was applied to all traits, which may be inappropriate if some data are non-normal or highly heterogeneous.

- **Response:** *We acknowledge your point. ANOVA assumptions were checked, and this is now stated in the revised manuscript. Data normality was assessed using the Shapiro–Wilk test, and non-normal data were normalized using a square root transformation. Homogeneity of variances was verified using Levene’s test. After confirming these assumptions, ANOVA was performed, and mean comparisons were conducted using LSD at 5% significance. Pearson correlation: Pearson correlation was applied only to traits that met the assumptions of normality and homogeneity of variance. For traits that did not meet these assumptions, appropriate square root transformations were applied prior to correlation analysis to ensure valid results.*

### • Measurement of Indicators:

o Some indices (GP, GR, GI, PV, GV) are complex and repetitive; simplification or merging is recommended.

- ***Response:*** *Thank you for your advice. The data regarding the indices you highlight are in the Supplementary Data section. Although perhaps complex and repetitive, they are quantitative, robust measurements for different seed germination or seedling growth parameters and may prove to be useful for other researchers who would like to do future studies. The main data in this manuscript includes only the key parameters of GP, GR, MGT, and the length of the shoot and root of the seedling. We argue that simplification or merging would result in the loss of useful data.*

o Discrepancies exist between text and tables (e.g., MGT), sometimes stating no significant differences, sometimes reporting large increases.

- **Response:** *We have simplified the presentation of indices and ensured consistency between text and tables. We also moved the mean comparison table from the Supplementary Data section to the main part of the manuscript to resolve any ambiguities created based on Figure 1 (relative data).*

### • Result Interpretation:

o Statements generalizing sensitivity of invasive species are based on limited data.

- **Response:** *Many reports on the bioactivity of extracts report few or limited data. We have tested three weedy species (Sinapis arvensis L., Amaranthus retroflexus L., and Avena fatua L.), a multipurpose species (Secale cereale L.), and two crop species (Triticum aestivum L. and Vicia faba L.), in appropriate technical replicates. Although the dataset is relatively limited, we consider it useful and comparable to, or more comprehensive than, datasets reported in previous studies.*

o Lack of negative controls or sufficient replicates to assess natural variation.

- **Response:** *Negative controls, where no extract was added, are included for all germination parameters. We agree that we have not assessed the potential range of responses that may be occurring across the natural variation of the target species; however, the main aim of this paper is to establish that application of A. repens extract can have an effect across a range of representative species. Therefore, analysis of natural variation either within different sources of A. repens as the extract source, or of the target species themselves, was outside the scope of this work. We aimed to establish a baseline for whether the extract had activity and on which species, rather than how effective it is under field conditions.*

o Claims about “biological effects” from GC-MS are speculative, based solely on compound presence without experimental validation.

- **Response:** *We agree that GC-MS findings are indicative and not confirmatory. We have repeatedly used the words "putatively" and "Putative" in various sections of the GC-MS results and discussion to indicate that the GC-MS results are speculative. However, we explicitly mentioned in the final section of the discussion that future studies require compound isolation and experimental validation.*

## 2. Linguistic / Stylistic Issues

• Repetition of species names within the same sentence.

- **Response:** *Because we are using multiple species, we aimed for clarity. The repetition of species names in one sentence is more likely due to differences in A. repens concentrations in inhibiting germination or their different sensitivities.*

*Such as “Germination was fully inhibited for S. arvensis ≥ 40 g L⁻¹, A. retroflexus ≥ 80 g L⁻¹, A. fatua ≥ 120 g L⁻¹, T. aestivum ≥ 140 g L⁻¹, S. cereale ≥ 160 g L⁻¹, and V. faba ≥ 200 g L⁻¹” or “Radicle lengths varied greatly between the species. The longest radicle length belonged to A. fatua > S. arvensis > V. faba, respectively. Secale cereale, and T. aestivum had similar radicle length.”*

*In other cases, the phrase "all tested species" was used.*

• Very long and complex sentences that reduce readability.

- ***Response:*** *The manuscript has been carefully revised to improve readability and clarity. Additionally, it was further refined for language and grammatical accuracy using the Springer Nature language editing service.*

• Use of technical terms without definition (e.g., PV).

- **Response:** *PV (Peak Value) was defined in the Materials and Methods section on line 336 (Revised Manuscript with track changes). All abbreviations related to tables are also provided in the form of captions.*

• Spelling errors (e.g., “inhabitation” → inhibition, “Secale cereal” → Secale cereale, “thefinal” → the final).

- **Response:** *All noted linguistic and spelling errors have been corrected.*

## 3. References

• Some references are repeated or inconsistent; order should be corrected.

- **Response:** *We have edited the reference list to ensure there are no duplicate references in the reference list. In some cases, several publications from the same author in different years have been used, but they are not duplicates. Also, due to the limited references on the allelopathic compounds of A. repens, we have had to use some articles that are clearly not similar to our work with the aim of making sure no unrelated references are used. The referencing has been standardized as appropriate for the journal.*

• Some references are outdated (pre-2000), with no justification for relevance.

- **Response:** *As we answered in the previous question, due to the limited resources on the allelopathic compounds of A. repens and the lack of a similar article to our manuscript, we were forced to use some relatively similar sources that have previously addressed the identification of allelopathic compounds. However, we would argue that simply being an older reference does not make it irrelevant.*

• Figure and citation inconsistencies (e.g., Fig 1d vs. Fig 1 e).

- **Response:** *We appreciate the careful observation of the reviewer and have worked to ensure any differences in figure citations have been corrected.*

## 4. Additional Observations

• No comparison with field studies; all results are laboratory-based.

- **Response:** *We clarify that the present study was intentionally designed as a controlled laboratory experiment with the primary objective of establishing a fundamental biological baseline for the allelopathic activity of Acroptilon repens extract. At this exploratory stage, the focus is on determining whether the extract exhibits measurable biological effects under standardized conditions, rather than on assessing agronomic performance. Comparisons with field-based studies were therefore not included, as such comparisons would not be methodologically appropriate at this phase of investigation. Field experiments require prior evidence of biological efficacy, as well as optimization of formulation, dose, and application methods, which have not yet been established for A. repens extract. Furthermore, as noted in the manuscript, extrapolation between different extract types or between laboratory and field conditions should be approached with caution, given substantial differences in chemical composition, bioavailability, and interactions with environmental factors. Consequently, the laboratory-based approach adopted here represents a necessary and scientifically justified first step toward future applied or field-oriented research.*

• Use of the term “bioherbicide” without toxicity or environmental assessment.

- **Response:** *Thank you for this point. We agree with you that the term "bioherbicides" cannot be used without a toxicity assessment. We have revised the two sentences that we had clearly mentioned.*

*“Although comprehensive ecological and agronomic assessments are needed prior to field application and further research is needed to isolate and confirm the bioactivity of these potentially phytotoxic secondary metabolites, these data support the potential use of A. repens aqueous extract as a natural, broad-spectrum allopathic agent, particularly as it is more active against the weeds than the crops we tested herein.”*

*“However, these data demonstrate that A. repens extract has the potential to be used as an allelopathic germination inhibitor.”*

• Study limitations, such as differences between fresh and stored seeds, were not stated.

- ***Response:*** *We have added further clarity about the collection or acquisition and storage of seeds into the Materials and Methods section. We have also added text to the Discussion explicitly acknowledging 1) that the two classes of plants (weeds and crops) were from seeds that were acquired or purchased and therefore had different storage histories, and 2) although they showed high germination without* A. repens *extract, that these differences may have affected their responses and therefore contributed to the distinct sensitivity patterns observed between weedy and cultivated species. We have also noted that future work should use seeds with equivalent age, storage conditions, and vigour to better isolate biological responses from seed‑handling effects.*

• Some tables (Tables 1–4) were not included, complicating result verification.

- **Response:** *We ensured that all tables were included in the text and in the upload. In this manuscript, we presented five tables, including the results of the analysis of variance, comparison of means, linear slope, and Pearson’s R2 values, and tables related to the GC-MS results. The other tables are related to supplementary data, which have been made available to PLOS One Journal in separate files.*

• Occasional grammatical inconsistencies, especially with commas and parentheses.

- ***Response:*** *Thank you for reading the manuscript very carefully. We have double-checked this text with our native English-speaking author and used the Springer Nature editor to correct the article's readability and grammatical errors. If further errors are found, we are happy to correct them in the typesetting and final editing stages.*

## Recommendations for Improvement:

• Correct all scientific and spelling errors, including species names.

• Simplify complex sentences and indicators.

• Clarify rationale for extraction concentrations and field applicability.

• Interpret results cautiously without overgeneralization.

• Review references for consistency and relevance.

• Explicitly mention study limitations and potential practical constraints.

• Indicate that GC-MS findings are speculative unless experimentally validated.

- **Response:** *We appreciate that these comments have helped improve the clarity, accuracy, and scientific rigor of the manuscript. We feel we have addressed each of these points above with further detail.*

# Reviewer #6:

The manuscript Bioherbicidal potential of Acroptilon repens L. (Russian knapweed) aqueous extracts is very interesting. However, it is not clear to me why GC–MS was used for the characterization of the extract instead of HPLC. Please clarify. Additionally, is it possible to perform an HPLC analysis to better characterize the chemical composition of the extract?

- **Response:** *Thank you for this suggestion. Untargeted GC-MS was chosen because it is well-suited for volatile and semi-volatile compounds present in the extract. While HPLC could provide complementary information, it was beyond the scope of this initial screening study. We will keep that in mind if we can obtain further funding to conduct follow-up studies on this promising extract.*

Furthermore, in Tables 3 and 4 only the detected substances are listed, without their relative abundance. The representation (quantitative share) of these components is very important.

- **Response:** *We appreciate this observation. Unfortunately, we were unable to generate absolute measures for the putatively identified compounds; our untargeted analysis was performed without compound‑specific calibration. We used an Agilent 7890B GC coupled to a 5977A MSD. The software computes Area (%) values which represent the relative abundance of detected compounds based on their integrated peak areas in the GC–MS chromatogram response rather than absolute concentrations. We have added this text into the Materials and methods, Results and Discussions explaining this and highlighting this as a limitation of our study.*
