# Supplementary material for: Under controlled conditions, lower concentrations of aqueous extracts of Acroptilon repens are needed to inhibit seed germination and seedling growth of weeds than crops
Source: PLoS One. 2026 Aug 27;21(8):e0354454. doi: 10.1371/journal.pone.0354454 (PMC13521396; doi:10.1371/journal.pone.0354454)
Supplement: Supplementary file 6 [file pone.0354454.s013.docx]

PONE-D-25-29786R3

Bioherbicidal potential of Acroptilon repens L. (Russian knapweed) aqueous extracts

***Reviewer #7***s' comment: Thank you for submitting the revised version of your paper. You have answered all the questions of the reviewers in appropriate way. Your responses are very satisfactory and I am fully agreed with your responses which you have also incorporated in the revised version of your manuscript in appropriate places.

***Authors:*** We thank Review #7 for their careful reading of our manuscript and are happy to hear that they were satisfied that all comments have been addressed and the manuscript is ready to be accepted.

Additional Editor Comments:

**Editor:** I got different views from the reviewers. One of the reviewers accepted wihle the other rejected. I think he pointed out some good points to improve this paper. I have fully agreed some of his statements, see below:

the manuscript repeatedly refers to "bioherbicidal potential", "allelopathic effects", and "broad-spectrum allelopathic agent". However, the experimental design is limited to in vitro germination and seedling growth assays, which demonstrate phytotoxic effects under controlled conditions, not allelopathy in an ecological sense. No evidence is provided regarding release into soil, persistence, or plant–plant interactions. Therefore, the use of the term "bioherbicide" in the title and the interpretation in terms of allelopathy are not adequately supported and should be reconsidered".

***AUTHORS***: We thank the Academic Editor and reviewers for their careful and constructive evaluation of our manuscript. In particular, we have addressed the conceptual concerns raised regarding the interpretation of our results by substantially revising the title, abstract, discussion, and conclusions to clearly delimit the scope of the study. Although *Acroptilon repens* is well established in the literature as an allelopathic species, we now explicitly distinguish between previously reported allelopathic behaviour and the *in vitro* phytotoxic effects assessed here. All terminology implying ecological allelopathy or bioherbicidal activity unsupported by the experimental design has been removed or qualified, and methodological limitations are now clearly acknowledged. We believe these revisions ensure that the manuscript accurately reflects the data presented, is appropriately contextualized within the existing literature, and aligns fully with the reviewer and editorial guidance.

**Editor:** In addition, could you please see some other literature as he mentioned.

***AUTHORS***: We have added additional literature references, particularly in the introduction to highlight the considerable amount of research that has established *A. repens* as an allelopathic plant with demonstrable ability to repress seedling germination and establishment. We have also added in text that highlights how our study differs from this body of literature and what aspects of it are unique and novel.

**Editor:** Explain your procedure clearly and discuss methodological difference with earlier literature.

***AUTHORS***: We have tightened the methodology sections of the paper and clarified how our protocols match and differ from earlier literature.

**Editor:** Discuss and connect clearly your bio data with GCMS data.

***AUTHORS***: We have added in additional text to discuss and connect our biological data with the GC-MS data. As there is considerable data about the allelopathic metabolites within and released by *A. repens*, we have reframed this section to be exploratory data that are confirming the previous findings rather than conclusive evidence of what compounds are causing the observed effects. We have also streamlined the discussion text so that it is focused on what is probably active and what is probably just structural components.

Reviewer #8's Responses to Questions 3. Has the statistical analysis been performed appropriately and rigorously?

Reviewer #8: Yes

Reviewer #8's Response to Question 5. Is the manuscript presented in an intelligible fashion and written in standard English?

Reviewer #8: Yes

AUTHORS: We appreciate that Reviewer #8 recognizes that our paper is statistically sound and written in intelligible fashion in standard English.

________________________________________

2. Is the manuscript technically sound, and do the data support the conclusions? Reviewer #8: No

AUTHORS: As evidenced in the tracked changes, we have altered the manuscript to make it more technically sound, ensuring that all the conclusions are supported by the data.

4. Have the authors made all data underlying the findings in their manuscript fully available? Reviewer #8: No

AUTHORS: We have ensured that all of the data are available by effectively using the tables and figures within the paper, adding in supplementary files where appropriate.

________________________________________

6. Review Comments to the Author

***Reviewer #8:*** The manuscript entitled "Bioherbicidal potential of Acroptilon repens L. aqueous extracts" investigates the effects of aqueous extracts on seed germination and early seedling growth of several plant species.

While the study presents a dataset that is, in principle, technically usable, the work is affected by substantial conceptual, methodological, and interpretative limitations. In particular, it suffers from overstatement of the biological relevance of the results, insufficient integration with the existing literature, inconsistencies in the extraction procedures, and limited and weakly supported chemical characterization.

***AUTHORS***: We thank the reviewer for this detailed assessment. We agree that earlier versions of the manuscript overstated the biological relevance of the findings, did not sufficiently contextualize the work within the existing literature, and did not adequately acknowledge methodological and interpretative limitations. In response, we have substantially revised the manuscript to address each of these concerns, as detailed below.

***Reviewer #8:*** First, the manuscript repeatedly refers to "bioherbicidal potential", "allelopathic effects", and "broad-spectrum allelopathic agent". However, the experimental design is limited to in vitro germination and seedling growth assays, which demonstrate phytotoxic effects under controlled conditions, not allelopathy in an ecological sense. No evidence is provided regarding release into soil, persistence, or plant–plant interactions. Therefore, the use of the term "bioherbicide" in the title and the interpretation in terms of allelopathy are not adequately supported and should be reconsidered.

***AUTHORS***: We agree with the reviewer that the experimental design employed in this study, e.g. *in vitro* germination and seedling growth assays, does not directly test allelopathic interactions in an ecological or soil‑mediated context. We have highlighted this limitation in the new version of the text. At the same time, we note that *Acroptilon repens* has been widely described as an allelopathic species in the literature, based on a long history of observations from field and soil‑based assays [Fletcher & Renney, 1963] and considerable in vitro assays as detailed in the text. To avoid conflating these established properties with the specific scope of our experiments, we have revised the manuscript to clearly distinguish between previously reported allelopathic behaviour of *A. repens* and the *in vitro* phytotoxic effects demonstrated here. Accordingly, the term “bioherbicide” has been removed from the title, which has been altered to better reflect the findings of the paper, and references to allelopathy have been restricted to background context, while interpretation of our results is now limited to inhibitory or phytotoxic effects observed under controlled conditions.

***Reviewer #8:*** Second, the manuscript does not adequately engage with the existing literature on A. repens. Several relevant studies on aqueous extracts, powdered plant material, essential oils, and ecological interactions are not sufficiently discussed. As a result, the work is not properly contextualized and appears largely confirmatory rather than novel.

***AUTHORS***: We thank the reviewer for this comment. We acknowledge that the version read contains revisions, made in response to prior reviewer guidance, that substantially reduced discussion of the allelopathic literature on *Acroptilon repens*. This was done with the aim of avoiding conflating previous work with the scope of our *in vitro* assays. In the current revision, we have expanded text that discusses relevant *A. repens* studies. For instance, we have expanded the Introduction to better contextualize the study within the existing literature on *A. repens*, explicitly summarising prior work on aqueous extracts and germination responses. This revised framing situates the present results within the existing literature while maintaining a clear distinction between previously reported allelopathic behaviour and the specific phytotoxic effects demonstrated here.

***Reviewer #8:*** Third, the extraction procedure used for the bioassays raises concerns regarding reproducibility. Different concentrations (0–200 g/L) are prepared independently rather than obtained from a single standardized extract. This approach does not ensure consistent extraction efficiency or comparable chemical composition across treatments and introduces methodological variability that is not discussed.

***AUTHORS***: We thank the reviewer for raising this point. We would like to clarify that all extract concentrations were prepared from a **single, pooled, and homogenized bulk sample of dried *A. repens* material**, rather than from independently sourced plant material. Different concentrations were generated by varying the mass of this same bulk powder relative to a fixed volume of distilled water, thereby maintaining a consistent plant source across treatments. We recognize that this was not sufficiently explicit in the previous version of the Methods section and have revised the text to clearly state the use of a single homogenized batch. We have also added clarification regarding the implications of this extraction approach for reproducibility and interpretation in the Discussion.

***Reviewer #8:*** A major limitation concerns the lack of correspondence between the extracts used in biological assays and those analysed by GC–MS. The GC–MS analysis is performed on extracts prepared using a different procedure (1:10 w/v extraction with additional processing steps), which does not reflect the extracts used in the bioassays. Consequently, the reported chemical composition cannot be directly linked to the observed biological effects, and the interpretation relating specific compounds to activity is not sufficiently supported.

In addition, the chemical characterization is limited using GC–MS alone, which does not capture the full range of compounds present in aqueous extracts, particularly polar constituents. Compound identification is putative and based on library matching, while the reported relative abundances (area %) do not provide quantitative information. Therefore, linking specific compounds to the observed biological activity is not sufficiently supported.

***AUTHORS***: We thank the reviewer for their view on this matter. We agree that GC–MS alone does not capture the full chemical complexity of aqueous extracts, particularly polar constituents, and that compound identification based on library matching is necessarily putative. In response, we have dramatically revised the manuscript to explicitly present the GC–MS results as qualitative and hypothesis‑generating rather than quantitative or causal. We now clarify that relative peak areas do not provide absolute concentrations and that no direct linkage between specific compounds and observed biological effects can be inferred from the present data.

At the same time, we note that *Acroptilon repens* has been extensively studied in the literature using complementary approaches, and the compounds detected here are consistent with classes of phytotoxic and allelopathic metabolites previously reported for this species. We have revised the Discussion to use the existing literature for contextualisation rather than attribution of causality, and we explicitly acknowledge that targeted chemical analyses and bioactivity‑guided fractionation would be required to establish mechanistic links.

In response to this comment, we have revised the manuscript to remove data from the “method 1“ extraction, and clarify how the aqueous extract was generated in the same way as those used for biological activity with the exception of the steps needed to convert the aqueous extract to something suitable for GC injection and that they were 10:1 w/v (not 1:10 w/v as noted above), clearly state the limitations of GC–MS for aqueous extracts, present compound identification as “putative“ and “non‑quantitative“, and restrict discussion of detected metabolites to contextualization within the existing literature rather than attribution of causal biological activity.

We have also ensured that the peaks that were identified have high NSIT matches and were not probable contaminations from the extraction method itself. We have altered the discussion appropriately.

***Reviewer #8:*** The description of sample processing (drying followed by lyophilization) is also unclear and requires clarification, as the rationale for combining these steps is not adequately explained.

***AUTHORS***: We thank the reviewer for raising this concern and recognize that the description of the extraction procedure in the previous version did not sufficiently emphasize the steps taken to ensure reproducibility. As we state above, **all bioassay and GC–MS extractions were prepared from a single pooled, homogenized bulk sample of dried *Acroptilon repens* plant material**, which was finely ground prior to extraction. We have revised the Methods section to explicitly distinguish between air‑drying of plant material prior to generating the aqueous extract and post‑extraction drying of aqueous extracts for GC-MS analysis. Air‑drying was used to stabilize plant tissue and ensure consistent mass‑to‑volume ratios before extraction, whereas subsequent drying and lyophilization of the liquid extracts were performed to concentrate aqueous extracts and obtain solvent‑free residues suitable for GC–MS analysis. The rationale for this two‑step approach is now clearly explained in the revised manuscript.

***Reviewer #8:*** The M&M section would benefit from improved clarity and detail. In particular, the origin of seeds is insufficiently documented (supplier and batch information are not provided), and storage conditions are described in vague terms. Some methodological descriptions are redundant or not optimally structured.

***AUTHORS***: We thank the reviewer for this constructive comment. The Materials and Methods section has been revised to improve clarity, reproducibility, and structure. Specifically, we have expanded the description of seed origin to include supplier details and batch information for commercially sourced material, and the location, collection timing, and post-harvest handling of field-collected seeds. Storage conditions are now described explicitly, including temperature, light exposure, and storage duration. In addition, redundant methodological descriptions have been removed and related procedures consolidated into clearly structured sections to improve readability. We have also clarified the distinction between extracts used for biological assays and those prepared for GC–MS analysis to ensure accurate interpretation of the chemical data. These revisions ensure that the methodology is fully transparent and reproducible.

***Reviewer #8:*** The Results section is largely descriptive and, in several cases, lacks sufficient quantitative interpretation. The organization by individual indices leads to fragmentation, and some analyses (e.g., correlations) appear of limited relevance.

***AUTHORS***: We thank the reviewer for this helpful comment. The Results section has been considerably revised to improve clarity, quantitative interpretation, and overall structure. Specifically, we have reorganized the presentation to focus on biologically relevant patterns (e.g., overall concentration response, weed vs crop comparison, and species-specific sensitivity) rather than reporting each germination index separately, thereby reducing fragmentation. Redundant descriptions of individual indices have been consolidated, and additional quantitative summary statements have been incorporated to highlight the magnitude and comparative nature of observed effects. Correlation analyses have been substantially condensed, moved to the supplementary figures and explicitly linked to the main biological interpretation where retained. These changes improve readability and ensure that the Results more clearly convey the key findings of the study.

***Reviewer #8:*** The Discussion largely reiterates the results and lacks critical comparison with previous studies. It also includes speculative links between detected compounds and biological activity that are not adequately supported by the data.

***AUTHORS***: We thank the reviewer for this constructive comment. In response, we have substantially revised the Discussion to reduce repetition of the Results, strengthen comparison with prior studies, and clearly delimit interpretation of the chemical data. The revised Discussion now emphasizes the novelty of the side‑by‑side comparison of weed and crop species relative to existing literature, while avoiding re‑statement of quantitative results. All discussion of GC–MS data has been explicitly reframed as qualitative and contextual, with causal or mechanistic claims removed or qualified. We now clearly state that compound identification is putative, relative abundances are non‑quantitative, and that bioactivity attribution requires future targeted and bioactivity‑guided studies. We believe these revisions address the reviewer’s concerns and ensure that the Discussion accurately reflects both the scope and limitations of the data.

***Reviewer #8:*** Finally, the Conclusions section reflects the same tendency toward overinterpretation and would benefit from a more cautious alignment with the actual scope of the experimental evidence.

***AUTHORS***: We thank the reviewer for this comment and agree that the previous Conclusions section was redundant and unnecessarily reiterated the scope of the experimental evidence. In response, we have ensured that content from the former Conclusions has been integrated into the Discussion, where it is now explicitly contextualized alongside methodological limitations and relevant literature, and the standalone Conclusions section has been removed. This restructuring avoids repetition and ensures that final interpretative statements remain appropriately cautious and evidence‑based.
