# Supplementary material for: Under controlled conditions, lower concentrations of aqueous extracts of Acroptilon repens are needed to inhibit seed germination and seedling growth of weeds than crops
Source: PLoS One. 2026 Aug 27;21(8):e0354454. doi: 10.1371/journal.pone.0354454 (PMC13521396; doi:10.1371/journal.pone.0354454)
Supplement: Peer review history

# Peer review: Decision Letter 0

**DOI:** 10.1371/journal.pone.0354454.r001

**Authors:** Ahmet Uludag

7 Jul 2025

Dear Dr. Alebrahim,

Thank you for submitting your manuscript to PLOS ONE. After careful consideration, we feel that it has merit but does not fully meet PLOS ONE’s publication criteria as it currently stands. Therefore, we invite you to submit a revised version of the manuscript that addresses the points raised during the review process.

I would like to thank to reviewer 1 for her very detailed review. Your manuscript needs new organization and language improvement. A good study needs better presenting. I have found your work invaluable, which it can lead further studies.

Please submit your revised manuscript by Aug 21 2025 11:59PM. If you will need more time than this to complete your revisions, please reply to this message or contact the journal office at plosone@plos.org. When you're ready to submit your revision, log on to https://www.editorialmanager.com/pone/ and select the 'Submissions Needing Revision' folder to locate your manuscript file.

- A rebuttal letter that responds to each point raised by the academic editor and reviewer(s). You should upload this letter as a separate file labeled 'Response to Reviewers'.
- A marked-up copy of your manuscript that highlights changes made to the original version. You should upload this as a separate file labeled 'Revised Manuscript with Track Changes'.
- An unmarked version of your revised paper without tracked changes. You should upload this as a separate file labeled 'Manuscript'.

If you would like to make changes to your financial disclosure, please include your updated statement in your cover letter. Guidelines for resubmitting your figure files are available below the reviewer comments at the end of this letter.

If applicable, we recommend that you deposit your laboratory protocols in protocols.io to enhance the reproducibility of your results. Protocols.io assigns your protocol its own identifier (DOI) so that it can be cited independently in the future. For instructions see: https://journals.plos.org/plosone/s/submission-guidelines#loc-laboratory-protocols. Additionally, PLOS ONE offers an option for publishing peer-reviewed Lab Protocol articles, which describe protocols hosted on protocols.io. Read more information on sharing protocols at https://plos.org/protocols?utm_medium=editorial-email&utm_source=authorletters&utm_campaign=protocols.

We look forward to receiving your revised manuscript.

Kind regards,

Ahmet Uludag, Ph.D.

Academic Editor

PLOS ONE

Journal Requirements:

When submitting your revision, we need you to address these additional requirements.

1. Please ensure that your manuscript meets PLOS ONE's style requirements, including those for file naming. The PLOS ONE style templates can be found at https://journals.plos.org/plosone/s/file?id=wjVg/PLOSOne_formatting_sample_main_body.pdf and https://journals.plos.org/plosone/s/file?id=ba62/PLOSOne_formatting_sample_title_authors_affiliations.pdf

2. When completing the data availability statement of the submission form, you indicated that you will make your data available on acceptance. We strongly recommend all authors decide on a data sharing plan before acceptance, as the process can be lengthy and hold up publication timelines. Please note that, though access restrictions are acceptable now, your entire data will need to be made freely accessible if your manuscript is accepted for publication. This policy applies to all data except where public deposition would breach compliance with the protocol approved by your research ethics board. If you are unable to adhere to our open data policy, please kindly revise your statement to explain your reasoning and we will seek the editor's input on an exemption. Please be assured that, once you have provided your new statement, the assessment of your exemption will not hold up the peer review process.

Additional Editor Comments :

This is a good study comparing to similar studies. However, it needs to be well organized and rewritten. I think you presented more statistical analysis in your abstract. I recommend using passive sentences are better in our area. Tltle can be "Conteol potential of aqueous extract of Acroptilon repens (Russian knapweed) on some important weed species". Could you please carefully follow the reviewers' recommendations.

[Note: HTML markup is below. Please do not edit.]

Reviewers' comments:

Reviewer's Responses to Questions

**Comments to the Author**

1. Is the manuscript technically sound, and do the data support the conclusions?

Reviewer #1: Partly

Reviewer #2: Yes

**********

2. Has the statistical analysis been performed appropriately and rigorously?

Reviewer #1: No

Reviewer #2: Yes

**********

3. Have the authors made all data underlying the findings in their manuscript fully available?

The PLOS Data policy

Reviewer #1: No

Reviewer #2: Yes

**********

4. Is the manuscript presented in an intelligible fashion and written in standard English?

Reviewer #1: No

Reviewer #2: Yes

**********

Reviewer #1: Dear Authors,

I have many concerns about this research. In my opinion, the manuscript was written below the standards of the journal PLOS ONE. The research is at a preliminary stage, with inadequately chosen parameters and highly questionable results. Most parts of the abstract, introduction, material and methods, results, and conclusion must be rewritten. In this form, the text is very difficult to read. ENGLISH LANGUAGE EDITING AND STYLE POLISHING FOR THIS MANUSCRIPT ARE NECESSARY. In this form, my recommendation for this manuscript is REJECT. The authors must be making a MAJOR REVISION and resubmitting the manuscript (it is necessary to expand the effect of aqueous extract in the research on plants, to calculate and present the results in another way, etc).

Comments:

Line 1: Title: The title is not adequate; it is more of a declarative sentence. Please see below my proposal for the manuscript title:

Allelopathic potential of aqueous extract of Acroptilon repens (Russian knapweed) for management of noxious weeds

Introduction

Lines 46-56: Traditional management approaches frequently depend on chemical inputs, particularly herbicides, to mitigate the adverse impacts of weeds on agricultural output [1]. The overuse and indiscriminate application of these substances, however, results in herbicide resistance [2], significant environmental issues, such as the degradation of agricultural ecosystems, accumulation of chemical residues, and the contamination of vital resources like air, water, and soil [3,4]. Therefore, in line with environmental preservation and economic sustainability measures, less harmful but equally effective alternatives are needed for successful weed management strategies in agriculture production [5].

Please replace with text below:

Traditional management approaches often rely on chemical inputs, particularly herbicides, to mitigate the adverse effects of weeds on agricultural yields [1]. The overuse and indiscriminate application of these substances result in herbicide resistance [2], as well as significant environmental issues, including the degradation of agricultural ecosystems, the accumulation of chemical residues, and the contamination of vital resources such as air, water, and soil [3,4]. Therefore, in line with environmental preservation and economic sustainability measures, less harmful but equally effective alternatives are needed for successful weed management strategies in agricultural production [5].

Lines 57-78: This part of the introduction has basic information and a definition of allelopathy. It is well-known literature and need not be detailed in the introduction. Please delete this part, or write one or two sentences about this.

Lines 79-117: This part is written too extensively. A lot of unnecessary information is written. In contrast, the objectives of the work are unclear.

Lines 117-120: This part is written like a conclusion, but an introduction should contain clear objectives of the research. In addition, it is impossible to draw such conclusions based on the results presented in the manuscript. Only a seed bioassay was performed here, which is the basis for preliminary research. After that, it is necessary to do a plant bioassay in controlled conditions and the field. This type of research can not give us clear answers about the effectiveness of plant extracts in controlling weed species and their herbicidal potential.

FINALLY, THE COMPLETE INTRODUCTION MUST BE REWRITTEN.

Material and methods

The methods of work and the material used are written unreasonably and include too much unnecessary information (for example: “were obtained from the Seed and Plant Improvement Institute of Iran, introduced in 1991.” “were also obtained from the Seed and Plant Improvement Institute of Iran, where it was introduced in 2017.” “Both seeds were provided from the Moghan Research and Natural Resources Center a month before the experiment and stored in a cool, dark environment.”).

Also, I do not agree with all the selected measured parameters. In general, in addition to the percentage of germination, germination rate, and seedling length, other parameters are not of great importance for this type of research.

Decide whether to use Latin or common names. It is unnecessary to repeat both several times.

Line 144: “affected by infection”, - What did you mean? Weeds may be present in a wheat field, but they are not an infection.

You have a wide range of solution concentrations from 2% to 20%. It would be better to calculate the dose-response EC50 using R or another program. Correlation is not the best method for this type of data.

Results

The results section is impossible to follow. Table 1 is overwhelming, with too many parameters that do not show any value or significance of the results. Each parameter is described in a separate subheading. Also, redundant and, for the most part, unreasonable. Not a good enough choice of statistics and presentation of results.

Table 4: Could you please explain the type of result you present here? You have a molecular formula, but you do not have the percentage content of the compound. In mass spectrometry, retention time, molecular ions [M-H]-, and MS2 fragments (specifically, Q1 and Q3 in a triple quadrupole system) are key parameters used for compound identification and quantification. But they are not a response to the presence of components in the plant extract.

Line 397-405: In this part of the results, we do not have any valuable information about the content of allelopathic compounds, and which one could be most effective for weed control.

The manuscript has about 40 pages. It is too much, too much unnecessary and repeated information.

FINALLY, THE COMPLETE RESULTS MUST BE RECALCULATED, AND ALSO THE TEXT OF THIS SECTION AND THE DISCUSSION MUST BE REWRITTEN.

Literature

In this manuscript, you listed 101 literature sources. That amount is usually suitable for a PhD thesis, but not for a research article. Please choose 20-30 of the most relevant references for your research.

Reviewer #2: Relevance and Importance: The search for alternatives to chemical herbicides is crucial for sustainable agriculture and environmental protection. The article effectively highlights this necessity.

The full scientific name of Acroptilon repens (L.) DC. should be provided upon first mention and in the title.

The full scientific names of weeds should also be included at their first mention.

Lines 82 and 88 contain excessive literature references; three current sources would suffice.

Materials and Methods

The specific parts of the plant material used for the preparation of the aqueous extract should be clarified, as well as the developmental stage of the collected plant, which is currently unspecified.

Simply stating stigmasterol and methanesulfonic acid as the "most abundant compounds" may neglect the contributions of other compounds. Allelopathic effects typically arise from the combination of multiple compounds.

While references to "Table 1" and "Fig 1" are frequent, the tables and figures in the PDF do not consistently match the references in the text or are missing (for instance, "Table 1" and "Table 2" have been merged).

Combining "Table 1" and "Table 2" into a single table while referencing them separately in the text creates confusion. It is advisable to present the tables in a clearer manner.

Although Fig 1c is labeled as "relative to control values" for mean germination time, the MGT values in Table 1 are presented directly relative to concentrations, leading to discrepancies between visual and numerical data.

For Sinapis arvensis*, it was reported that germination was completely inhibited at concentrations above 20 g L-¹, resulting in limited data analysis. This complicates the understanding of the dose-response curve for this species at higher concentrations.

While natural products are generally safer, high concentrations of identified compounds, such as carvacrol and phenol, may harm beneficial soil organisms and aquatic ecosystems.

It is evident that the quantity of plant materials used to obtain extracts can negatively impact their application potential. Suggested methods should be developed to mitigate this issue.

Further research is essential to transform this potential into a commercial product. Specifically, the extract needs to be standardized, active ingredients isolated, and their efficacy tested both individually and in combination under field conditions.

**********

what does this mean?). If published, this will include your full peer review and any attached files.

If you choose “no”, your identity will remain anonymous but your review may still be made public.

**Do you want your identity to be public for this peer review?**  For information about this choice, including consent withdrawal, please see our Privacy Policy

Reviewer #1: No

Reviewer #2: **Yes:** Koray Kaçan

**********

[NOTE: If reviewer comments were submitted as an attachment file, they will be attached to this email and accessible via the submission site. Please log into your account, locate the manuscript record, and check for the action link "View Attachments". If this link does not appear, there are no attachment files.]

While revising your submission, please upload your figure files to the Preflight Analysis and Conversion Engine (PACE) digital diagnostic tool, https://pacev2.apexcovantage.com/. PACE helps ensure that figures meet PLOS requirements. To use PACE, you must first register as a user. Registration is free. Then, login and navigate to the UPLOAD tab, where you will find detailed instructions on how to use the tool. If you encounter any issues or have any questions when using PACE, please email PLOS at figures@plos.org. Please note that Supporting Information files do not need this step.

Attachment Submitted filename: Comments to authors.docx

# Author comment: Author response to Decision Letter 1

**DOI:** 10.1371/journal.pone.0354454.r002

27 Aug 2025

Additional Editor Comments:

This is a good study comparing to similar studies. However, it needs to be well organized and rewritten. I think you presented more statistical analysis in your abstract. I recommend using passive sentences are better in our area. Title can be "Control potential of aqueous extract of Acroptilon repens (Russian knapweed) on some important weed species". Could you please carefully follow the reviewers' recommendations.

Response: We appreciate both the Editor’s and Reviewer 1’s comments on our title. We have generated a title that combines the best of both suggestions and emphasizes that we also studied the relevant crop species - “Allelopathic potential of Acroptilon repens (Russian knapweed) aqueous extract for managing agriculturally important weed species in comparison to relevant crops”.

Reviewer 1

Dear Authors,

General comment: I have many concerns about this research. In my opinion, the manuscript was written below the standards of the journal PLOS ONE. The research is at a preliminary stage, with inadequately chosen parameters and highly questionable results. Most parts of the abstract, introduction, material and methods, results, and conclusion must be rewritten. In this form, the text is very difficult to read. ENGLISH LANGUAGE EDITING AND STYLE POLISHING FOR THIS MANUSCRIPT ARE NECESSARY. In this form, my recommendation for this manuscript is REJECT. The authors must be making a MAJOR REVISION and resubmitting the manuscript (it is necessary to expand the effect of aqueous extract in the research on plants, to calculate and present the results in another way, etc).

General response: We appreciate your valuable comments. We've addressed your questions and rewritten the paper.

Comments:

Line 1: Title: The title is not adequate; it is more of a declarative sentence. Please see below my proposal for the manuscript title:

Allelopathic potential of aqueous extract of Acroptilon repens (Russian knapweed) for management of noxious weeds

Response: We have changed the title combining this suggestion with the one made by the Editor.

Introduction

Lines 46-56: Traditional management approaches frequently depend on chemical inputs, particularly herbicides, to mitigate the adverse impacts of weeds on agricultural output [1]. The overuse and indiscriminate application of these substances, however, results in herbicide resistance [2], significant environmental issues, such as the degradation of agricultural ecosystems, accumulation of chemical residues, and the contamination of vital resources like air, water, and soil [3,4]. Therefore, in line with environmental preservation and economic sustainability measures, less harmful but equally effective alternatives are needed for successful weed management strategies in agriculture production [5].

Response: The text written by the referee was replaced.

Lines 57-78: This part of the introduction has basic information and a definition of allelopathy. It is well-known literature and need not be detailed in the introduction. Please delete this part, or write one or two sentences about this.

Response: We removed the definitions related to allelopathy and rewrote the introduction.

Lines 79-117: This part is written too extensively. A lot of unnecessary information is written. In contrast, the objectives of the work are unclear.

Response: The introduction has been completely rewritten.

Lines 117-120: This part is written like a conclusion, but an introduction should contain clear objectives of the research. In addition, it is impossible to draw such conclusions based on the results presented in the manuscript. Only a seed bioassay was performed here, which is the basis for preliminary research. After that, it is necessary to do a plant bioassay in controlled conditions and the field. This type of research can not give us clear answers about the effectiveness of plant extracts in controlling weed species and their herbicidal potential.

Response: The introduction has been completely rewritten.

FINALLY, THE COMPLETE INTRODUCTION MUST BE REWRITTEN.

Response: The introduction has been completely rewritten.

Material and methods

The methods of work and the material used are written unreasonably and include too much unnecessary information (for example: “were obtained from the Seed and Plant Improvement Institute of Iran, introduced in 1991.” “were also obtained from the Seed and Plant Improvement Institute of Iran, where it was introduced in 2017.” “Both seeds were provided from the Moghan Research and Natural Resources Center a month before the experiment and stored in a cool, dark environment.”).

Response: We removed unnecessary sections based on the referee's opinion and rewrote the materials and methods.

Also, I do not agree with all the selected measured parameters. In general, in addition to the percentage of germination, germination rate, and seedling length, other parameters are not of great importance for this type of research.

Response: Based on the Reviewer’s suggestion, we have moved the data pertaining to the other parameters to supplementary files – Figure 1 and the tables in the main paper now only deal with percentage of germination, germination rate, and seedling length. We kept the data concerning Mean Germination Time in the main paper as it was the one parameter unchanged by application of A. repens extract.

Decide whether to use Latin or common names. It is unnecessary to repeat both several times.

Response: Common names were removed.

Line 144: “affected by infection”, - What did you mean? Weeds may be present in a wheat field, but they are not an infection.

Response: By the word infection, we meant "the excessive and unwanted presence of A. repens weeds in a wheat field." The sentence was rewritten.

You have a wide range of solution concentrations from 2% to 20%. It would be better to calculate the dose-response EC50 using R or another program. Correlation is not the best method for this type of data.

Response: We initially used R software to conduct dose-response (EC₅₀) analysis; however, the model fit was poor, so we did not proceed with this method for formal interpretation. The R output is provided in the appendix for transparency. Other dose-response tools were considered but found to be less reliable. Instead, we used ANOVA and regression analyses to evaluate germination responses across concentrations. Summary statistics and mean comparisons are presented in Table 1, while detailed outputs are available in the supplementary material.

Germination Percentage

Type of plant b d ED10 ED50 ED90 Lack of fit

Amaranthus retroflexus 8.81 (6.29) 100.43 (3.25) 26.64 (7.78) 34.17 (3.97) 43.84 (3.06) <0.01

Secale cereal 4.29 (0.44) 89.27 (2.38) 61.68 (4.82) 102.83 (3.38) 171.46 (6.92) <0.01

Avena Fatua 4.67 (0.64) 85.95 (2.76) 47.49 (4.63) 75.99 (3.08) 121.59 (5.53) <0.01

Sinapis arvensis 7.83 (23.83) 99.99 (3.71) 12.30 (18.18) 16.28 (10.18) 21.59 (4.99) <0.01

Triticum aestivum 3.10 (0.29) 91.47 (2.90) 38.69 (4.08) 78.44 (3.66) 159.04 (7.89) <0.01

Vicia faba 3.14 (0.33) 90.11 (2.94) 38.05 (4.30) 76.54 (3.62) 153.94 (8.42) <0.01

Germination Rate

Amaranthus retroflexus 4.20 (0.58) 14.85 0.38) 12.54 (0.96) 21.16 (0.58) 35.71 (2.81) <0.01

Secale cereal 4.61 (0.58) 8.04 (0.24) 65.50 (5.84) 105.40 (3.83) 169.61 (7.60) <0.01

Avena Fatua 3.52 (0.28) 13.04 (0.31) 32.53 (2.51) 60.67 (2.08) 113.18 (4.17) <0.01

Sinapis arvensis 5.44 (13.77) 8.27 (0.38) 9.01 (18.20) 13.50 (13.50) 20.21 (1.88) <0.01

Triticum aestivum 2.94 (0.31) 8.60 (0.30) 36.74 (4.57) 77.49 (4.25) 163.40 (9.12) <0.01

Vicia faba 2.11 (0.52) 4.49 (0.42) 18.93 (7.99) 53.45 (9.94) 150.89 (22.41) <0.01

Germination Index

Amaranthus retroflexus 4.20 (0.58) 14.85 (0.38) 12.54 (0.96) 21.16 (0.58) 35.71 (2.81) <0.01

Secale cereal 4.61 (0.58) 8.04 (0.24) 65.50 (5.84) 105.40 (3.83) 169.61 (7.60) <0.01

Avena Fatua 3.52 (0.28) 13.04 (0.31) 32.53 (2.51) 60.67 (2.08) 113.18 (4.17) <0.01

Sinapis arvensis 5.44 (13.77) 8.27 (0.38) 9.01 (18.20) 13.50 (13.50) 20.21 (1.88) <0.01

Triticum aestivum 2.94 (0.31) 8.60 (0.30) 36.74 (4.57) 77.49 (4.25) 163.40 (9.12) <0.01

Vicia faba 2.11 (0.52) 4.49 (0.42) 18.93 (7.99) 53.45 (9.94) 150.89 (22.41) <0.01

Mean Germination Ttime

Amaranthus retroflexus 30.74 (9.99) 2.91 (0.34) 57.77 (1.67) 61.27 (1.70) 60.49 (2.23) 0.11

Secale cereal 109.14 (9.99) 2.29 (0.21) 149.05 (9.66) 152.08 (9.85) 155.17 (10.00) 0.11

Avena Fatua 80.76 (9.99) 2.02 (0.24) 109.01 (9.70) 111.78 (9.94) 114.61 (10.20) 0.11

Sinapis arvensis 30.68 (9.99) 3.77 (0.42) 30.73 (9.41) 30.26 (9.98) 34.63 (10.61) 0.11

Triticum aestivum 90.75 (9.99) 2.20 (0.24) 120.57 (5.04) 123.31 (5.16) 126.12 (5.30) 0.11

Vicia faba 0.12 (4.98) 5.04 (0.18) 1.19 *1049 (8.98*1051) 9.55*1056 (10.00) 7.62 *1064 (5.72*1067) 0.11

Plumule length

Amaranthus retroflexus 31.22 (1295.44) 3.24 (0.25) 37.15 (113.87) 39.85 (5.86) 42.76 (118.57) <0.01

Secale cereal 2.80 (0.43) 6.21 (0.29) 43.44 (7.96) 95.09 (7.00) 208.16 (17.00) <0.01

Avena Fatua 2.87 (0.11) 21.34 (0.32) 18.80 (0.87) 40.40 (0.92) 86.82 (2.49) <0.01

Sinapis arvensis 5.63 (5.00) 7.50 (0.36) 11.92 (5.51) 17.61 (2.14) 26.01 (6. 10) <0.01

Triticum aestivum 28.05 (24.06) 6.21 (0.15) 113.40 (6.13) 122.64 (2.05) 132.63 (10.82) <0.01

Vicia faba 5.24 (1.14) 3.96 (0.19) 82.65 (10.57) 125.71 (6.62) 191.19 (14.72) <0.01

Radicle length

Amaranthus retroflexus 2.64 (2.05) 2.08 (0.55) 9.76 (8.76) 22.40 (9.59) 51.44 (32.28) <0.01

Secale cereal 2.62 (0.43) 7.88 (0.46) 34.13 (7.41) 78.83 (7.38) 182.07 (17.93) <0.01

Avena Fatua 2.47 (0.30) 13.41 (0.54) 9.78 (1.47) 23.77 (1.60) 57.74 (6.10) <0.01

Sinapis arvensis 6.97 (34.75) 10.43 (0.55) 10.64 (33.43) 14.57 (22.96) 19.97 (1.70) <0.01

Triticum aestivum 5.18 (1.27) 6.29 (0.34) 63.88 (9.81) 97.60 (6.23) 149.12 (11.68) <0.01

Vicia faba 3.50 (0.31) 13.18 (0.34) 61.03 (4.81) 114.30 (3.74) 214.07 (10.34) <0.01

Peak Value

Amaranthus retroflexus 4.83 (0.98) 49.99 (2.00) 14.51 (1.27) 22.84 (1.02) 35.97 (4.16) <0.01

Secale cereal 5.35 (0.93) 33.66 (1.22) 72.65 (7.52) 109.49 (4.45) 164.99 (8.83) <0.01

Avena Fatua 3.23 (0.37) 47.76 (1.74) 30.62 (3.79) 60.43 (3.32) 119.23 (6.53) <0.01

Sinapis arvensis 6.88 (25.95) 31.10 (2.00) 10.58 (25.38) 14.56 (17.42) 20.04 (2.07) <0.01

Triticum aestivum 2.55 (0.34) 38.48 (1.84) 31.38 (5.80) 74.09 (5.98) 174.90 (12.84) <0.01

Vicia faba 2.13 (0.57) 18.95 (2.04) 19.11 (8.65) 53.39 (10.85) 149.12 (27.05) <0.01

G Value

Amaranthus retroflexus 4.83 (0.98) 49.99 (2.00) 14.51 (1.27) 22.84 (1.02) 35.97 (4.16) <0.01

Secale cereal 5.35 (0.93) 33.66 (1.22) 72.65 (7.52) 109.49 (4.45) 164.99 (8.83) <0.01

Avena Fatua 3.23 (0.37) 47.76 (1.74) 30.62 (3.79) 60.43 (3.32) 119.23 (6.53) <0.01

Sinapis arvensis 6.88 (25.95) 31.10 (2.00) 10.58 (25.38) 14.56 (17.42) 20.04 (2.07) <0.01

Triticum aestivum 2.55 (0.34) 38.48 (1.84) 31.38 (5.80) 74.09 (5.98) 174.90 (12.84) <0.01

Vicia faba 2.13 (0.57) 18.95 (2.04) 19.11 (8.65) 53.39 (10.85) 149.12 (27.05) <0.01

Mean Daily Germination

Amaranthus retroflexus 8.81 (6.30) 7.17 (0.23) 26.63 (7.81) 34.17 (3.99) 43.84 (3.06) <0.01

Secale cereal 4.29 (0.44) 6.37 (0.17) 61.68 (4.82) 102.83 (3.38) 171.46 (6.92) <0.01

Avena Fatua 4.67 (0.64) 6.13 (0.19) 47.49 (4.63) 75.99 (3.08) 121.59 (5.53) <0.01

Sinapis arvensis 7.80 (15.58) 7.14 (0.26) 12.29 (11.94) 16.28 (6.68) 21.57 (3.37) <0.01

Triticum aestivum 3.10 (0.29) 6.53 (0.20) 38.69 (4.08) 78.44 (3.66) 159.04 (7.89) <0.01

Vicia faba 3.14 (0.33) 6.43 (0.21) 38.05 (4.30) 76.54 (3.62) 153.94 (8.42) <0.01

Results

The results section is impossible to follow. Table 1 is overwhelming, with too many parameters that do not show any value or significance of the results. Each parameter is described in a separate subheading. Also, redundant and, for the most part, unreasonable. Not a good enough choice of statistics and presentation of results.

Response: In response to this comment, and comments above, we have streamlined the results section to keep the most relevant parameters and moved the remaining analyses to the supplementary material. Please also see comments above regarding statistical analysis and changes we have made.

Table 4: Could you please explain the type of result you present here? You have a molecular formula, but you do not have the percentage content of the compound. In mass spectrometry, retention time, molecular ions [M-H]-, and MS2 fragments (specifically, Q1 and Q3 in a triple quadrupole system) are key parameters used for compound identification and quantification. But they are not a response to the presence of components in the plant extract.

Response: The data we present are putative compounds as we were unable to validate the targets using known quantities of standards. Although the analysis we did is limited, as we had to work within the limitations of the equipment and standards available to us, it is indicative of the types of compounds that are within the A. repens extract and could be useful to guide future research in this area.

Line 397-405: In this part of the results, we do not have any valuable information about the content of allelopathic compounds, and which one could be most effective for weed control.

Response: We have added in additional data on the putative compounds including the m/z and the Match Score (NIST). Due to the limitations explained above, we were unable to generate quantitative data or to validate these peaks with known standards.

FINALLY, THE COMPLETE RESULTS MUST BE RECALCULATED, AND ALSO THE TEXT OF THIS SECTION AND THE DISCUSSION MUST BE REWRITTEN.

Response: We have reconfigured the analyses, rewritten the results to ensure the presentation is clear and accurate, and reworked the discussion.

Literature

In this manuscript, you listed 101 literature sources. That amount is usually suitable for a PhD thesis, but not for a research article. Please choose 20-30 of the most relevant references for your research.

Response: We have streamlined the references ensuring that only the most appropriate are kept.

Reviewer 2

General comment: Relevance and Importance: The search for alternatives to chemical herbicides is crucial for sustainable agriculture and environmental protection. The article effectively highlights this necessity.

General response: We appreciate your valuable comments. We've addressed your questions and rewritten the paper.

The full scientific name of Acroptilon repens (L.) DC. should be provided upon first mention and in the title.

Response: The scientific names of plant species were written in full.

The full scientific names of weeds should also be included at their first mention.

Response: The scientific names of plant species were written in full.

Lines 82 and 88 contain excessive literature references; three current sources would suffice.

Response: Some references were removed based on reviewer feedback.

Materials and Methods

The specific parts of the plant material used for the preparation of the aqueous extract should be clarified, as well as the developmental stage of the collected plant, which is currently unspecified.

Response: The stage of collecting the A. repens plant was before flowering. The parts used include the A. repens vegetative tissues, including leaves and stems. Also, based on the comments of the first reviewer, the experimental design section has been completely rewritten.

Simply stating stigmasterol and methanesulfonic acid as the "most abundant compounds" may neglect the contributions of other compounds. Allelopathic effects typically arise from the combination of multiple compounds.

Response: Although we have identified compounds that may be conferring the allelopathic effects of the extract, we have not tested the individually, or in combination, to demonstrate that they are the active compounds. We have altered the text appropriately to make this point more clearly.

While references to "Table 1" and "Fig 1" are frequent, the tables and figures in the PDF do not consistently match the references in the text or are missing (for instance, "Table 1" and "Table 2" have been merged).

Response: The results in Table 1 are the results of comparing the average effect of different concentrations on germination and germination characteristics. However, the results in Figure 1 are presented in relativ

Attachment Submitted filename: Response to Refeers.docx

# Peer review: Decision Letter 1

**DOI:** 10.1371/journal.pone.0354454.r003

**Authors:** Ahmet Uludag

17 Oct 2025

Thank you for submitting your manuscript to PLOS ONE. After careful consideration, we feel that it has merit but does not fully meet PLOS ONE’s publication criteria as it currently stands. Therefore, we invite you to submit a revised version of the manuscript that addresses the points raised during the review process.

I have added my comments below. Could you please make corrections accordingly both reviewer's and my suggestions.

Please submit your revised manuscript by Dec 01 2025 11:59PM. If you will need more time than this to complete your revisions, please reply to this message or contact the journal office at plosone@plos.org. When you're ready to submit your revision, log on to https://www.editorialmanager.com/pone/ and select the 'Submissions Needing Revision' folder to locate your manuscript file.

- A rebuttal letter that responds to each point raised by the academic editor and reviewer(s). You should upload this letter as a separate file labeled 'Response to Reviewers'.
- A marked-up copy of your manuscript that highlights changes made to the original version. You should upload this as a separate file labeled 'Revised Manuscript with Track Changes'.
- An unmarked version of your revised paper without tracked changes. You should upload this as a separate file labeled 'Manuscript'.

If applicable, we recommend that you deposit your laboratory protocols in protocols.io to enhance the reproducibility of your results. Protocols.io assigns your protocol its own identifier (DOI) so that it can be cited independently in the future. For instructions see: https://journals.plos.org/plosone/s/submission-guidelines#loc-laboratory-protocols. Additionally, PLOS ONE offers an option for publishing peer-reviewed Lab Protocol articles, which describe protocols hosted on protocols.io. Read more information on sharing protocols at https://plos.org/protocols?utm_medium=editorial-email&utm_source=authorletters&utm_campaign=protocols.

We look forward to receiving your revised manuscript.

Kind regards,

Ahmet Uludag, Ph.D.

Academic Editor

PLOS ONE

Journal Requirements:

1. If the reviewer comments include a recommendation to cite specific previously published works, please review and evaluate these publications to determine whether they are relevant and should be cited. There is no requirement to cite these works unless the editor has indicated otherwise.

2. Please review your reference list to ensure that it is complete and correct. If you have cited papers that have been retracted, please include the rationale for doing so in the manuscript text, or remove these references and replace them with relevant current references. Any changes to the reference list should be mentioned in the rebuttal letter that accompanies your revised manuscript. If you need to cite a retracted article, indicate the article’s retracted status in the References list and also include a citation and full reference for the retraction notice.

**Additional Editor Comments:**

I have some more suggestions in addition to reviewers' suggestions.

Suggestion for title: Bioherbicidal potential of Acroptilon repens (Russian knapweed) Aqueous extracts

Suggested running title: Bioherbicidal potential of Acroptilon repens

The manuscript is mainly written using passive voice. Some active sentences also can be written in passive voice. Examples are: “We assessed its impact on seed germination and early growth of three weedy species (Sinapis arvensis, Amaranthus retroflexus, and Avena fatua) and three crop species (Secale cereale, Triticum aestivum, and Vicia faba)”. “Herein we evaluate how the aqueous extract of Russian knapweed (Acroptilon repens L.) influences germination and growth indicators of redroot pigweed (Amaranthus retroflexus), wild oats (Avena fatua), wild mustard (Sinapis arvensis), wheat (Triticum aestivum L.), fava bean (Vicia faba) and cereal rye (Secale cereale L”.

There are some problematic sentences which makes reading difficult. AN example is following: When Russian knapweed is present in wheat and corn fields, it dramatically reduces both yield and flour quality. Even small amounts of cross-contamination from Russian knapweed seeds into the wheat cause the flour to taste bitter [34,35].

Materials and methods start with a sentences which is not necessary and this sentence should be deleted: This research is designed to assess the impact of Russian knapweed (Acroptilon repens L.) extract on germination of various problematic weed species, including redroot pigweed (Amaranthus retroflexus) and wild mustard (Sinapis arvensis), as well as common crops such as wheat (Triticum aestivum L.) and fava bean (Vicia faba). Additionally, it will examine crops with weedy characteristics, including wild oat (Avena fatua) and wild rye (Secale cereale L.).

Noxios weed is a term which is mainly used for regulated weed species by legislation.

[Note: HTML markup is below. Please do not edit.]

Reviewers' comments:

Reviewer's Responses to Questions

**Comments to the Author**

Reviewer #3: All comments have been addressed

Reviewer #4: All comments have been addressed

**********

2. Is the manuscript technically sound, and do the data support the conclusions?

Reviewer #3: Yes

Reviewer #4: Partly

**********

3. Has the statistical analysis been performed appropriately and rigorously?

Reviewer #3: Yes

Reviewer #4: Yes

**********

4. Have the authors made all data underlying the findings in their manuscript fully available?

The PLOS Data policy

Reviewer #3: Yes

Reviewer #4: Yes

**********

5. Is the manuscript presented in an intelligible fashion and written in standard English?

Reviewer #3: Yes

Reviewer #4: Yes

**********

Reviewer #3: The manuscript investigated the potential of Russian knapweed to produce herbicidal activity against several weed species and crops. Results are clear and understandable, except for untargeted GS-MS. It is however, a common study, done numerously by other researchers on other weed species. Some sentences are confusing and need to be rephrased. The paper would produce a better impact if study on the isolation of a target compound were to be done and presented in the manuscript

Reviewer #4: The authors have responded to the comments; however, there are still some areas that need improvement.

In Table 3, the NIST match for some compounds is very low. It is important to select matches higher than 80%, or to use standard compounds or retention indices for identification. Please consider this issue.

**********

what does this mean?). If published, this will include your full peer review and any attached files.

If you choose “no”, your identity will remain anonymous but your review may still be made public.

**Do you want your identity to be public for this peer review?**  For information about this choice, including consent withdrawal, please see our Privacy Policy

Reviewer #3: No

Reviewer #4: No

**********

[NOTE: If reviewer comments were submitted as an attachment file, they will be attached to this email and accessible via the submission site. Please log into your account, locate the manuscript record, and check for the action link "View Attachments". If this link does not appear, there are no attachment files.]

While revising your submission, please upload your figure files to the Preflight Analysis and Conversion Engine (PACE) digital diagnostic tool, https://pacev2.apexcovantage.com/. PACE helps ensure that figures meet PLOS requirements. To use PACE, you must first register as a user. Registration is free. Then, login and navigate to the UPLOAD tab, where you will find detailed instructions on how to use the tool. If you encounter any issues or have any questions when using PACE, please email PLOS at figures@plos.org

Attachment Submitted filename: PONE-D-25-29786_reviewer.pdf

# Author comment: Author response to Decision Letter 2

**DOI:** 10.1371/journal.pone.0354454.r004

20 Nov 2025

Manuscript Title: Bioherbicidal potential of Acroptilon repens (Russian knapweed) aqueous extracts

Manuscript ID: PONE-D-25-29786R1

Journal: PLOS ONE

Corresponding Authors: Dr. Mohammad Taghi Alebrahim & Dr Dana MacGregor

Date of Resubmission:

Dear Dr. Uludag and Reviewers,

Thank you very much for your thoughtful and constructive feedback on our manuscript titled “Allelopathic potential of Acroptilon repens (Russian knapweed) aqueous extract for managing agriculturally important weed species in comparison to relevant crops” (Manuscript ID: PONE-D-25-29786R1). We appreciate the time and effort you and the reviewers have invested in evaluating our work.

We are pleased to learn that the manuscript was found to have merit, and that the majority of the reviewers’ comments were addressed in the previous draft. We have carefully considered all remaining suggestions provided by both the reviewers and the academic editor, and we have revised the manuscript accordingly to improve its clarity, scientific rigor, and overall presentation.

In the following sections, we provide a detailed point-by-point response to each comment, indicating how we have addressed the concerns raised. It is important to note that the References section now includes two additional references, numbered 55 and 56, corresponding to the newly identified compounds in Extraction Method 2. The entire reference list has been renumbered to follow the revised sequence. We hope that the revised manuscript now meets the standards required for publication in PLOS ONE.

_____

We thank Dr. Uludag for the editorial feedback and helpful suggestions to improve the clarity and presentation of our manuscript. We have addressed each point as follows:

Comment 1. Suggested Title and Running Title

• We have adopted the suggested title: “Bioherbicidal potential of Acroptilon repens (Russian knapweed) aqueous extracts”.

• The running title has been updated to: “Bioherbicidal potential of Acroptilon repens”.

2. Use of Passive and Active Voice

• We have revised the manuscript to ensure we used the passive voice where appropriate. Sentences previously written in active voice have been adjusted to passive where appropriate, particularly in the Methods and Results sections. For example:

• Original: “We assessed its impact on seed germination and early growth…” � changed to “… was assessed.”

• Original: “This study evaluates how the aqueous extract of A. repens influences germination and growth metrics of A. retroflexus, A. fatua, S. arvensis, and S. cereale compared to T. aestivum and V. faba.

3. Problematic Sentence Regarding Wheat and Corn Fields

• The sentence describing the impact of Russian knapweed on wheat and corn fields has been rephrased for clarity. The revised version avoids ambiguity and improves readability:

• Revised: “The presence of Russian knapweed in wheat and corn fields can significantly reduce yield and affect flour quality due to bitter-tasting seed contamination.”

4. Materials and Methods Opening Sentence

• As suggested, we have removed the introductory sentence from the Materials and Methods section to streamline the narrative and avoid redundancy.

5. Use of the Term “Noxious Weed”

• We have replaced the term “noxious weed” with more appropriate terminology, such as “invasive” or “problematic weed species,” to reflect ecological and agronomic relevance without implying regulatory classification.

We hope these revisions meet your expectations and contribute to a clearer and more scientifically robust manuscript.

______

Reviewer 3

We thank Reviewer #3 for their positive assessment of our manuscript and for acknowledging that all previous comments have been addressed. We appreciate the additional feedback provided and have made further revisions to improve the clarity and scientific value of the manuscript.

Comment 1: “Results are clear and understandable, except for untargeted GC-MS.”

Response: We have revised the section describing the untargeted GC-MS analysis to improve clarity and ensure that the methodology and interpretation are more accessible to readers. Specifically, we have:

• Clarified the process of extractions in the materials and methods section.

• Made it more clear that compound identifications were provisional, but that the identifications were justified based on the data we have.

• Rearranged the compound within the table so that it is clear where the confidence scores are confident and those that are putative to enhance transparency.

Comment 2: “It is however, a common study, done numerously by other researchers on other weed species.”

Response: We acknowledge that allelopathic studies using aqueous extracts have been conducted on various weed species – we refer to other studies in the paper throughout making sure that our work is put into the context of what has been done previously. We also are careful to explain why our work is unique. We believe our study is among the first to systematically compare the bioherbicidal effects of Acroptilon repens on both weed and crop species using a comprehensive set of germination and growth parameters. We have tried to make this clearer throughout.

Comment 3: “Some sentences are confusing and need to be rephrased.”

Response: We have carefully reviewed the manuscript and revised several sentences for clarity and readability. Examples include:

• Rewriting the sentence about Russian knapweed’s impact on wheat and corn fields to improve flow and precision.

• Simplifying complex sentence structures in the Introduction and Results sections.

Comment 4: “The paper would produce a better impact if study on the isolation of a target compound were to be done and presented in the manuscript.”

Response: We agree that isolating and characterizing specific bioactive compounds would enhance the impact of the study. However, this was beyond the scope of the current work, which aimed to evaluate the overall bioherbicidal potential of the aqueous extract. We have added a statement in the Discussion section acknowledging this limitation and proposing targeted compound isolation as a priority for future research.

_____

Reviewer 4

We thank Reviewer #4 for their careful review and constructive comments. We appreciate the acknowledgment that most of the previous concerns have been addressed and have made further revisions to improve the manuscript based on the remaining suggestions.

Comment 1: “In Table 3, the NIST match for some compounds is very low. It is important to select matches higher than 80%, or to use standard compounds or retention indices for identification. Please consider this issue.”

Response: We appreciate this important observation regarding compound identification. In response, we have:

• Re-evaluated the GC-MS data and removed compounds with NIST match scores below 80% unless they were supported by additional evidence (e.g., retention time consistency or literature reports).

• Added a note in the Methods section clarifying the threshold used for compound identification.

• Included a revised version of Table 3 with updated compound matches and confidence scores.

• Acknowledged the limitation of relying solely on NIST library matches and emphasized the need for future work involving standard compounds and retention indices for more definitive identification.

We hope these revisions address the reviewer’s concerns and improve the reliability of our chemical profiling.

Attachment Submitted filename: Response to Reviewers.docx

# Peer review: Decision Letter 2

**DOI:** 10.1371/journal.pone.0354454.r005

**Authors:** Ahmet Uludag

11 Dec 2025

Dear Dr. Alebrahim,

Thank you for submitting your manuscript to PLOS ONE. After careful consideration, we feel that it has merit but does not fully meet PLOS ONE’s publication criteria as it currently stands. Therefore, we invite you to submit a revised version of the manuscript that addresses the points raised during the review process.

Please fulfil requests of reviewers. If you have currently have further data requested by reviewers, please add them too.

Please submit your revised manuscript by Jan 25 2026 11:59PM. If you will need more time than this to complete your revisions, please reply to this message or contact the journal office at plosone@plos.org. When you're ready to submit your revision, log on to https://www.editorialmanager.com/pone/ and select the 'Submissions Needing Revision' folder to locate your manuscript file.

- A rebuttal letter that responds to each point raised by the academic editor and reviewer(s). You should upload this letter as a separate file labeled 'Response to Reviewers'.
- A marked-up copy of your manuscript that highlights changes made to the original version. You should upload this as a separate file labeled 'Revised Manuscript with Track Changes'.
- An unmarked version of your revised paper without tracked changes. You should upload this as a separate file labeled 'Manuscript'.

If applicable, we recommend that you deposit your laboratory protocols in protocols.io to enhance the reproducibility of your results. Protocols.io assigns your protocol its own identifier (DOI) so that it can be cited independently in the future. For instructions see: https://journals.plos.org/plosone/s/submission-guidelines#loc-laboratory-protocols. Additionally, PLOS ONE offers an option for publishing peer-reviewed Lab Protocol articles, which describe protocols hosted on protocols.io. Read more information on sharing protocols at https://plos.org/protocols?utm_medium=editorial-email&utm_source=authorletters&utm_campaign=protocols.

We look forward to receiving your revised manuscript.

Kind regards,

Ahmet Uludag, Ph.D.

Academic Editor

PLOS One

Journal Requirements:

1. If the reviewer comments include a recommendation to cite specific previously published works, please review and evaluate these publications to determine whether they are relevant and should be cited. There is no requirement to cite these works unless the editor has indicated otherwise.

2. Please review your reference list to ensure that it is complete and correct. If you have cited papers that have been retracted, please include the rationale for doing so in the manuscript text, or remove these references and replace them with relevant current references. Any changes to the reference list should be mentioned in the rebuttal letter that accompanies your revised manuscript. If you need to cite a retracted article, indicate the article’s retracted status in the References list and also include a citation and full reference for the retraction notice.

Additional Editor Comments :

Dear colleague, we always have problem to find out reviewers. I hope this time you will make necessary changes. I think further insrumental analysis is not necessary. However, please include the data if you currently have.

[Note: HTML markup is below. Please do not edit.]

Reviewers' comments:

Reviewer's Responses to Questions

**Comments to the Author**

Reviewer #5: All comments have been addressed

Reviewer #6: (No Response)

**********

2. Is the manuscript technically sound, and do the data support the conclusions?

Reviewer #5: Yes

Reviewer #6: Yes

**********

3. Has the statistical analysis been performed appropriately and rigorously?

Reviewer #5: Yes

Reviewer #6: Yes

**********

4. Have the authors made all data underlying the findings in their manuscript fully available?

The PLOS Data policy

Reviewer #5: Yes

Reviewer #6: (No Response)

**********

5. Is the manuscript presented in an intelligible fashion and written in standard English?

Reviewer #5: Yes

Reviewer #6: (No Response)

**********

Reviewer #5: Subject: Comments and Observations on Manuscript [Bioherbicidal potential of Acroptilon repens L. (Russian knapweed) aqueous extracts]

Dear Editor’s,

After a thorough review of the submitted manuscript titled (Bioherbicidal potential of Acroptilon repens L. (Russian knapweed) aqueous extracts),we would like to bring to your attention several observations and points that may require clarification or revision. These observations are summarized below under scientific, methodological, linguistic, and reference-related aspects:

1. Scientific / Methodological Issues

• Experimental Design:

o The rationale for selecting the concentrations (0–200 g L⁻¹) was not explained, nor was it indicated whether these levels are field-applicable.

o Inconsistencies in reporting the number of seeds per replicate (60 seeds mentioned in some sections, not in others) need correction.

o Environmental control was limited to temperature; humidity and ventilation in the incubator were not mentioned.

• Statistical Analysis:

o Only LSD at 5% was reported, without verification of ANOVA assumptions such as variance homogeneity.

o Pearson correlation was applied to all traits, which may be inappropriate if some data are non-normal or highly heterogeneous.

• Measurement of Indicators:

o Some indices (GP, GR, GI, PV, GV) are complex and repetitive; simplification or merging is recommended.

o Discrepancies exist between text and tables (e.g., MGT), sometimes stating no significant differences, sometimes reporting large increases.

• Result Interpretation:

o Statements generalizing sensitivity of invasive species are based on limited data.

o Lack of negative controls or sufficient replicates to assess natural variation.

o Claims about “biological effects” from GC-MS are speculative, based solely on compound presence without experimental validation.

2. Linguistic / Stylistic Issues

• Repetition of species names within the same sentence.

• Very long and complex sentences that reduce readability.

• Use of technical terms without definition (e.g., PV).

• Spelling errors (e.g., “inhabitation” → inhibition, “Secale cereal” → Secale cereale, “thefinal” → the final).

3. References

• Some references are repeated or inconsistent; order should be corrected.

• Some references are outdated (pre-2000), with no justification for relevance.

• Figure and citation inconsistencies (e.g., Fig 1d vs. Fig 1 e).

4. Additional Observations

• No comparison with field studies; all results are laboratory-based.

• Use of the term “bioherbicide” without toxicity or environmental assessment.

• Study limitations, such as differences between fresh and stored seeds, were not stated.

• Some tables (Tables 1–4) were not included, complicating result verification.

• Occasional grammatical inconsistencies, especially with commas and parentheses.

Recommendations for Improvement:

• Correct all scientific and spelling errors, including species names.

• Simplify complex sentences and indicators.

• Clarify rationale for extraction concentrations and field applicability.

• Interpret results cautiously without overgeneralization.

• Review references for consistency and relevance.

• Explicitly mention study limitations and potential practical constraints.

• Indicate that GC-MS findings are speculative unless experimentally validated.

We hope these comments will help improve the clarity, accuracy, and scientific rigor of the manuscript.

Thank you for your attention.

Sincerely,

Reviewer #6: The manuscript Bioherbicidal potential of Acroptilon repens L. (Russian knapweed) aqueous extracts is very interesting. However, it is not clear to me why GC–MS was used for the characterization of the extract instead of HPLC. Please clarify. Additionally, is it possible to perform an HPLC analysis to better characterize the chemical composition of the extract?

Furthermore, in Tables 3 and 4 only the detected substances are listed, without their relative abundance. The representation (quantitative share) of these components is very important.

**********

what does this mean?). If published, this will include your full peer review and any attached files.

If you choose “no”, your identity will remain anonymous but your review may still be made public.

**Do you want your identity to be public for this peer review?**  For information about this choice, including consent withdrawal, please see our Privacy Policy

Reviewer #5: No

Reviewer #6: No

**********

[NOTE: If reviewer comments were submitted as an attachment file, they will be attached to this email and accessible via the submission site. Please log into your account, locate the manuscript record, and check for the action link "View Attachments". If this link does not appear, there are no attachment files.]

To ensure your figures meet our technical requirements, please review our figure guidelines: https://journals.plos.org/plosone/s/figures

You may also use PLOS’s free figure tool, NAAS, to help you prepare publication quality figures: https://journals.plos.org/plosone/s/figures#loc-tools-for-figure-preparation.

NAAS will assess whether your figures meet our technical requirements by comparing each figure against our figure specifications.

# Author comment: Author response to Decision Letter 3

**DOI:** 10.1371/journal.pone.0354454.r006

4 Feb 2026

Response to Reviewers

General comment: We thank the reviewers for their constructive feedback. Below are our detailed responses to each comment.

Reviewer #5:

Subject:

Comments and Observations on Manuscript [Bioherbicidal potential of Acroptilon repens L. (Russian knapweed) aqueous extracts]

Dear Editor’s,

After a thorough review of the submitted manuscript titled (Bioherbicidal potential of Acroptilon repens L. (Russian knapweed) aqueous extracts), we would like to bring to your attention several observations and points that may require clarification or revision. These observations are summarized below under scientific, methodological, linguistic, and reference-related aspects:

1. Scientific / Methodological Issues

• Experimental Design:

o The rationale for selecting the concentrations (0–200 g L⁻¹) was not explained, nor was it indicated whether these levels are field-applicable.

- Response: Thank you for raising this point. The concentrations (0 to 200 g L⁻¹) were selected to cover a broad range for initial screening purposes. This study aimed to first and foremost establish a baseline understanding of whether the extract would be effective; therefore, considerations regarding field applicability were outside the scope of this experiment.

o Inconsistencies in reporting the number of seeds per replicate (60 seeds mentioned in some sections, not in others) need correction.

- Response: We appreciate your observation. We have changed the text to make it clearer that three technical replicates of 20 seeds per replicate were used and that the total seeds per analysis was 60. The number of seeds per replicate has been standardized and made consistent throughout the manuscript.

o Environmental control was limited to temperature; humidity and ventilation in the incubator were not mentioned.

- Response: Thank you for noting this. The plates containing the seeds were sealed and therefore humidity and ventilation details of the chamber itself did not affect the conditions that the seeds were experiencing. We have added text that clarifies this and highlighted that the aim was to ensure consistent and equivalent conditions between the dishes.

• Statistical Analysis:

o Only LSD at 5% was reported, without verification of ANOVA assumptions such as variance homogeneity.

o Pearson correlation was applied to all traits, which may be inappropriate if some data are non-normal or highly heterogeneous.

� Response: We acknowledge your point. ANOVA assumptions were checked, and this is now stated in the revised manuscript. Data normality was assessed using the Shapiro–Wilk test, and non-normal data were normalized using a square root transformation. Homogeneity of variances was verified using Levene’s test. After confirming these assumptions, ANOVA was performed, and mean comparisons were conducted using LSD at 5% significance. Pearson correlation: Pearson correlation was applied only to traits that met the assumptions of normality and homogeneity of variance. For traits that did not meet these assumptions, appropriate square root transformations were applied prior to correlation analysis to ensure valid results.

• Measurement of Indicators:

o Some indices (GP, GR, GI, PV, GV) are complex and repetitive; simplification or merging is recommended.

� Response: Thank you for your advice. The data regarding the indices you highlight are in the Supplementary Data section. Although perhaps complex and repetitive, they are quantitative, robust measurements for different seed germination or seedling growth parameters and may prove to be useful for other researchers who would like to do future studies. The main data in this manuscript includes only the key parameters of GP, GR, MGT, and the length of the shoot and root of the seedling. We argue that simplification or merging would result in the loss of useful data.

o Discrepancies exist between text and tables (e.g., MGT), sometimes stating no significant differences, sometimes reporting large increases.

- Response: We have simplified the presentation of indices and ensured consistency between text and tables. We also moved the mean comparison table from the Supplementary Data section to the main part of the manuscript to resolve any ambiguities created based on Figure 1 (relative data).

• Result Interpretation:

o Statements generalizing sensitivity of invasive species are based on limited data.

- Response: Many reports on the bioactivity of extracts report few or limited data. We have tested three weedy species (Sinapis arvensis L., Amaranthus retroflexus L., and Avena fatua L.), a multipurpose species (Secale cereale L.), and two crop species (Triticum aestivum L. and Vicia faba L.), in appropriate technical replicates. Although the dataset is relatively limited, we consider it useful and comparable to, or more comprehensive than, datasets reported in previous studies.

o Lack of negative controls or sufficient replicates to assess natural variation.

� Response: Negative controls, where no extract was added, are included for all germination parameters. We agree that we have not assessed the potential range of responses that may be occurring across the natural variation of the target species; however, the main aim of this paper is to establish that application of A. repens extract can have an effect across a range of representative species. Therefore, analysis of natural variation either within different sources of A. repens as the extract source, or of the target species themselves, was outside the scope of this work. We aimed to establish a baseline for whether the extract had activity and on which species, rather than how effective it is under field conditions.

o Claims about “biological effects” from GC-MS are speculative, based solely on compound presence without experimental validation.

- Response: We agree that GC-MS findings are indicative and not confirmatory. We have repeatedly used the words "putatively" and "Putative" in various sections of the GC-MS results and discussion to indicate that the GC-MS results are speculative. However, we explicitly mentioned in the final section of the discussion that future studies require compound isolation and experimental validation.

2. Linguistic / Stylistic Issues

• Repetition of species names within the same sentence.

- Response: Because we are using multiple species, we aimed for clarity. The repetition of species names in one sentence is more likely due to differences in A. repens concentrations in inhibiting germination or their different sensitivities.

Such as “Germination was fully inhibited for S. arvensis ≥ 40 g L⁻¹, A. retroflexus ≥ 80 g L⁻¹, A. fatua ≥ 120 g L⁻¹, T. aestivum ≥ 140 g L⁻¹, S. cereale ≥ 160 g L⁻¹, and V. faba ≥ 200 g L⁻¹” or “Radicle lengths varied greatly between the species. The longest radicle length belonged to A. fatua > S. arvensis > V. faba, respectively. Secale cereale, and T. aestivum had similar radicle length.”

In other cases, the phrase "all tested species" was used.

• Very long and complex sentences that reduce readability.

- Response: The manuscript has been carefully revised to improve readability and clarity. Additionally, it was further refined for language and grammatical accuracy using the Springer Nature language editing service.

• Use of technical terms without definition (e.g., PV).

- Response: PV (Peak Value) was defined in the Materials and Methods section on line 336 (Revised Manuscript with track changes). All abbreviations related to tables are also provided in the form of captions.

• Spelling errors (e.g., “inhabitation” → inhibition, “Secale cereal” → Secale cereale, “thefinal” → the final).

- Response: All noted linguistic and spelling errors have been corrected.

3. References

• Some references are repeated or inconsistent; order should be corrected.

- Response: We have edited the reference list to ensure there are no duplicate references in the reference list. In some cases, several publications from the same author in different years have been used, but they are not duplicates. Also, due to the limited references on the allelopathic compounds of A. repens, we have had to use some articles that are clearly not similar to our work with the aim of making sure no unrelated references are used. The referencing has been standardized as appropriate for the journal.

• Some references are outdated (pre-2000), with no justification for relevance.

- Response: As we answered in the previous question, due to the limited resources on the allelopathic compounds of A. repens and the lack of a similar article to our manuscript, we were forced to use some relatively similar sources that have previously addressed the identification of allelopathic compounds. However, we would argue that simply being an older reference does not make it irrelevant.

• Figure and citation inconsistencies (e.g., Fig 1d vs. Fig 1 e).

- Response: We appreciate the careful observation of the reviewer and have worked to ensure any differences in figure citations have been corrected.

4. Additional Observations

• No comparison with field studies; all results are laboratory-based.

- Response: We clarify that the present study was intentionally designed as a controlled laboratory experiment with the primary objective of establishing a fundamental biological baseline for the allelopathic activity of Acroptilon repens extract. At this exploratory stage, the focus is on determining whether the extract exhibits measurable biological effects under standardized conditions, rather than on assessing agronomic performance. Comparisons with field-based studies were therefore not included, as such comparisons would not be methodologically appropriate at this phase of investigation. Field experiments require prior evidence of biological efficacy, as well as optimization of formulation, dose, and application methods, which have not yet been established for A. repens extract. Furthermore, as noted in the manuscript, extrapolation between different extract types or between laboratory and field conditions should be approached with caution, given substantial differences in chemical composition, bioavailability, and interactions with environmental factors. Consequently, the laboratory-based approach adopted here represents a necessary and scientifically justified first step toward future applied or field-oriented research.

• Use of the term “bioherbicide” without toxicity or environmental assessment.

- Response: Thank you for this point. We agree with you that the term "bioherbicides" cannot be used without a toxicity assessment. We have revised the two sentences that we had clearly mentioned.

“Although comprehensive ecological and agronomic assessments are needed prior to field application and further research is needed to isolate and confirm the bioactivity of these potentially phytotoxic secondary metabolites, these data support the potential use of A. repens aqueous extract as a natural, broad-spectrum allopathic agent, particularly as it is more active against the weeds than the crops we tested herein.”

“However, these data demonstrate that A. repens extract has the potential to be used as an allelopathic germination inhibitor.”

• Study limitations, such as differences between fresh and stored seeds, were not stated.

- Response: We have added further clarity about the collection or acquisition and storage of seeds into the Materials and Methods section. We have also added text to the Discussion explicitly acknowledging 1) that the two classes of plants (weeds and crops) were from seeds that were acquired or purchased and therefore had different storage histories, and 2) although they showed high germination without A. repens extract, that these differences may have affected their responses and therefore contributed to the distinct sensitivity patterns observed between weedy and cultivated species. We have also noted that future work should use seeds with equivalent age, storage conditions, and vigour to better isolate biological responses from seed‑handling effects.

• Some tables (Tables 1–4) were not included, complicating result verification.

- Response: We ensured that all tables were included in the text and in the upload. In this manuscript, we presented five tables, including the results of the analysis of variance, comparison of means, linear slope, and Pearson’s R2 values, and tables related to the GC-MS results. The other tables are related to supplementary data, which have been made available to PLOS One Journal in separate files.

• Occasional grammatical inconsistencies, especially with commas and parentheses.

- Response: Thank you for reading the manuscript very carefully. We have double-checked this text with our native English-speaking author and used the Springer Nature editor to correct the article's readability and grammatical errors. If further errors are found, we are happy to correct them in the typesetting and final editing stages.

Recommendations for Improvement:

• Correct all scientific and spelling errors, including species names.

• Simplify complex sentences and indicators.

• Clarify rationale for extraction concentrations and field applicability.

• Interpret results cautiously without overgeneralization.

• Review references for consistency and relevance.

• Explicitly mention study limitations and potential practical constraints.

• Indicate that GC-MS findings are speculative unless experimentally validated.

- Response: We appreciate that these comments have helped improve the clarity, accuracy, and scientific rigor of the manuscript. We feel we have addressed each of these points above with further detail.

Reviewer #6:

The manuscript Bioherbicidal potential of Acroptilon repens L. (Russian knapweed) aqueous extracts is very interesting. However, it is not clear to me why GC–MS was used for the characterization of the extract instead of HPLC. Please clarify. Additionally, is it possible to perform an HPLC analysis to better characterize the chemical composition of the extract?

- Response: Thank you for this suggestion. Untargeted GC-MS was chosen because it is well-suited for volatile and semi-volatile compounds present in the extract. While HPLC could provide complementary information, it was beyond the scope of this initial screening study. We will keep that in mind if we can obtain further funding to conduct follow-up studies on this promising extract.

Furthermore, in Tables 3 and 4 only the detected substances are listed, without their relative abundance. The representation (quantitative share) of these components is very important.

- Response: We appreciate this observation. Unfortunately, we were unable to generate absolute measures for the putatively identified compounds; our untargeted analysis was performed without compound specific calibration. We used an Agilent 7890B GC coupled to a 5977A MSD. The software computes Area (%) values which represent the relative abundance of detected compounds based on their integrated peak areas in the GC–MS chromatogram response rather than absolute concentrations. We have added this text into the Materials and methods, Results and Discussions explaining this and highlighting this as a limitation of our study.

Attachment Submitted filename: Response_to_Reviewers_auresp_3.docx

# Peer review: Decision Letter 3

**DOI:** 10.1371/journal.pone.0354454.r007

**Authors:** Ahmet Uludag

30 Apr 2026

Dear Dr. Alebrahim,

Please submit your revised manuscript by Jun 14 2026 11:59PM. If you will need more time than this to complete your revisions, please reply to this message or contact the journal office at plosone@plos.org. When you're ready to submit your revision, log on to https://www.editorialmanager.com/pone/ and select the 'Submissions Needing Revision' folder to locate your manuscript file.

- A letter that responds to each point raised by the academic editor and reviewer(s). You should upload this letter as a separate file labeled 'Response to Reviewers'.
- A marked-up copy of your manuscript that highlights changes made to the original version. You should upload this as a separate file labeled 'Revised Manuscript with Track Changes'.
- An unmarked version of your revised paper without tracked changes. You should upload this as a separate file labeled 'Manuscript'.

If you would like to make changes to your financial disclosure, please include your updated statement in your cover letter. Guidelines for resubmitting your figure files are available below the reviewer comments at the end of this letter.

If applicable, we recommend that you deposit your laboratory protocols in protocols.io to enhance the reproducibility of your results. Protocols.io assigns your protocol its own identifier (DOI) so that it can be cited independently in the future. For instructions see: https://journals.plos.org/plosone/s/submission-guidelines#loc-laboratory-protocols. Additionally, PLOS ONE offers an option for publishing peer-reviewed Lab Protocol articles, which describe protocols hosted on protocols.io. Read more information on sharing protocols at https://plos.org/protocols?utm_medium=editorial-email&utm_source=authorletters&utm_campaign=protocols.

As the corresponding author, your ORCID iD is verified in the submission system and will appear in the published article. PLOS supports the use of ORCID, and we encourage all coauthors to register for an ORCID iD and use it as well. Please encourage your coauthors to verify their ORCID iD within the submission system before final acceptance, as unverified ORCID iDs will not appear in the published article. *Only*  the individual author can complete the verification step; PLOS staff *cannot*  verify ORCID iDs on behalf of authors.

We look forward to receiving your revised manuscript.

Kind regards,

Ahmet Uludag, Ph.D.

Academic Editor

PLOS One

Journal Requirements:

1. If the reviewer comments include a recommendation to cite specific previously published works, please review and evaluate these publications to determine whether they are relevant and should be cited. There is no requirement to cite these works unless the editor has indicated otherwise.

2. Please review your reference list to ensure that it is complete and correct. If you have cited papers that have been retracted, please include the rationale for doing so in the manuscript text, or remove these references and replace them with relevant current references. Any changes to the reference list should be mentioned in the rebuttal letter that accompanies your revised manuscript. If you need to cite a retracted article, indicate the article’s retracted status in the References list and also include a citation and full reference for the retraction notice.

Additional Editor Comments:

I got different views from the reviewers. One of the reviewers accepted wihle the other rejected. I think he pointed out some good points to improve this paper. I have fully agreed some of his statements, see below:

"the manuscript repeatedly refers to "bioherbicidal potential", "allelopathic effects", and "broad-spectrum allelopathic agent". However, the experimental design is limited to in vitro germination and seedling growth assays, which demonstrate phytotoxic effects under controlled conditions, not allelopathy in an ecological sense. No evidence is provided regarding release into soil, persistence, or plant–plant interactions. Therefore, the use of the term "bioherbicide" in the title and the interpretation in terms of allelopathy are not adequately supported and should be reconsidered".

In addition, could you please see some other literature as he mentioned. Explain your procedure clearly and discuss methodological difference with earlier literature. Discuss and connect clearly your bio data with GCMS data.

[Note: HTML markup is below. Please do not edit.]

Reviewers' comments:

Reviewer's Responses to Questions

**Comments to the Author**

Reviewer #7: All comments have been addressed

Reviewer #8: (No Response)

**********

2. Is the manuscript technically sound, and do the data support the conclusions?

Reviewer #7: Yes

Reviewer #8: No

**********

3. Has the statistical analysis been performed appropriately and rigorously?

Reviewer #7: Yes

Reviewer #8: Yes

**********

4. Have the authors made all data underlying the findings in their manuscript fully available?

The PLOS Data policy

Reviewer #7: Yes

Reviewer #8: No

**********

5. Is the manuscript presented in an intelligible fashion and written in standard English?

Reviewer #7: Yes

Reviewer #8: Yes

**********

Reviewer #7: Dear Authors

Thank you for submitting the revised version of your paper. You have answered all the questions of the reviewers in appropriate way. Your responses are very satisfactory and I am fully agreed with your responses which you have also incorporated in the revised version of your manuscript in appropriate places.

Reviewer #8: The manuscript entitled "Bioherbicidal potential of Acroptilon repens L. aqueous extracts" investigates the effects of aqueous extracts on seed germination and early seedling growth of several plant species.

While the study presents a dataset that is, in principle, technically usable, the work is affected by substantial conceptual, methodological, and interpretative limitations. In particular, it suffers from overstatement of the biological relevance of the results, insufficient integration with the existing literature, inconsistencies in the extraction procedures, and limited and weakly supported chemical characterization.

First, the manuscript repeatedly refers to "bioherbicidal potential", "allelopathic effects", and "broad-spectrum allelopathic agent". However, the experimental design is limited to in vitro germination and seedling growth assays, which demonstrate phytotoxic effects under controlled conditions, not allelopathy in an ecological sense. No evidence is provided regarding release into soil, persistence, or plant–plant interactions. Therefore, the use of the term "bioherbicide" in the title and the interpretation in terms of allelopathy are not adequately supported and should be reconsidered.

Second, the manuscript does not adequately engage with the existing literature on A. repens. Several relevant studies on aqueous extracts, powdered plant material, essential oils, and ecological interactions are not sufficiently discussed. As a result, the work is not properly contextualized and appears largely confirmatory rather than novel.

Third, the extraction procedure used for the bioassays raises concerns regarding reproducibility. Different concentrations (0–200 g/L) are prepared independently rather than obtained from a single standardized extract. This approach does not ensure consistent extraction efficiency or comparable chemical composition across treatments and introduces methodological variability that is not discussed.

A major limitation concerns the lack of correspondence between the extracts used in biological assays and those analyzed by GC–MS. The GC–MS analysis is performed on extracts prepared using a different procedure (1:10 w/v extraction with additional processing steps), which does not reflect the extracts used in the bioassays. Consequently, the reported chemical composition cannot be directly linked to the observed biological effects, and the interpretation relating specific compounds to activity is not sufficiently supported.

In addition, the chemical characterization is limited by the use of GC–MS alone, which does not capture the full range of compounds present in aqueous extracts, particularly polar constituents. Compound identification is putative and based on library matching, while the reported relative abundances (area %) do not provide quantitative information. Therefore, linking specific compounds to the observed biological activity is not sufficiently supported.

The description of sample processing (drying followed by lyophilization) is also unclear and requires clarification, as the rationale for combining these steps is not adequately explained.

The M&M section would benefit from improved clarity and detail. In particular, the origin of seeds is insufficiently documented (supplier and batch information are not provided), and storage conditions are described in vague terms. Some methodological descriptions are redundant or not optimally structured.

The Results section is largely descriptive and, in several cases, lacks sufficient quantitative interpretation. The organization by individual indices leads to fragmentation, and some analyses (e.g., correlations) appear of limited relevance.

The Discussion largely reiterates the results and lacks critical comparison with previous studies. It also includes speculative links between detected compounds and biological activity that are not adequately supported by the data.

Finally, the Conclusions section reflects the same tendency toward overinterpretation and would benefit from a more cautious alignment with the actual scope of the experimental evidence.

**********

what does this mean?). If published, this will include your full peer review and any attached files.

If you choose “no”, your identity will remain anonymous but your review may still be made public.

**Do you want your identity to be public for this peer review?**  For information about this choice, including consent withdrawal, please see our Privacy Policy

Reviewer #7: No

Reviewer #8: No

**********

[NOTE: If reviewer comments were submitted as an attachment file, they will be attached to this email and accessible via the submission site. Please log into your account, locate the manuscript record, and check for the action link "View Attachments". If this link does not appear, there are no attachment files.]

To ensure your figures meet our technical requirements, please review our figure guidelines: https://journals.plos.org/plosone/s/figures

You may also use PLOS’s free figure tool, NAAS, to help you prepare publication quality figures: https://journals.plos.org/plosone/s/figures#loc-tools-for-figure-preparation.

NAAS will assess whether your figures meet our technical requirements by comparing each figure against our figure specifications.

# Author comment: Author response to Decision Letter 4

**DOI:** 10.1371/journal.pone.0354454.r008

9 Jun 2026

PONE-D-25-29786R3

Bioherbicidal potential of Acroptilon repens L. (Russian knapweed) aqueous extracts

Reviewer #7s' comment: Thank you for submitting the revised version of your paper. You have answered all the questions of the reviewers in appropriate way. Your responses are very satisfactory and I am fully agreed with your responses which you have also incorporated in the revised version of your manuscript in appropriate places.

Authors: We thank Review #7 for their careful reading of our manuscript and are happy to hear that they were satisfied that all comments have been addressed and the manuscript is ready to be accepted.

Additional Editor Comments:

Editor: I got different views from the reviewers. One of the reviewers accepted wihle the other rejected. I think he pointed out some good points to improve this paper. I have fully agreed some of his statements, see below:

the manuscript repeatedly refers to "bioherbicidal potential", "allelopathic effects", and "broad-spectrum allelopathic agent". However, the experimental design is limited to in vitro germination and seedling growth assays, which demonstrate phytotoxic effects under controlled conditions, not allelopathy in an ecological sense. No evidence is provided regarding release into soil, persistence, or plant–plant interactions. Therefore, the use of the term "bioherbicide" in the title and the interpretation in terms of allelopathy are not adequately supported and should be reconsidered".

AUTHORS: We thank the Academic Editor and reviewers for their careful and constructive evaluation of our manuscript. In particular, we have addressed the conceptual concerns raised regarding the interpretation of our results by substantially revising the title, abstract, discussion, and conclusions to clearly delimit the scope of the study. Although Acroptilon repens is well established in the literature as an allelopathic species, we now explicitly distinguish between previously reported allelopathic behaviour and the in vitro phytotoxic effects assessed here. All terminology implying ecological allelopathy or bioherbicidal activity unsupported by the experimental design has been removed or qualified, and methodological limitations are now clearly acknowledged. We believe these revisions ensure that the manuscript accurately reflects the data presented, is appropriately contextualized within the existing literature, and aligns fully with the reviewer and editorial guidance.

Editor: In addition, could you please see some other literature as he mentioned.

AUTHORS: We have added additional literature references, particularly in the introduction to highlight the considerable amount of research that has established A. repens as an allelopathic plant with demonstrable ability to repress seedling germination and establishment. We have also added in text that highlights how our study differs from this body of literature and what aspects of it are unique and novel.

Editor: Explain your procedure clearly and discuss methodological difference with earlier literature.

AUTHORS: We have tightened the methodology sections of the paper and clarified how our protocols match and differ from earlier literature.

Editor: Discuss and connect clearly your bio data with GCMS data.

AUTHORS: We have added in additional text to discuss and connect our biological data with the GC-MS data. As there is considerable data about the allelopathic metabolites within and released by A. repens, we have reframed this section to be exploratory data that are confirming the previous findings rather than conclusive evidence of what compounds are causing the observed effects. We have also streamlined the discussion text so that it is focused on what is probably active and what is probably just structural components.

Reviewer #8's Responses to Questions 3. Has the statistical analysis been performed appropriately and rigorously?

Reviewer #8: Yes

Reviewer #8's Response to Question 5. Is the manuscript presented in an intelligible fashion and written in standard English?

Reviewer #8: Yes

AUTHORS: We appreciate that Reviewer #8 recognizes that our paper is statistically sound and written in intelligible fashion in standard English.

________________________________________

2. Is the manuscript technically sound, and do the data support the conclusions? Reviewer #8: No

AUTHORS: As evidenced in the tracked changes, we have altered the manuscript to make it more technically sound, ensuring that all the conclusions are supported by the data.

4. Have the authors made all data underlying the findings in their manuscript fully available? Reviewer #8: No

AUTHORS: We have ensured that all of the data are available by effectively using the tables and figures within the paper, adding in supplementary files where appropriate.

________________________________________

6. Review Comments to the Author

Reviewer #8: The manuscript entitled "Bioherbicidal potential of Acroptilon repens L. aqueous extracts" investigates the effects of aqueous extracts on seed germination and early seedling growth of several plant species.

While the study presents a dataset that is, in principle, technically usable, the work is affected by substantial conceptual, methodological, and interpretative limitations. In particular, it suffers from overstatement of the biological relevance of the results, insufficient integration with the existing literature, inconsistencies in the extraction procedures, and limited and weakly supported chemical characterization.

AUTHORS: We thank the reviewer for this detailed assessment. We agree that earlier versions of the manuscript overstated the biological relevance of the findings, did not sufficiently contextualize the work within the existing literature, and did not adequately acknowledge methodological and interpretative limitations. In response, we have substantially revised the manuscript to address each of these concerns, as detailed below.

Reviewer #8: First, the manuscript repeatedly refers to "bioherbicidal potential", "allelopathic effects", and "broad-spectrum allelopathic agent". However, the experimental design is limited to in vitro germination and seedling growth assays, which demonstrate phytotoxic effects under controlled conditions, not allelopathy in an ecological sense. No evidence is provided regarding release into soil, persistence, or plant–plant interactions. Therefore, the use of the term "bioherbicide" in the title and the interpretation in terms of allelopathy are not adequately supported and should be reconsidered.

AUTHORS: We agree with the reviewer that the experimental design employed in this study, e.g. in vitro germination and seedling growth assays, does not directly test allelopathic interactions in an ecological or soil mediated context. We have highlighted this limitation in the new version of the text. At the same time, we note that Acroptilon repens has been widely described as an allelopathic species in the literature, based on a long history of observations from field and soil based assays [Fletcher & Renney, 1963] and considerable in vitro assays as detailed in the text. To avoid conflating these established properties with the specific scope of our experiments, we have revised the manuscript to clearly distinguish between previously reported allelopathic behaviour of A. repens and the in vitro phytotoxic effects demonstrated here. Accordingly, the term “bioherbicide” has been removed from the title, which has been altered to better reflect the findings of the paper, and references to allelopathy have been restricted to background context, while interpretation of our results is now limited to inhibitory or phytotoxic effects observed under controlled conditions.

Reviewer #8: Second, the manuscript does not adequately engage with the existing literature on A. repens. Several relevant studies on aqueous extracts, powdered plant material, essential oils, and ecological interactions are not sufficiently discussed. As a result, the work is not properly contextualized and appears largely confirmatory rather than novel.

AUTHORS: We thank the reviewer for this comment. We acknowledge that the version read contains revisions, made in response to prior reviewer guidance, that substantially reduced discussion of the allelopathic literature on Acroptilon repens. This was done with the aim of avoiding conflating previous work with the scope of our in vitro assays. In the current revision, we have expanded text that discusses relevant A. repens studies. For instance, we have expanded the Introduction to better contextualize the study within the existing literature on A. repens, explicitly summarising prior work on aqueous extracts and germination responses. This revised framing situates the present results within the existing literature while maintaining a clear distinction between previously reported allelopathic behaviour and the specific phytotoxic effects demonstrated here.

Reviewer #8: Third, the extraction procedure used for the bioassays raises concerns regarding reproducibility. Different concentrations (0–200 g/L) are prepared independently rather than obtained from a single standardized extract. This approach does not ensure consistent extraction efficiency or comparable chemical composition across treatments and introduces methodological variability that is not discussed.

AUTHORS: We thank the reviewer for raising this point. We would like to clarify that all extract concentrations were prepared from a single, pooled, and homogenized bulk sample of dried A. repens material, rather than from independently sourced plant material. Different concentrations were generated by varying the mass of this same bulk powder relative to a fixed volume of distilled water, thereby maintaining a consistent plant source across treatments. We recognize that this was not sufficiently explicit in the previous version of the Methods section and have revised the text to clearly state the use of a single homogenized batch. We have also added clarification regarding the implications of this extraction approach for reproducibility and interpretation in the Discussion.

Reviewer #8: A major limitation concerns the lack of correspondence between the extracts used in biological assays and those analysed by GC–MS. The GC–MS analysis is performed on extracts prepared using a different procedure (1:10 w/v extraction with additional processing steps), which does not reflect the extracts used in the bioassays. Consequently, the reported chemical composition cannot be directly linked to the observed biological effects, and the interpretation relating specific compounds to activity is not sufficiently supported.

In addition, the chemical characterization is limited using GC–MS alone, which does not capture the full range of compounds present in aqueous extracts, particularly polar constituents. Compound identification is putative and based on library matching, while the reported relative abundances (area %) do not provide quantitative information. Therefore, linking specific compounds to the observed biological activity is not sufficiently supported.

AUTHORS: We thank the reviewer for their view on this matter. We agree that GC–MS alone does not capture the full chemical complexity of aqueous extracts, particularly polar constituents, and that compound identification based on library matching is necessarily putative. In response, we have dramatically revised the manuscript to explicitly present the GC–MS results as qualitative and hypothesis generating rather than quantitative or causal. We now clarify that relative peak areas do not provide absolute concentrations and that no direct linkage between specific compounds and observed biological effects can be inferred from the present data.

At the same time, we note that Acroptilon repens has been extensively studied in the literature using complementary approaches, and the compounds detected here are consistent with classes of phytotoxic and allelopathic metabolites previously reported for this species. We have revised the Discussion to use the existing literature for contextualisation rather than attribution of causality, and we explicitly acknowledge that targeted chemical analyses and bioactivity guided fractionation would be required to establish mechanistic links.

In response to this comment, we have revised the manuscript to remove data from the “method 1“ extraction, and clarify how the aqueous extract was generated in the same way as those used for biological activity with the exception of the steps needed to convert the aqueous extract to something suitable for GC injection and that they were 10:1 w/v (not 1:10 w/v as noted above), clearly state the limitations of GC–MS for aqueous extracts, present compound identification as “putative“ and “non quantitative“, and restrict discussion of detected metabolites to contextualization within the existing literature rather than attribution of causal biological activity.

We have also ensured that the peaks that were identified have high NSIT matches and were not probable contaminations from the extraction method itself. We have altered the discussion appropriately.

Reviewer #8: The description of sample processing (drying followed by lyophilization) is also unclear and requires clarification, as the rationale for combining these steps is not adequately explained.

AUTHORS: We thank the reviewer for raising this concern and recognize that the description of the extraction procedure in the previous version did not sufficiently emphasize the steps taken to ensure reproducibility. As we state above, all bioassay and GC–MS extractions were prepared from a single pooled, homogenized bulk sample of dried Acroptilon repens plant material, which was finely ground prior to extraction. We have revised the Methods section to explicitly distinguish between air drying of plant material prior to generating the aqueous extract and post extraction drying of aqueous extracts for GC-MS analysis. Air drying was used to stabilize plant tissue and ensure consistent mass to volume ratios before extraction, whereas subsequent drying and lyophilization of the liquid extracts were performed to concentrate aqueous extracts and obtain solvent free residues suitable for GC–MS analysis. The rationale for this two step approach is now clearly explained in the revised manuscript.

Reviewer #8: The M&M section would benefit from improved clarity and detail. In particular, the origin of seeds is insufficiently documented (supplier and batch information are not provided), and storage conditions are described in vague terms. Some methodological descriptions are redundant or not optimally structured.

AUTHORS: We thank the reviewer for this constructive comment. The Materials and Methods section has been revised to improve clarity, reproducibility, and structure. Specifically, we have expanded the description of seed origin to include supplier details and batch information for commercially sourced material, and the location, collection timing, and post-harvest handling of field-collected seeds. Storage conditions are now described explicitly, including temperature, light exposure, and storage duration. In addition, redundant methodological descriptions have been removed and related procedures consolidated into clearly structured sections to improve readability. We have also clarified the distinction between extracts used for biological assays and those prepared for GC–MS analysis to ensure accurate interpretation of the chemical data. These revisions ensure that the methodology is fully transparent and reproducible.

Reviewer #8: The Results section is largely descriptive and, in several cases, lacks sufficient quantitative interpretation. The organization by individual indices leads to fragmentation, and some analyses (e.g., correlations) appear of limited relevance.

AUTHORS: We thank the reviewer for this helpful comment. The Results section has been considerably revised to improve clarity, quantitative interpretation, and overall structure. Specifically, we have reorganized the presentation to focus on biologically relevant patterns (e.g., overall concentration response, weed vs crop comparison, and species-specific sensitivity) rather than r

Attachment Submitted filename: Response_to_reviewers_auresp_4.docx

# Peer review: Decision Letter 4

**DOI:** 10.1371/journal.pone.0354454.r009

**Authors:** Ahmet Uludag

8 Jul 2026

Under controlled conditions, lower concentrations of aqueous extracts of Acroptilon repens are needed to inhibit seed germination and seedling growth of weeds than crops

PONE-D-25-29786R4

Dear Dr. Alebrahim,

We’re pleased to inform you that your manuscript has been judged scientifically suitable for publication and will be formally accepted for publication once it meets all outstanding technical requirements.

Within one week, you’ll receive an e-mail detailing the required amendments. When these have been addressed, you’ll receive a formal acceptance letter and your manuscript will be scheduled for publication.

An invoice will be generated when your article is formally accepted. Please note, if your institution has a publishing partnership with PLOS and your article meets the relevant criteria, all or part of your publication costs will be covered. Please make sure your user information is up-to-date by logging into Editorial Manager at Editorial Manager® and clicking the ‘Update My Information' link at the top of the page. For questions related to billing, please contact billing support.

If your institution or institutions have a press office, please notify them about your upcoming paper to help maximize its impact. If they’ll be preparing press materials, please inform our press team as soon as possible -- no later than 48 hours after receiving the formal acceptance. Your manuscript will remain under strict press embargo until 2 pm Eastern Time on the date of publication. For more information, please contact onepress@plos.org.

Kind regards,

Ahmet Uludag, Ph.D.

Academic Editor

PLOS One

Additional Editor Comments (optional):

Congratulations. I am aorry too long to complete this process. It is not wasy to find reviewers. Furthermore, your manuscript canged much during the process.

Reviewers' comments:

Reviewer's Responses to Questions

**Comments to the Author**

Reviewer #4: (No Response)

Reviewer #7: All comments have been addressed

Reviewer #9: All comments have been addressed

**********

2. Is the manuscript technically sound, and do the data support the conclusions?

Reviewer #4: (No Response)

Reviewer #7: Yes

Reviewer #9: Yes

**********

3. Has the statistical analysis been performed appropriately and rigorously?

Reviewer #4: (No Response)

Reviewer #7: Yes

Reviewer #9: Yes

**********

4. Have the authors made all data underlying the findings in their manuscript fully available?

The PLOS Data policy

Reviewer #4: (No Response)

Reviewer #7: Yes

Reviewer #9: Yes

**********

5. Is the manuscript presented in an intelligible fashion and written in standard English?

Reviewer #4: (No Response)

Reviewer #7: Yes

Reviewer #9: Yes

**********

Reviewer #4: The bioassay data for the allelopathic potential of the extract are limited to morphological traits, which represent only a routine assessment. This study should include sufficient physiological and biochemical analyses to elucidate the underlying inhibitory mechanisms.

Reviewer #7: Thank you for submitting the revised version appropriately. I am satisfied with the revised version of the paper and recommend it for acceptance.

Reviewer #9: The MS has been reviewed and authors made necessary corrections. It is suitable to be published in PlosOne

**********

what does this mean?). If published, this will include your full peer review and any attached files.

If you choose “no”, your identity will remain anonymous but your review may still be made public.

**Do you want your identity to be public for this peer review?**  For information about this choice, including consent withdrawal, please see our Privacy Policy

Reviewer #4: **Yes:** Mohammad Pouresmaeil

Reviewer #7: No

Reviewer #9: No

**********

# Editor report: Acceptance letter

**DOI:** 10.1371/journal.pone.0354454.r010

**Authors:** Ahmet Uludag

PONE-D-25-29786R4

PLOS One

Dear Dr. Alebrahim,

I'm pleased to inform you that your manuscript has been deemed suitable for publication in PLOS One. Congratulations! Your manuscript is now being handed over to our production team.

At this stage, our production department will prepare your paper for publication. This includes ensuring the following:

* All references, tables, and figures are properly cited

* All relevant supporting information is included in the manuscript submission,

* There are no issues that prevent the paper from being properly typeset

You will receive further instructions from the production team, including instructions on how to review your proof when it is ready. Please keep in mind that we are working through a large volume of accepted articles, so please give us a few days to review your paper and let you know the next and final steps.

Lastly, if your institution or institutions have a press office, please let them know about your upcoming paper now to help maximize its impact. If they'll be preparing press materials, please inform our press team within the next 48 hours. Your manuscript will remain under strict press embargo until 2 pm Eastern Time on the date of publication. For more information, please contact onepress@plos.org.

You will receive an invoice from PLOS for your publication fee after your manuscript has reached the completed accept phase. If you receive an email requesting payment before acceptance or for any other service, this may be a phishing scheme. Learn how to identify phishing emails and protect your accounts at https://explore.plos.org/phishing.

If we can help with anything else, please email us at customercare@plos.org.

Thank you for submitting your work to PLOS ONE and supporting open access.

Kind regards,

PLOS ONE Editorial Office Staff

on behalf of

Dr. Ahmet Uludag

Academic Editor

PLOS One
